# One Scaffold, Three Chelation Modes: 2‑(1*H*‑Tetrazol-1-yl)pyridine as a Dynamic Ligand for Cationic and Neutral Cyclometalated Iridium(III) Complexes

**DOI:** 10.1021/acs.inorgchem.6c02199

**Published:** 2026-08-20

**Authors:** Cecilia Bruschi, Dmitry Tungulin, Filippo Monti, Elia Matteucci, Enrico Leoni, Letizia Sambri, Nicola Armaroli, Andrea Baschieri

**Affiliations:** † Laboratory of Organic Electronics, Department of Science and Technology, Linköping University, 601 74 Norrköping, Sweden; ‡ Department of Industrial Chemistry, TosoMontanari, University of Bologna, Via Piero Gobetti 85, 40129 Bologna, Italy; § Department of Chemistry, Giacomo Ciamician, University of Bologna, Via Piero Gobetti 85, 40129 Bologna, Italy; ∥ Laboratorio TIMAS-MCC, ENEA, Via Ravegnana 186, 48018 Faenza (RA), Italy; ⊥ Institute for Organic Synthesis and Photoreactivity (ISOF), National Research Council of Italy (CNR), Via Piero Gobetti 101, 40129 Bologna, Italy

## Abstract

The 2-(1*H*-tetrazol-1-yl)­pyridine scaffold (**L1**) is exploited as a versatile ligand in heteroleptic iridium­(III) complexes. Depending on the reaction conditions, ligand **L1** reacts with the prototypical iridium­(III) dimer [Ir­(ppy)_2_(μ-Cl)]_2_ (where Hppy = 2-phenylpyridine) leading to the formation of distinct complexes. As a neutral N^∧^N ancillary ligand, it forms the cationic complex **A**, otherwise, after deprotonation of its tetrazole ring, as a monoanionic C^∧^N ligand it can lead the neutral complex **B**. Finally, after *N*-methylation to form the tetrazolium salt **L2**, it behaves as a *N*-heterocyclic carbene (N^∧^C: chelation mode), forming the cationic complex **C**. This trimodal coordination versatility is further enriched by the serendipitous discovery of the byproduct complex **D**, arising from partial ring-opening of the tetrazolium salt. The electrochemical redox gap of the complexes reflects the different σ-donor and π-acceptor character of each coordination mode. Complex **A** shows a broad metal-to-ligand charge-transfer (^3^MLCT) emission band at 298 K, while complexes **B**–**D** display an intricate interplay between ligand-centered (^3^LC) and ^3^MLCT states, as confirmed by DFT calculations. This work establishes a novel class of multifunctional ligands and provides a comprehensive structure–property investigation for improving the photophysical performances of tetrazole-based iridium­(III) emitters for optoelectronic applications.

## Graphical abstract

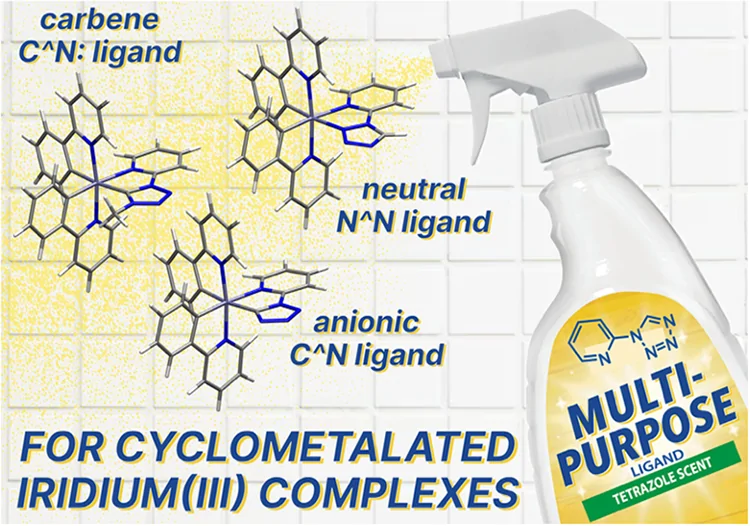

## Introduction

Cyclometalated iridium­(III) complexes have attracted considerable attention owing to a distinctive combination of properties: they behave as efficient phosphorescent emitters, generally characterized by high quantum yields together with robust photochemical and photophysical stability.^1^ In addition, appropriate selection of the coordinating ligands allows fine control over the emission wavelength across the entire visible range.^2^ These favorable characteristics have enabled the use of such complexes in a variety of application fields, spanning from luminescent materials^3^ to bioimaging.^4,5^

Heteroleptic iridium­(III) complexes are typically built around two bidentate cyclometalating ligands with a third ancillary ligand completing the octahedral coordination sphere of the metal ion. Depending on the nature of this ancillary ligand (*i*.*e*., neutral, dianionic, or monoanionic), the overall complex can be cationic, anionic, or neutral, respectively. Within this broad ligand landscape, iridium complexes bearing phenyl-substituted *N*-heterocyclic carbene ligands have been the subject of extensive investigation over the past few decades.^6–15^

As mentioned above, the emission wavelength of iridium­(III) complexes can be adjusted through chemical modification of the ligands, perturbing the energy of the frontier molecular orbitals and consequently the HOMO–LUMO gap. One approach for tuning the emission color is based on the addition of electron-rich atoms to the ligand scaffold.

In this perspective, Ortí and co-workers reported a purely computational DFT study systematically investigating how the number and position of nitrogen atoms in an azole ring in both the cyclometalating and ancillary ligands could affect the electronic structure and phosphorescent emission energy of cationic [Ir­(C^∧^N)_2_(N^∧^N)]^+^ complexes.^16^ They found that, when the azole ring is incorporated into the cyclometalating C^∧^N ligands, increasing the number of nitrogen atoms on the ring preferentially stabilizes the HOMO, which is localized on those ligands and on the iridium d_π_ orbitals, leading to a widening of the HOMO–LUMO gap and a progressive blue-shift of the emission. This theoretical evidence found its experimental validation in the literature, considering complexes of the general formula [Ir­(C^∧^N)_2_(bpy)]^+^, where bpy = 2,2′-bipyridine and C^∧^N is a phenyl-azole with increasing nitrogen content (*i*.*e*., pyrazole,^17^ triazole,^18^ and tetrazole^19^ ring). Conversely, when the azole moiety is part of the ancillary N^∧^N ligand, the LUMO (which is centered on the π* orbitals of such a ligand) is stabilized to a greater extent than the HOMO, resulting in a narrowing of the HOMO–LUMO gap and in a corresponding red shift of the emission as the number of nitrogen atoms increases.^16^

Calculations also revealed that the nature of the atom (*i.e.*, carbon or nitrogen) of the azole ring directly linked to the phenyl substituent of the cyclometalating C^∧^N ligands plays a decisive role: complexes bearing a nitrogen atom at that position display a markedly larger HOMO–LUMO gap compared to structural isomers with a carbon atom in the same position, even when the total number of ring nitrogen atoms is identical.^16^ Anyway, such a study did not investigate such an effect on the ancillary one.

Pyridyl-tetrazole derivatives have been extensively used as ancillary ligands in cyclometalated iridium­(III) complexes, even in combination with the archetypal [Ir­(ppy)_2_(N^∧^N)]^0/+^ structures.^20,21^ Anyway, such examples always report pyridyl-tetrazoles with the azole ring linked to the pyridine one through the tetrazolyl carbon, which is not the case explored by Ortí and co-workers.^16^

With the scope of exploring the positional role of nitrogen atoms on the pyridyl-tetrazole ancillary ligand, we synthesized a new family of emitting iridium­(III) complexes using 2-(1*H*-tetrazol-1-yl)­pyridine (**L1**) as a chelating ligand (Chart 1), so a scaffold in which the tetrazole ring is linked to the pyridine one through a tetrazolyl nitrogen. Beyond mere electronic and optical perturbations due to positional effects, this unit can also be used either as a neutral coordinating N^∧^N ligand or, upon deprotonation under basic conditions, as a monoanionic C^∧^N ligand (Chart 1).

**Chart 1:**
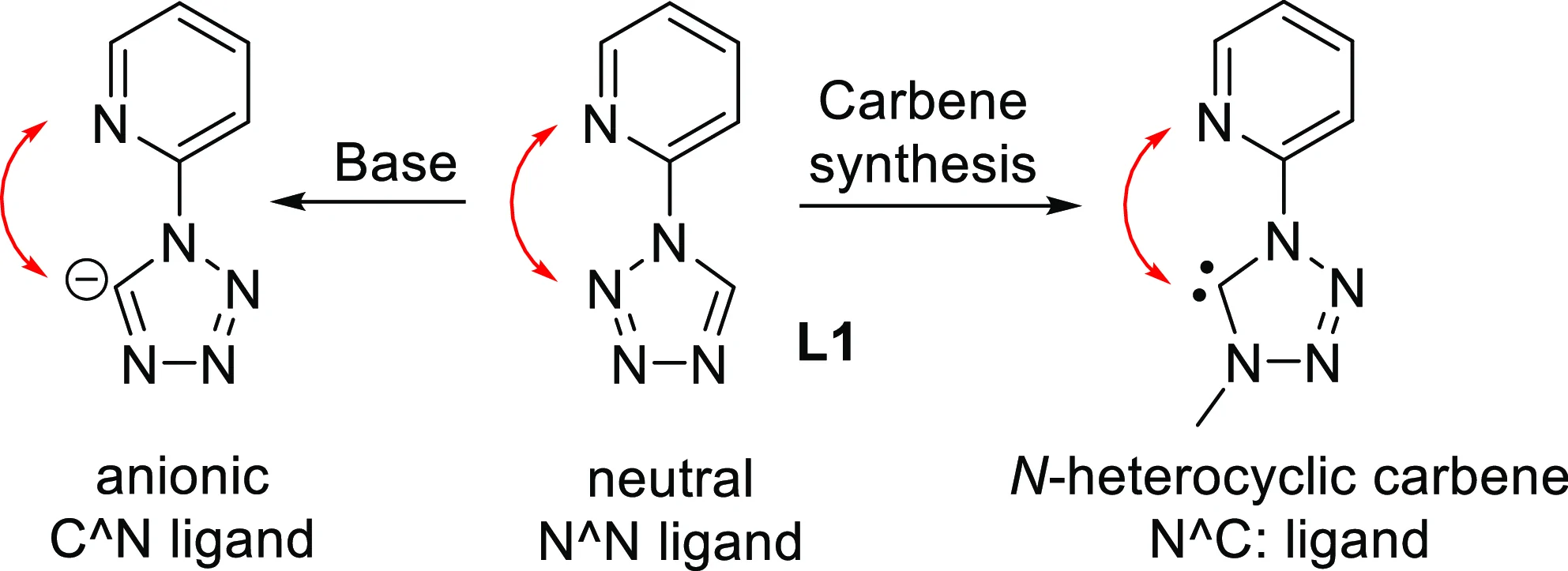
Versatility of 2-(1*H*-tetrazol-1-yl)­pyridine Derivatives as Bidentate Ligands[Fn c1fn1]

a The coordination sites are pointed out.

In the past, several attempts were explored to use **L1** as a ligand in transition-metal complexes. As an example, in 1995, Downard et al. unsuccessfully tried the synthesis of palladium­(II) and ruthenium­(II) complexes bearing **L1**.^22^ Only in 2020, Ivanova and co-workers reported the synthesis of the first organometallic compounds containing this ligand.^23^ They were complexes based on copper­(II), cobalt­(II), and nickel­(II). In all these cases, **L1** was used as a monodentate ligand, and the bond to the metal center was formed only via a single nitrogen atom of the tetrazole ring. However, these compounds were not emissive.^23^

For the first time, the present study successfully explores the versatility of this unit as a chelating ligand (Chart 1), preparing three distinct families of luminescent heteroleptic iridium­(III) complexes: **L1** was used either (i) as a N^∧^N ancillary ligand, affording a cationic complex **A**, or (ii) as a cyclometalating C^∧^N ligand, yielding a neutral complex **B**; otherwise (iii) to get the corresponding *N*-heterocyclic carbenes (**L2**), which was subsequently used as a N^∧^C: ligand, obtaining the cationic complex **C** (Scheme 1).

**Scheme 1:**
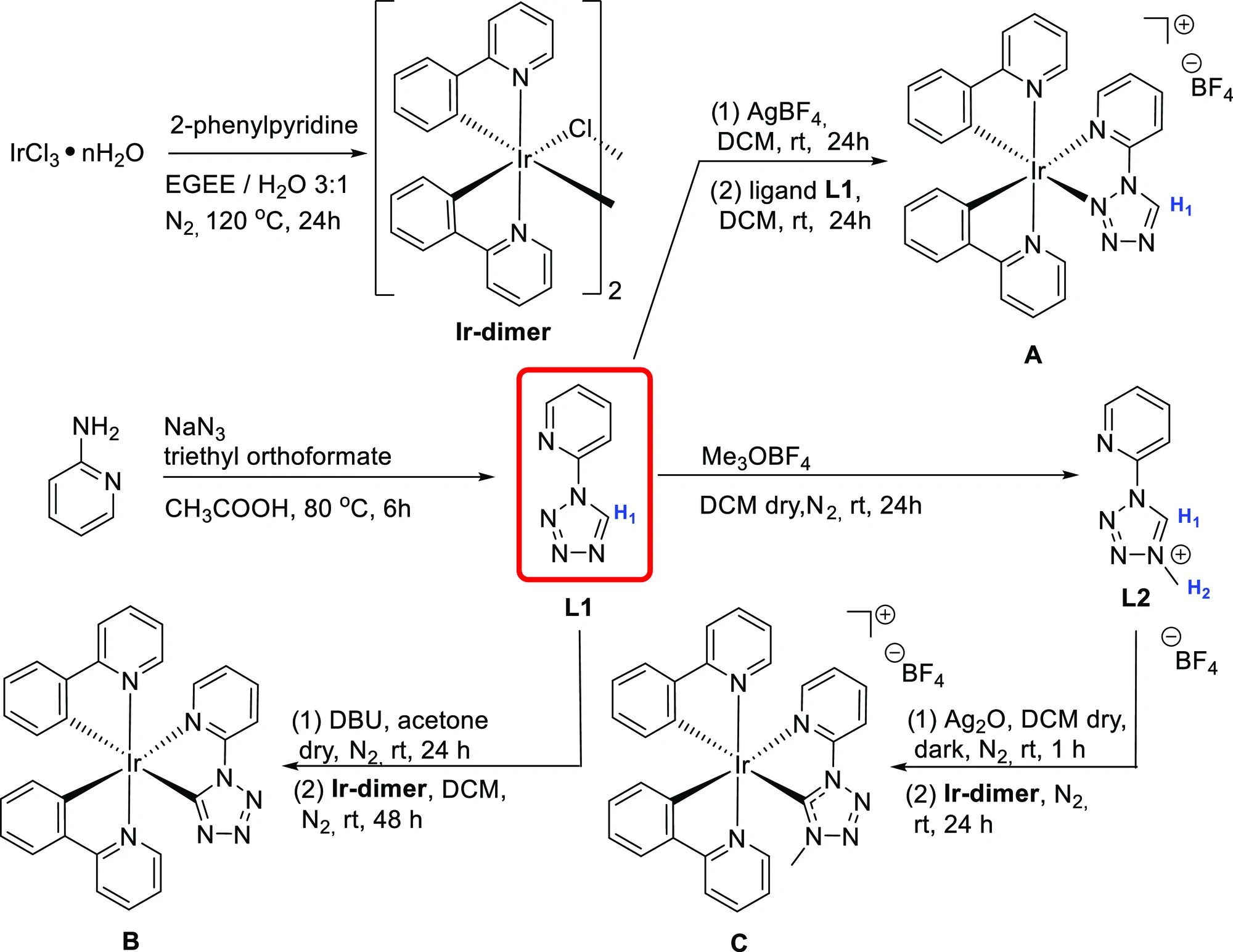
Synthesis of Tetrazole **L1** and the Methylated Tetrazolylidene **L2** Used as Ligands and of the Related Complexes (A–C)

## Experimental Section

### General Information

Analytical-grade solvents and commercially available reagents were used as received unless otherwise stated. Chromatographic purifications were performed using 70–230 mesh silica gel and 70–290 mesh Al_2_O_3_. ^1^H, ^13^C, and ^19^F NMR spectra were recorded on a Varian AS 300, on a Mercury 400 or a Varian AS 600 spectrometer. All spectra were acquired at 25 °C, unless otherwise specified.

Chemical shifts (δ) are reported in ppm relative to residual solvent signals for ^1^H and ^13^C NMR (^1^H NMR: 7.26 ppm for CDCl_3_, 2.05 ppm for acetone-d_6_,1.94 ppm for CD_3_CN; ^13^C NMR: 77.0 ppm for CDCl_3_, 29.9 ppm for acetone-d_6_,1.32 ppm for CD_3_CN). ^19^F NMR spectra were recorded at 470 MHz using trichlorofluoromethane as an external standard. ^13^C­{^1^H} NMR spectra were acquired with ^1^H broad band decoupled mode. Coupling constants are given in Hz. The types of carbon atoms were determined by DEPT NMR experiments. To indicate the multiplicity were used the following abbreviations: s, singlet; d, doublet; t, triplet; dd, double doublet; dt, double triplet; m, multiplet. The high-resolution mass spectra (HRMS) were obtained with an ESI-QTOF (Agilent Technologies, model G6520A) instrument, and the *m*/*z* values are referred to the monoisotopic mass.

***Caution*** **:** *These nitrogen-rich compounds posed no handling difficulties in our experience, though small-scale work and best safety practices are strongly advised*. *Handling NaN*_3_ *requires particular care*: *it is fatal upon ingestion*, *skin contact*, *or inhalation*, *and prolonged or repeated ingestion may cause organ damage (brain)*. *It is also very toxic to aquatic life with long-lasting effects*.

#### Ir-Dimer

[Ir­(ppy)_2_(μ-Cl)]_2_ was synthesized following a previously reported procedure with slight modifications.^24^ IrCl_3_ · nH_2_O (50 mg, 0.167 mmol, 1 equiv) and 2-phenylpyridine (65 mg, 0.418 mmol, 2.5 equiv) were dissolved in a 3:1 mixture of 2-ethoxyethanol and H_2_O (4 mL) under N_2_ atmosphere. The solution was refluxed overnight. After this time, water (5 mL) was added and the formed yellow solid was filtered on Büchner and washed with water (2 × 5 mL), Et_2_O (2 × 5 mL), and hexane (2 × 5 mL). The crude dimer was used in the next step without further purifications (63.7 mg; yield 71.3%).

#### Synthesis of 2-(1*H*-Tetrazol-1-yl)­pyridine (**L1**)

Compound **L1** was synthesized following a previously reported procedure with slight modifications.^25^ 2-Aminopyridine (0.94 g, 10 mmol, 1 equiv), sodium azide (1.33 g, 20 mmol, 2 equiv), and triethyl orthoformate (5 mL, 30 mmol, 3 equiv) were dissolved in glacial acetic acid (4.6 mL, 80 mmol, 8 equiv). The solution was stirred at 80 °C for 6h. Acetic acid was removed in vacuum, and the crude was dissolved in Et_2_O. NaHCO_3_ was added until the evolution of CO_2_ ceased. The formed solid was filtered off, and the ether layer was dried over Na_2_SO_4_. The solvent was removed in vacuum, and the crude was recrystallized from EtOH. The final product was obtained in 62% yield (0.91 g) as white crystals. NMR spectra were in agreement with those available in the literature data.^26^ ^1^H NMR (400 MHz, CDCl_3_) δ 9.53 (s, 1H, H_1_), 8.53 (ddd, *J* = 4.9, 1.9, 0.9 Hz, 1H), 8.07 (d, *J* = 8.2 Hz, 1H), 8.03 – 7.92 (m, 1H), 7.44 (ddd, *J* = 7.4, 4.9, 1.1 Hz, 1H); ^13^C {^1^H} NMR (101 MHz, CDCl_3_) δ 149.0 (CH), 146.9 (C), 140.0 (CH), 139.7 (CH), 124.7 (CH), 114.3 (CH); HRMS (ESI-QTOF) ([M + Na]^+^): *m*/*z* calcd for C_6_H_5_N_5_: 170.0443; found: 170.0442.

#### Synthesis of 4-Methyl-1-(pyridin-2-yl)-1*H*-tetrazol-4-ium Tetrafluoroborate (**L2**)

In a double-necked round-bottom flask, compound **L1** (300 mg, 2.04 mmol, 1 equiv) was dissolved in dichloromethane dry (18 mL), and Me_3_OBF_4_ (368 mg, 2.49 mmol, 1.2 equiv) was added to the solution. The mixture was stirred for 24 h under a nitrogen atmosphere. Then, the solvent was removed, and the crude was washed with ethylic ether (2 × 5 mL) and dichloromethane (5 mL). The final product was obtained in 93% yield (479 mg, 1.89 mmol) as a white powder. ^1^H NMR (400 MHz, CD_3_CN) δ 10.81 (s, 1H, H_1_), 8.69 (ddd, *J* = 4.8, 1.8, 0.9 Hz, 1H), 8.27 – 8.22 (m, 1H), 8.16 (dt, *J* = 8.3, 1.0 Hz, 1H), 7.76 (ddd, *J* = 7.5, 4.9, 1.1 Hz, 1H), 4.45 (s, 3H, H_2_); ^13^C {^1^H} NMR (101 MHz, CD_3_CN) δ 151.0 (CH), 146.1 (CH), 142.2 (CH), 141.1 (C), 128.8 (CH), 116.2 (CH), 39.2 (CH_3_, C_2_); ^19^F NMR (282 MHz, CD_3_CN) δ −151.8; HRMS (ESI-QTOF) ([M]^+^): *m*/*z* calcd for C_7_H_8_N_5_^+^: 162.0775; found: 162.0781.

#### Synthesis of Complex **A**

In a 25 mL round-bottom flask, **Ir-dimer** (30 mg, 0.028 mmol, 1 equiv) was dissolved in dichloromethane (3 mL) and ethanol (2 drops). When the solid was dissolved, AgBF_4_ (10.9 mg, 0.056 mmol, 2 equiv) was added. The mixture was kept in the dark and stirred at room temperature overnight. After this period, the stirring was stopped and the formed AgCl salt was removed. The yellow solution was added dropwise to ligand **L1** (8.2 mg, 0.056 mmol, 2 equiv), previously dissolved in dichloromethane (8 mL). The mixture was stirred at room temperature for a further 24 h. Then the solvent was removed, the solid was dissolved in acetone 5 mL and recrystallized with ethylic ether. Complex **A** was isolated as a pure yellow solid in 41% yield (16.9 mg, 0.023 mmol). ^1^H NMR (400 MHz, acetone-d_6_) δ 10.72 (s, 1H, H_1_), 8.80 (dt, *J*_D_ = 8.2 Hz, *J*_T_ = 0.9 Hz, 1H), 8.56 – 8.50 (m, 1H), 8.26 (t, *J* = 8.4 Hz, 2H), 8.06 – 7.98 (m, 4H), 7.93 – 7.89 (m, 2H), 7.88 – 7.81 (m, 2H), 7.21 (t, *J* = 7.4 Hz, 1H), 7.16 (t, *J* = 7.4 Hz, 1H), 7.08 (dt, *J*_T_ = 7.5 Hz, *J*_D_ = 1.6 Hz 1H), 7.00 (dt, *J*_T_ = 7.5 Hz, *J*_D_ = 1.6 Hz 1H), 6.95 (dt, *J*_T_ = 7.5 Hz, *J*_D_ = 1.6 Hz 1H), 6.88 (dt, *J*_T_ = 7.5 Hz, *J*_D_ = 1.6 Hz 1H), 6.31 (t, *J* = 7.9 Hz, 2H); ^13^C {^1^H} NMR (151 MHz, acetone-d_6_) δ 168.7 (C), 167.7 (C), 151.4 (CH), 151.0 (CH), 150.7 (CH), 147.0 (C), 146.6 (C), 145.2 (C), 145.0 (C), 144.6 (C), 143.6 (CH), 140.1_8_ (CH), 140.1_6_ (CH), 132.7 (CH), 132.1 (CH), 131.4 (CH), 130.7 (CH), 129.3_2_ (CH), 129.3_1_ (CH), 126.0 (CH), 125.5 (CH), 124.8 (CH), 124.4 (CH), 124.2 (CH), 123.7 (CH), 121.0 (CH), 120.9 (CH), 117.6 (CH); ^19^F NMR (376 MHz, acetone-d_6_) δ −152.00; HRMS (ESI-QTOF) ([M]^+^): *m*/*z* calcd for C_28_H_21_IrN_7_^+^: 648.1484; found: 648.1463.

#### Synthesis of Complex **B**

In a 25 mL double-necked round-bottom flask, ligand **L1** (8.6 mg, 0.058 mmol, 1 equiv) was dissolved in freshly distilled dry acetone (8 mL). DBU (44 μL, 0.29 mmol, 5 equiv) was added and the reaction was stirred under nitrogen at room temperature for 24 h. **Ir-dimer** (31.3 mg, 0.029 mmol, 0.5 equiv) was dissolved in dry dichloromethane and the yellow solution was added dropwise to deprotonated ligand **L1**. The mixture was stirred under a nitrogen atmosphere at room temperature for a further 48 h. The solvent was removed and the crude was washed with hexane. Then the solid was dissolved in acetone 5 mL and recrystallized with ethylic ether. Complex **B** was isolated as a pure yellow solid in 27.6% yield (10.3 mg, 0.016 mmol). ^1^H NMR (400 MHz, CDCl_3_) δ 8.99 (d, *J* = 8.2 Hz, 1H), 8.00 (d, *J* = 5.8 Hz, 1H), 7.91 (d, *J* = 8.1 Hz, 1H), 7.86 (d, *J* = 7.9 Hz, 1H), 7.81 – 7.72 (m, 2H), 7.60 – 7.48 (m, 3H), 7.44 (d, *J* = 8.1 Hz, 1H), 7.23 – 7.18 (m, 1H), 7.07 – 7.02 (m, 1H), 6.89 – 6.80 (m, 3H), 6.74 – 6.66 (m, 2H), 6.63 – 6.56 (m, 1H), 6.21 (d, *J* = 8.9 Hz, 1H), 6.15 (d, *J* = 7.1 Hz, 1H); ^13^C {^1^H} NMR (151 MHz, CDCl_3_) δ 169.6 (C), 169.0 (C), 167.8 (C), 151.1 (CH), 149.2 (CH), 148.2 (C), 144.6 (CH), 144.1_3_ (C), 144.1_1_ (C), 143.7 (C), 138.3 (CH), 137.4 (CH), 137.1 (CH), 132.3 (CH), 132.1 (CH), 129.6 (CH), 129.5 (CH), 124.2 (CH), 124.0 (CH), 122.7 (CH), 122.3 (CH), 121.6 (CH), 121.3 (CH), 119.0 (CH), 118.6 (CH), 118.4 (C), 114.6 (CH), 113.4 (CH); HRMS (ESI-QTOF) ([M + H]^+^): *m*/*z* calcd for C_28_H_20_IrN_7_^+^: 646.1464; found: 646.1463.

#### Synthesis of Complex **C** and **D**

In a 25 mL double-necked round-bottom flask, ligand **L2** (9.8 mg, 0.04 mmol, 2.1 equiv) was dissolved in dry dichloromethane (8 mL). Ag_2_O (9.5 mg, 0.04 mmol, 2.2 equiv) was added in the absence of light and the mixture was kept in the dark and stirred under nitrogen at room temperature for 1 h. After this period, **Ir-dimer** (20 mg, 0.019 mmol, 1 equiv) was added. The mixture was stirred under a nitrogen atmosphere at room temperature for a further 24 h. The solid was removed with a Celite pad and the crude precipitate was washed with ethylic ether (3 × 5 mL). Then, the solvent was removed, and the crude was purified by column chromatography on Al_2_O_3_ using DCM/MeOH 97:3 as an eluent, to give complex **C** as a pure yellow solid in 31.8% yield (8.9 mg, 0.012 mmol). ^1^H NMR (300 MHz, CDCl_3_) δ 8.45 (d, *J* = 8.4 Hz, 1H), 8.28 – 8.20 (m, 1H), 8.15 (d, *J* = 5.1 Hz, 1H), 7.96 – 7.84 (m, 2H), 7.84 – 7.64 (m, 5H), 7.55 (d, *J* = 5.9 Hz, 1H), 7.49 – 7.41 (m, 1H), 7.23 – 7.17 (m, 1H), 7.09 (d, *J* = 16.4 Hz, 2H), 7.04 – 6.96 (m, 2H), 6.91 (td, *J* = 7.4, 1.4 Hz, 1H), 6.46 (dd, *J* = 7.6, 1.0 Hz, 1H), 6.23 (dd, *J* = 7.3, 1.0 Hz, 1H), 3.57 (s, 3H); ^13^C {^1^H} NMR (101 MHz, CDCl_3_) δ 172.3 (C), 167.7 (C), 167.2 (C), 160.7 (C), 154.77 (CH), 149.9 (CH), 149.5 (C), 149.2 (CH), 144.5 (C), 144.2 (C), 142.5 (C), 142.4 (CH), 138.4 (CH), 137.9 (CH), 131.2 (CH), 130.9 (2 CH), 130.7 (CH), 126.7 (CH), 125.0 (CH), 124.7 (CH), 124.6 (CH), 123.6 (CH), 123.6 (CH), 122.3 (CH), 119.7 (CH), 119.6 (CH), 115.0 (CH), 36.6 (CH_3_); ^19^F NMR (282 MHz, CDCl_3_) δ −153.72; HRMS (ESI-QTOF) ([M]^+^): *m*/*z* calcd for C_29_H_23_IrN_7_^+^: 662.1640; found: 662.1620.

#### Complex **D**

Results: 4.5 mg as a yellow solid, 0.007 mmol, yield = 19%. ^1^H NMR (600 MHz, CDCl_3_) δ 9.06 (s, 1H, H_1_), 8.66 (d, *J* = 5.7 Hz, 1H), 8.04 (d, *J* = 5.9 Hz, 1H), 7.96 (d, *J* = 8.2 Hz, 1H), 7.89 (d, *J* = 8.4 Hz, 1H), 7.86 – 7.81 (m, 2H), 7.70 – 7.63 (m, 2H), 7.55 (d, *J* = 9.2 Hz, 1H), 7.32 (dd, *J* = 16.9, 8.1 Hz, 2H), 7.21 (t, *J* = 6.6 Hz, 1H), 7.18 – 7.12 (m, 1H), 6.95 – 6.86 (m, 2H), 6.78 – 6.65 (m, 4H), 6.23 (d, *J* = 8.9 Hz, 1H), 6.07 (d, *J* = 7.6 Hz, 1H), 2.51 (d, *J* = 4.7 Hz, 3H, H_2_); ^13^C {^1^H} NMR (151 MHz, CDCl_3_) δ 168.5 (C), 167.1 (C), 157.0 (C), 150.4 (C), 149.5 (CH), 148.9 (CH), 148.3 (CH), 146.6 (C), 144.3 (C), 143.7 (C), 143.5 (C), 139.9 (CH), 138.3 (CH), 137.9 (CH), 132.6 (CH), 132.4 (CH), 130.1 (CH), 129.5 (CH), 124.9 (CH), 123.9 (CH), 122.7 (CH), 122.4 (CH), 122.3 (CH), 121.7 (CH), 120.4 (CH), 119.2 (CH), 119.0 (CH), 117.0 (CH), 27.3 (CH_3_, C_2_); ESI-MS: 634 [M + H]^+^; HRMS (ESI-QTOF) ([M+K]^+^): *m*/*z* calcd for C_29_H_24_IrN_5_^+^: 672.1275; found: 672.1273.

#### (*E*)-*N*-Methyl-*N*’-(pyridin-2-yl)­formimidamide (**L3**)

^1^H NMR (400 MHz, CD_3_CN) δ 9.23 (s, 1H), 8.37 – 8.12 (m, 2H), 7.37 (t, *J* = 7.4 Hz, 1H), 7.25 (d, *J* = 8.9 Hz, 1H), 6.10 (s, 1H), 2.83 (d, *J* = 4.7 Hz, 3H). HRMS (ESI-QTOF) ([M]^+^): *m*/*z* calcd for C_7_H_9_N_3_^+^: 135.0796; found: 135.0796.

### Electrochemical Characterization

Voltammetric measurements were conducted with a Metrohm AutoLab PGSTAT 302N electrochemical workstation controlled via the NOVA 2.0 software. All experiments were performed at room temperature in acetonitrile or dichloromethane, with sample concentrations of approximately 0.5 mM and 0.1 M tetrabutylammonium hexafluorophosphate (electrochemical grade, TBAPF_6_) employed as the supporting electrolyte. Argon bubbling was used to remove oxygen from the solutions. A three-electrode configuration was adopted throughout (BioLogic VC-4 cell, volume range 1–3 mL), consisting of a glassy carbon working electrode (1.6 mm diameter active disk surface), an Ag/AgNO_3_ reference electrode (0.01 M in acetonitrile, 0.1 M TBAClO_4_ supporting electrolyte), and a platinum wire counter electrode. Ferrocene was added as an internal reference at the conclusion of each measurement. Cyclic voltammetry (CV) scans were acquired at 100 mV s^–1^, while Osteryoung square-wave voltammetry (OSWV) scans were recorded at 25 mV s^–1^, with a square-wave amplitude of ± 20 mV and a frequency of 25 Hz.

### Photophysics

Spectroscopic measurements were performed in spectrofluorimetric-grade acetonitrile and dichloromethane. Absorption spectra were acquired using a PerkinElmer Lambda 950 spectrophotometer. For photoluminescence measurements, sample solutions were held in 10.00 mm fluorimetric Suprasil quartz cuvettes, with dissolved oxygen removed by argon bubbling for 30 min. Uncorrected emission spectra were collected with an Edinburgh Instruments FLS920 spectrometer, equipped with a Peltier-cooled Hamamatsu R928 photomultiplier tube (PMT, spectral window 185–850 nm), using an Osram XBO xenon arc lamp (450 W) as excitation source. Spectral correction was achieved through a calibration curve obtained with an Ocean Optics calibrated deuterium–halogen lamp (DH-3plus-CAL-EXT). Photoluminescence quantum yields (PLQYs) in solution were determined from the corrected spectra on a wavelength (nm) scale, following the method of Demas and Crosby,^27^ with an air-equilibrated aqueous solution of tris­(2,2′-bipyridyl)­ruthenium­(II) dichloride used as reference standard (PLQY = 0.040).^28^ Emission lifetimes (τ) were obtained via the time-correlated single photon counting (TCSPC) technique, using an HORIBA Jobin Yvon IBH FluoroHub controlling a spectrometer equipped with a pulsed NanoLED or SpectraLED (λ_exc_ = 370 nm for both) as excitation source and a red-sensitive Hamamatsu R-3237–01 PMT (185–850 nm) as detector. Luminescence decay profiles were analyzed with the manufacturer-supplied DAS6 Decay Analysis Software, and fit quality was evaluated based on χ^2^ values close to unity and residuals evenly distributed across the time axis. Luminescence spectra at 77 K were recorded on sample solutions loaded into quartz tubes (2 mm inner diameter) immersed in a dedicated quartz Dewar flask filled with liquid nitrogen. Estimated experimental uncertainties are ± 8% for τ, ± 10% for PLQYs, and ±2 nm and ±5 nm for absorption and emission peaks, respectively.

### Computational Details

Density functional theory (DFT) calculations were performed with the Gaussian 16 program package (revision B.01),^29^ employing the M06 global-hybrid meta-GGA exchange-correlation functional.^30,31^ For the iridium metal center, the first 60 inner-core electrons were replaced by the fully relativistic Stuttgart/Cologne energy-consistent pseudopotential with multielectron fit (ECP60MDF), combined with the corresponding triple-ζ basis set (*i*.*e*., cc-pVTZ-PP basis).^32^ For all remaining atoms, the Pople 6–31G­(d,p) basis set was employed.^33,34^ Full geometry optimizations, without symmetry constraints, were carried out at the time-independent DFT level for both the ground state (S_0_) and the lowest triplet state of all complexes examined; acetonitrile solvation effects were accounted for throughout via the polarizable continuum model (PCM).^35,36^ Frequency calculations were systematically performed to verify that each optimized stationary point corresponded to a genuine minimum on the potential-energy surface (absence of imaginary frequencies). The nature of the emitting states was probed through geometry optimizations and frequency calculations at the spin-unrestricted UM06 level (spin multiplicity of 3), starting either from the S_0_-optimized geometry or from alternative educated guesses. ^3^MC states were likewise optimized within the spin-unrestricted DFT framework, testing multiple starting geometries generated primarily by elongating selected Ir–N bonds and tilting the corresponding aromatic ring. Time-dependent DFT calculations (TD-DFT),^37,38^ performed at the same level of theory as the geometry optimizations, were used to compute the first 16 triplet excited states from the S_0_ minimum, with their character assigned through Natural Transition Orbital (NTO) analysis.^39^ All the pictures reporting molecular geometries, orbitals, and spin-density surfaces were visualized using GaussView 6.^40^

## Results and Discussion

### Synthesis

The [Ir­(ppy)_2_(μ-Cl)]_2_ dimer was synthesized following the general procedure reported by Nonoyama in 1974.^24^ IrCl_3_ hydrate was reacted with two equivalents of 2-phenylpyridine (Hppy), selected as a cyclometalating ligand, giving the **Ir-dimer**, as reported in Scheme 1.

Subsequently, following the procedure reported by Linert,^41^ 2-aminopyridine, sodium azide and triethyl orthoformate were dissolved in glacial acetic acid and the solution was stirred at 80 °C for 6 h. The final product **L1** was obtained in 62% yield as a white solid. NMR signals were in accordance with literature-reported data. The signal related to the proton of the tetrazole ring appears as a singlet centered at 9.53 ppm (H_1_ in **L1**), as reported in Figures S1–S3.

Then, **L2** was synthesized using **L1** as the starting material. To get the tetrazolium salt precursors of tetrazolylidenes, Me_3_OBF_4_ was used as the methylating agent. The reaction was performed in dichloromethane at room temperature under a nitrogen atmosphere. Ligand **L2** was obtained in 93% yield. The ^1^H NMR spectrum of the product confirms the expected structure, showing the appearance of a new singlet of an *N*-methyl group at 4.45 ppm (H_2_ in **L2**), while the tetrazole proton signal remains unchanged (H_1_ in **L2**) (Figures S4–S7). HRMS analysis was performed for ligands **L1** and **L2** (Figures S8 and S9).

To elucidate the structure of ligand **L2**, several crystallization attempts were carried out by using different solvents. Unfortunately, none of these attempts yielded crystals that were suitable for X-ray diffraction analysis. During efforts to recover **L2** from these experiments, ligand **L3** was identified as a degradation product. Tetrazolylidenes bearing unsubstituted nitrogen atoms (N2 and N3) are known to undergo decomposition, releasing molecular nitrogen, as previously reported for related heterocycles.^42,43^

The resulting compound was characterized by NMR spectroscopy and HRMS analysis (Figures S10 and S11). These findings provide indirect evidence that methylation occurred at the 1,4-positions of the tetrazolium ring, consistent with previous literature reports.^42,44^ Any minor amounts of the corresponding 1,3-substituted isomers were removed during the purification process.

All the obtained precursors were then used to prepare the luminescent complexes (**A**–**C**). Initially, the **Ir-dimer** was treated with AgBF_4_ to increase its reactivity and then added to a solution of ligand **L1**, previously dissolved in dichloromethane. The reaction mixture was stirred at room temperature for 24 h. Finally, recrystallization from ethylic ether in acetone gave the pure complex **A** in good yield (41%). The formation of the desired complex was confirmed by NMR spectrometry (Figures S12–S15). In detail, in the ^1^H NMR spectrum, the singlet of the CH of the tetrazole ring was shifted to 10.72 ppm (H_1_ in **A**), while the signals associated with the two cyclometalated ligands are not equivalent, confirming the nonsymmetric structure of the resulting complex. The ^13^C NMR spectrum was consistent with the proposed structure, displaying signals for all nonequivalent carbon atoms.

For the neutral iridium­(III) complex **B**, the development of a new synthetic methodology was required, as the 2-(1*H*-tetrazol-1-yl)­pyridine acts as a C^∧^N ligand. A key challenge in using **L1** as a cyclometalating ligand lies in identifying a suitable base to promote the reaction, since the tetrazolylpyridine **L1** exhibits pronounced pH sensitivity. Therefore, a systematic screening of different organic bases was performed. In the presence of a strong base, such as tetrabutylammonium hydroxide (TBAOH), **L1** reacted to form a new species, i.e., *N*-(pyridin-2-yl)­cyanamide. As soon as the base was added to the solution, bubbles of N_2_ were observed. The singlet associated with the tetrazole ring disappeared, while a new broad singlet at 5.30 ppm, attributable to a newly formed amine group, appeared (Figure S16). In contrast, in the presence of trimethylamine, deprotonation did not occur efficiently. Removal of the acidic proton, without compromising the tetrazole ring, was achieved only by using 1,8-diazabicycloundec-7-ene (DBU) as a base (Figure S16). Reaction of the **Ir-dimer** with previously deprotonated ligand **L1** gave complex **B** in approximately 28% yield, after recrystallization with acetone and diethyl ether (Scheme 1). The ^1^H NMR spectrum showed no singlet attributable to the tetrazole CH proton, confirming the cyclometalation. Furthermore, the signals associated with the two cyclometalated ligands are nonequivalent, consistent with the nonsymmetric structure of the resulting complex (Figures S17–S19).

Concerning complex **C**, a one-pot procedure was developed by reacting **L2** with **Ir-dimer** [Ir­(ppy)_2_(μ-Cl)]_2_ in dichloromethane in the presence of Ag_2_O. The corresponding silver­(I) tetrazolylidenes were generated in situ, followed by silver–iridium transmetalation to afford the iridium complex **C** in about 32% yield (Scheme 1). During this synthesis, a coproduct was also formed, further complicating the purification of complex **C** (Figure S20–S23). However, it was possible to isolate the two compounds by column chromatography, and they were subsequently characterized by NMR and mass spectrometry. HRMS analysis confirmed the formation of compound **C**. In addition to the molecular ion peak, the mass spectrum exhibits signals attributable to the loss of one or two nitrogen atoms (Figure S29).

Therefore, the molecular structure of the unknown coproduct, complex **D**, was successfully identified (Chart 2). This iridium-based complex features two 2-phenylpyridines acting as cyclometalating ligands, along with an ancillary ligand derived from the decomposition of the tetrazole ring. This finding is consistent with the issues highlighted above, which suggest possible degradation of the tetrazole ring during the deprotonation step. The ^1^H NMR spectrum of complex **D** shows a broad signal at 9.06 ppm (H_1_ in **D**) and a doublet at 2.51 ppm (H_2_ in **D**), attributed to the methyl group (Figures S24–S26). These features are consistent with the formation of an NH group coupled to a methyl substituent. High-resolution mass spectrometry (HRMS) confirms this hypothesis, as a signal at *m*/*z* 672 was observed in positive ion mode, corresponding to (M + K)^+^ (Figure S30).

**Chart 2:**
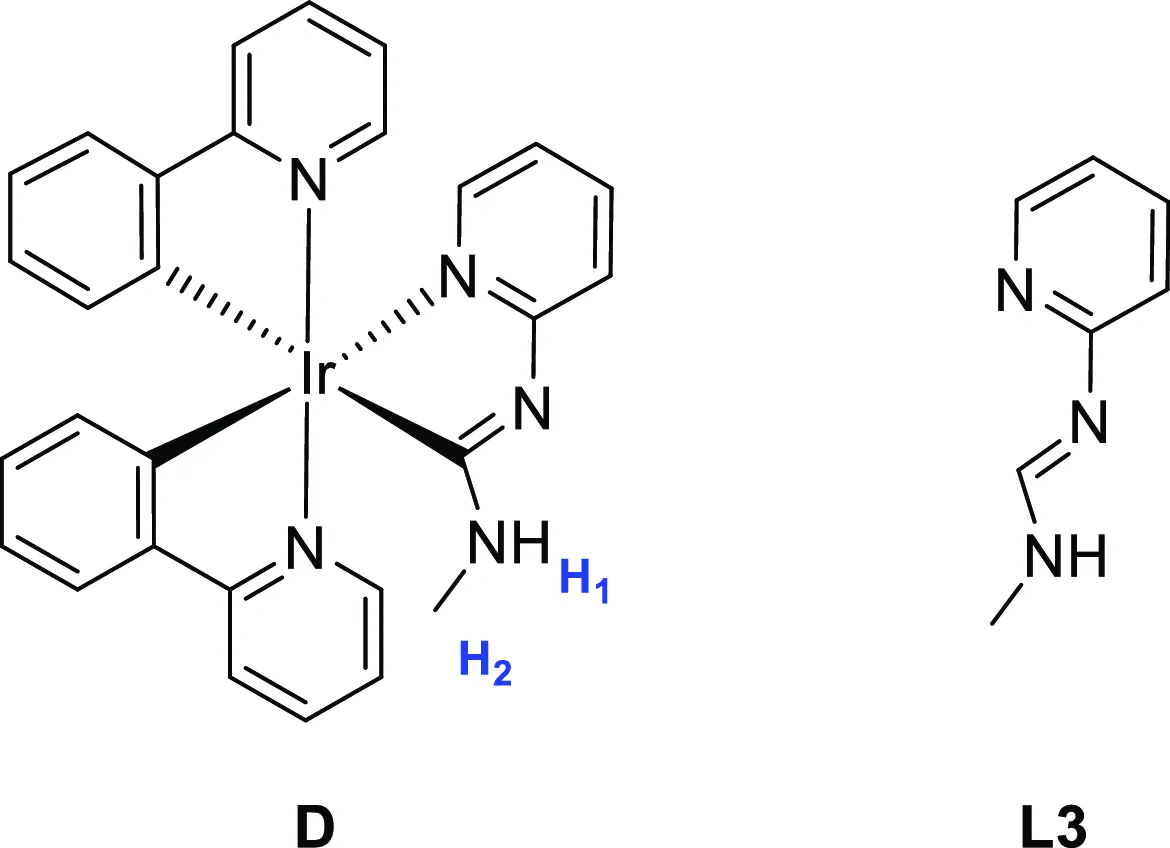
Neutral Iridium­(III) Complex D Formed as Coproduct during the Synthesis of Complex C and Degradation Product L3 Obtained from Ligand L2

### DFT Calculations: Ground-State Properties

The electronic structure of complexes **A**–**D** was investigated by DFT methods using the M06 hybrid *meta*-GGA exchange–correlation functional.^30,31^ Ground-state geometries were fully optimized without symmetry constraints in acetonitrile, through the polarizable continuum model (PCM).^35,36^ The adequacy of this computational approach for structurally analogous cyclometalated iridium­(III) complexes has been already established in previous studies.^45,46^

The resulting energy diagrams and frontier Kohn–Sham molecular orbitals are displayed in Figure 1. As is standard for this class of compounds, the HOMO of all four complexes is predominantly localized on the iridium d_π_ orbitals and on the phenyl rings of the two ppy cyclometalating ligands.^2,47^ On the contrary, the LUMO topology and energy vary substantially depending on the different nature and/or chelation mode of the tetrazolylpyridine ancillary ligand, which can also lead to both cationic (**A** and **C**) or neutral (**B** and **D**) complexes.

**Figure 1:**
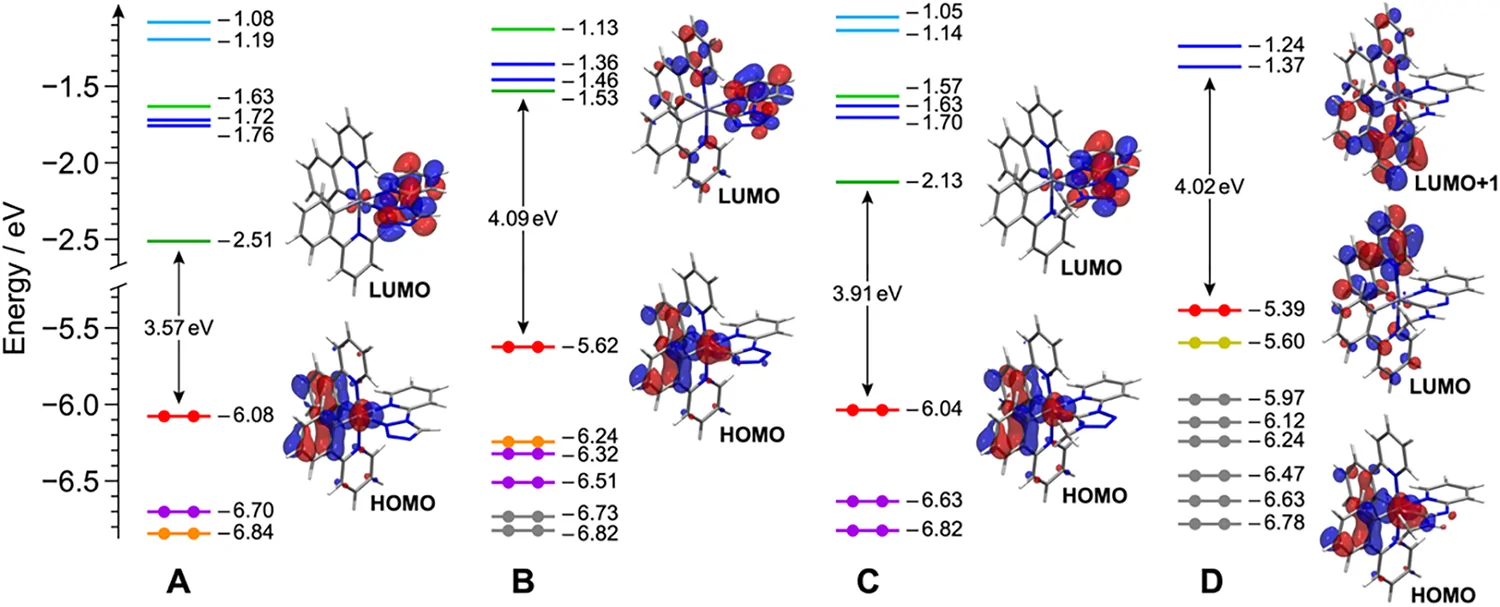
Energy diagram showing the energy values of the frontier Kohn–Sham molecular orbitals of (**A**–**D**) in acetonitrile. For some relevant orbitals, the corresponding isosurface is also displayed for the sake of clarity (isovalue = 0.04 e^1/2^ bohr^–3/2^). Along the series, relevant orbitals with similar topology are plotted with the same color for an easier comparison.

For complex **A**, the LUMO is localized on the π* system of the neutral N^∧^N **L1** ancillary ligand which, due to the presence of the tetrazole ring, features a strongly electron-deficient character. This results in a relatively low-lying LUMO and a narrow HOMO–LUMO gap (*i.e.*, 3.57 eV, Figure 1).

In complex **B**, where the deprotonated **L1** acts as a C^∧^N cyclometalating ligand, the LUMO is shifted to significantly higher energy relative to **A**. Indeed, the anionic character of the cyclometalated tetrazolide destabilizes both the HOMO and the LUMO of the complex (that is now neutral), but the effect on the LUMO (centered on the anionic ancillary ligand) is clearly more remarkable. Accordingly, the net effect on the HOMO–LUMO gap is an increase of 0.52 eV compared to **A** (*i*.*e*., 4.09 eV).

Complex **C**, bearing the tetrazolylidene carbene as an ancillary ligand, displays HOMO and LUMO energies intermediate between those of **A** and **B**. On one hand, both complexes **A** and **C** are cationic, and the σ-donation of the tetrazolylidene ancillary ligand just only slightly destabilizes the HOMO of **C** compared to that of **A**. On the other hand, the π*-accepting ability of the same tetrazolylidene ligand is lower than that of the neutral tetrazolyl–pyridine of **A**, raising the LUMO energy by 0.38 eV and widening the HOMO–LUMO gap to 3.91 eV (Figure 1).

A markedly different scenario is observed in complex **D**, due to the peculiar nature of its ancillary ligand resulting from the degradation of the tetrazolylidene carbene of complex **C**. Such a ligand, obtained upon release of molecular nitrogen, becomes an anionic cyclometalating ligand with reduced π conjugation resulting from the broken tetrazole ring. As a result: (i) the HOMO of complex **D** is the highest of the series and close to the one of **B**, since both compounds experience the very strong σ-donation of a cyclometallating anionic ancillary ligand; (ii) the LUMO of **D** is also the highest of the group and localized on the pyridine moiety of the ppy ligands, since the ancillary one does not provide low-lying π* orbitals due to broken π conjugation (Figure 1). This leads to a HOMO–LUMO gap of 4.02 eV, which is very close to the one reported for **B**.

### Electrochemistry

The electronic properties of complexes **A**–**D** were also investigated by square-wave voltammetry in room-temperature acetonitrile and dichloromethane solutions (Figures 2 and S31) and the recorded redox potentials are gathered in Table 1. All redox processes are irreversible, likely due to the lability of the nitrogen-rich ligands used across the series upon both oxidation and reduction. This experimental evidence contrasts with the fully reversible behavior typically observed for more conventional N^∧^N bipyridine-type ancillary ligands in cyclometalated iridium­(III) complexes.^2^ Moreover, complex **D** displays a remarkable instability in acetonitrile solution, so that all electrochemical data were reported in dichloromethane (Figure 2 and Table 1) and compared to acetonitrile data just in Figure S31.

**Figure 2:**
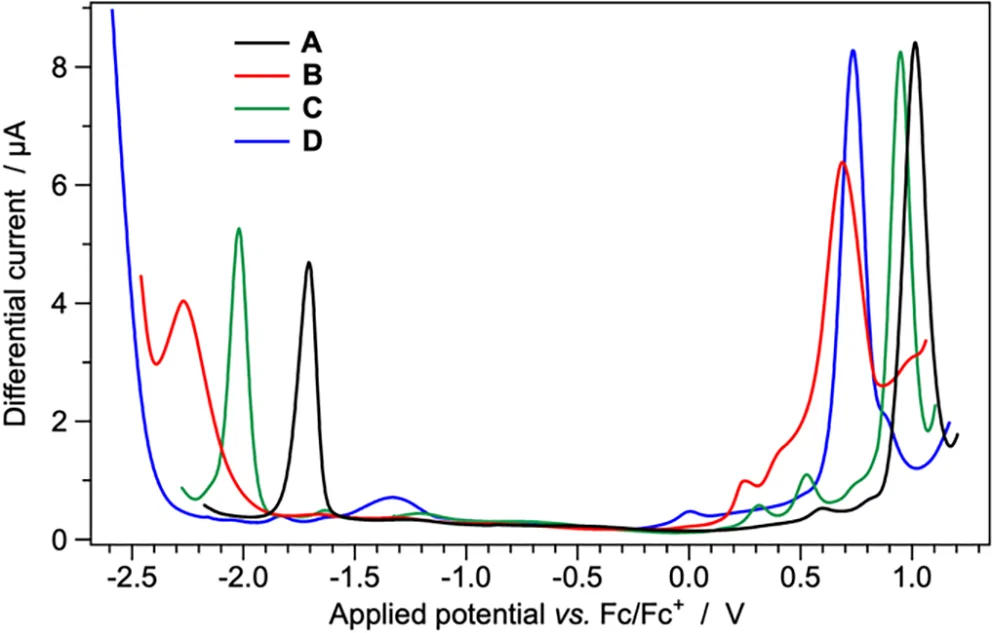
Square-wave voltammograms of complexes (**A–D**) (0.5 mM) in acetonitrile (**A–C**) and dichloromethane (**D**) solutions at 298 K.

**Table 1:** Electrochemical Data of A–D in Acetonitrile Solution (0.5 mM) + 0.1 M TBAPF6 at 298 K

	*E*_ox_ ^a^ [V]	*E*_red_ ^a^ [V]	Δ*E*_redox_ ^b^ [V]
**A**	+1.01	– 1.71	2.72
**B**	+0.69	– 2.27	2.96
**C**	+0.95	– 2.02	2.97
**D** ^c^	+0.74^c^	^c^	–

a The reported potential values are obtained by square-wave voltammetry and reported vs the ferrocene/ferrocenium couple, used as an internal reference. All of the redox processes are irreversible.

b Δ*E*_redox_ = *E*_ox_ – *E*_red_.

c Data for **D** are obtained in dichloromethane solution due to instability in acetonitrile; the reduction potential falls outside the solvent electrochemical window.

The oxidation potentials follow the order **B** < **D** < **C** < **A** (Figure 2 and Table 1), reflecting the different ligand-field strength and electron density at the iridium center imposed by the four different ancillary or cyclometalating coordination modes. Complex **B**, where the anionic tetrazolide ligand donates strongly to iridium, is oxidized most easily, while complex **A**, with the neutral N^∧^N coordination and the most electron-withdrawing **L1** ligand, exhibits the highest potential. Complexes **C** and **D** are in intermediate positions, consistent with the relatively strong σ-donation of the neutral tetrazolylidene carbene and the stronger effect exerted by the anionic cyclometalation of the ring-opened ligand, respectively.

The reduction potentials span a wider range (Table 1), reflecting the variable capability of the ligand π* system to accept electrons. Complex **A** is reduced at the least negative potential (−1.71 V), because the neutral tetrazolyl–pyridine ancillary ligand provides the lowest-lying π* orbital in the series, as confirmed by DFT calculations (Figure 1). Conversely, complex **D** displays the most negative reduction potential (<−2.5 V, outside the dichloromethane electrochemical window), since the reduction is expected to occur on the higher-lying π* orbitals on the ppy cyclometalating ligands, as suggested by DFT (Figure 1).

The resulting electrochemical redox gaps span from 2.72 V (**A**) to >3.2 V (**D**), so that complex **A** is expected to display the most red-shifted emission spectrum of the series, as also suggested by DFT calculations (see previous section).

### Photophysical Properties and Excited-State Calculations

The UV–vis absorption spectra of complexes **A**–**D** were recorded in room-temperature acetonitrile and dichloromethane solutions, and are shown in Figure 3 (the spectrum of **D** is reported just in dichloromethane solution due to incompatibility with the highly coordinative acetonitrile solvent, see Figure S32).

**Figure 3:**
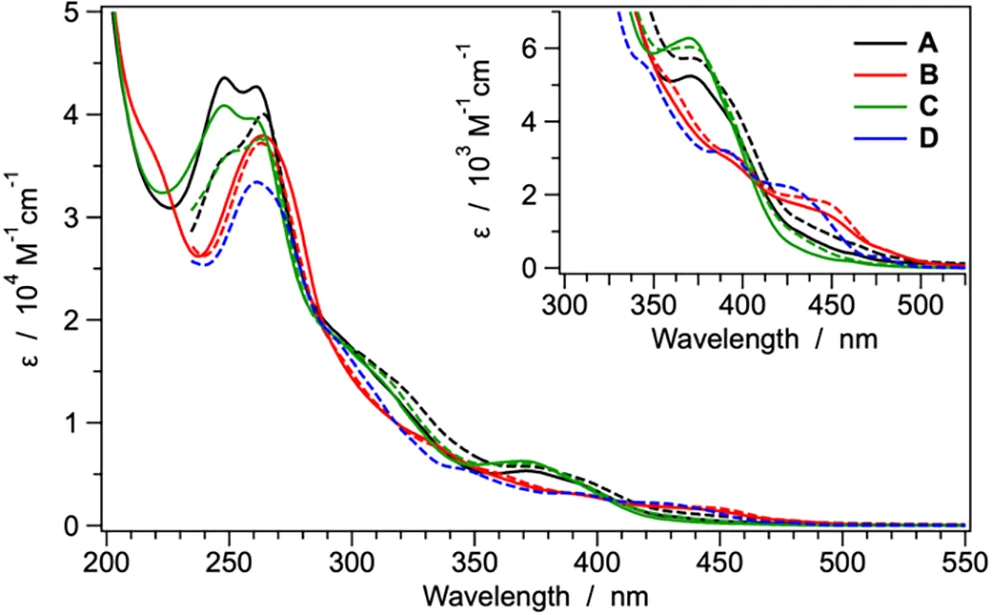
Absorption spectra of complexes **A–D** in room-temperature acetonitrile (full) and dichloromethane (dashed) solutions. Lowest-energy transitions are magnified in the inset.

All four complexes display intense absorption bands below 330 nm attributable to spin-allowed ligand-centered (LC) π–π* transitions involving both the ppy and ancillary ligands. The lower-energy region (350–500 nm) shows weaker, broader features assigned to spin-allowed metal-to-ligand charge-transfer (MLCT) transitions. The lowest-energy transitions below 450 nm are attributed to direct population of the lowest triplet state (S_0_ → T_1_) made weakly allowed by spin–orbit coupling at the iridium center.^47^

Time-dependent DFT (TD-DFT) calculations were used to provide insight into the triplet excited-state landscape of each complex at the Franck–Condon region. The lowest-lying triplet vertical excitations are collected in Tables S1–S4, expressed in terms of their dominant natural transition orbital (NTO) pairs.^39^ Figure 4 offers a compact representation of the triplet-state energy scenario at the Franck–Condon geometry across the full series.

**Figure 4:**
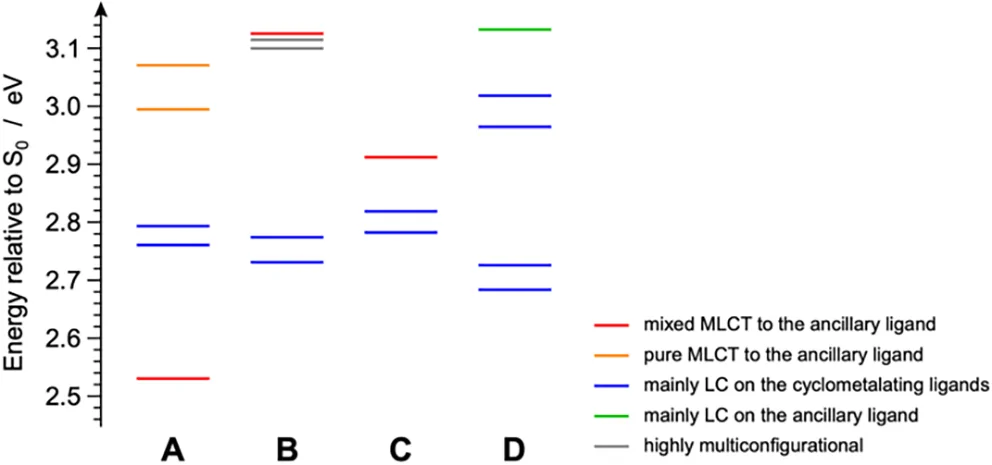
Energy diagram of the lowest-lying triplet states for complexes **A**–**D**, computed in acetonitrile as vertical excitations from the respective ground-state minimum-energy geometries.

Once again, complex **A** displays the lowest triplet vertical excitation of the series, computed at 2.53 eV above S_0_ (Table S1), which is essentially a HOMO → LUMO transition with a predominant MLCT character; T_2_ and T_3_ lie at 2.76 and 2.79 eV, respectively, and involve ppy-centered ^3^LC contributions. For the other complexes, the S_0_ → T_1_ excitation has always a predominantly ppy-based ^3^LC character and it consequently comes in pairs with the S_0_ → T_2_ transition; T_1_ is found at 2.68, 2.73, and 2.78 eV for **D**, **B**, and **C**, respectively (Tables S2–S4). Interestingly, in the cationic complex **C**, the predominant ^3^MLCT transition from the iridium center to the ancillary ligand, the same HOMO → LUMO transition observed in **A**, is estimated to correspond to the S_0_ → T_3_ excitation, calculated at 2.91 eV, due to the conversion of **L1** (as in **A**) into a tetrazolylidene carbene. Meanwhile for complex **B** the same transition is further destabilized, appearing at 3.13 eV, corresponding to S_0_ → T_5_ excitation (Figure 4).

The normalized emission spectra of all the investigated complexes are presented in Figure 5 in room-temperature acetonitrile and dichloromethane solutions (top) and in butyronitrile frozen matrix at 77 K (bottom); the associated photophysical parameters are collected in Table 2.

**Figure 5:**
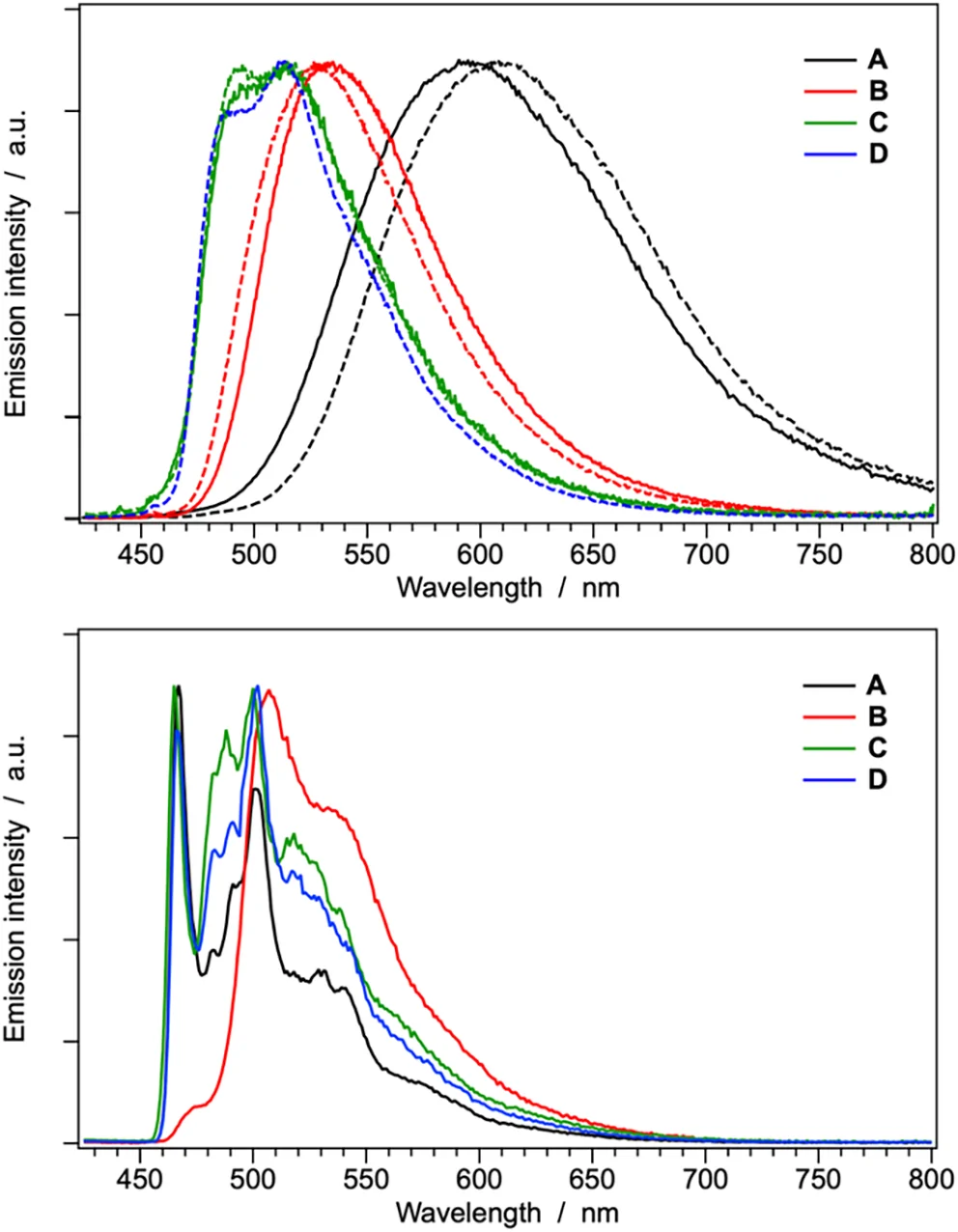
Normalized emission spectra of complexes **A–D** in acetonitrile (full) and dichloromethane (dashed) solutions at 298 K (top) and in butyronitrile glass at 77 K (bottom). Sample concentration: ≈20 μM.

**Table 2:** Luminescence Properties and Photophysical Parameters of Complexes A–D in Diluted Solutions

	CH_3_CN oxygen-free solution, 298 K					CH_2_Cl_2_ oxygen-free solution, 298 K					BuCN rigid matrix, 77 K	
	λ_em_ ^a^ [nm]	PLQY^a^ [%]	τ^b^ [μs]	*k*_r_ ^c^ [10^4^ s^–1^]	*k*_nr_ ^d^ [10^5^ s^–1^]	λ_em_ ^a^ [nm]	PLQY^a^ [%]	τ^b^ [μs]	*k*_r_ ^c^ [10^4^ s^–1^]	*k*_nr_ ^d^ [10^5^ s^–1^]	λ_em_ ^a^ [nm]	τ^b^ [μs]
**A**	592	≈ 0.5^e^	biexp.^e^	^e^	^e^	608	5.7	0.19	30	49	467, 501, 530	6.9
**B**	534	2.6	1.3	2.1	7.6	525	2.5	1.4	1.7	6.7	475^sh^, 507, 533^sh^	5.5
**C**	500^sh^, 516	0.7	1.2	0.5	8.0	497, 515	0.7	1.3	0.5	7.7	465, 500, 518	5.8
**D**	^f^	^f^	^f^	^f^	^f^	500^sh^, 514	2.3	0.86	2.7	11	467, 502, 520	6.3

a λ_exc_ = 400 nm.

b λ_exc_ = 373 nm.

c Radiative constant: *k*_r_ = PLQY/τ.

d Nonradiative constant: *k*_nr_ = 1/τ – *k*_r_.

e The complex degrades with time.

f Complex **D** decomposes rapidly in acetonitrile. ^sh^ = shoulder.

According to TD-DFT predictions (Figure 4), complex **A** shows the most red-shifted emission of the series (λ_max_ = 592 nm in acetonitrile), with a broad and featureless band profile characteristic of a ^3^MLCT transition. Surprisingly, the band shifts bathochromically to 608 nm in the less polar dichloromethane solution, opposite to the positive solvatochromism typically observed for MLCT emitters. This inverted behavior has been already documented in structurally related complexes^45^ and can be rationalized by considering that the emitting ^3^MLCT state is less polar than the ground state, as confirmed by DFT calculations of both the S_0_ and T_1_ minima of complex **A** (Figure S33). In a more polar solvent such as acetonitrile, the more polar ground state is preferentially stabilized, widening the S_0_–T_1_ gap and producing a net hypsochromic shift relative to dichloromethane.^48^

The photoluminescence quantum yield (PLQY) of **A** in acetonitrile is extremely low and associated to a multiexponential decay (Table 2), indicative of possible degradation in such a polar and coordinative solvent, probably following a mechanism analogous to that observed in **D**. Indeed, the emission spectrum of **A** in degassed acetonitrile solution varies with time, showing a progressive decrease in emission intensity (Figure S34). On the other hand, in dichloromethane complex **A** displays a 10-fold increase in the PLQY and a monoexponential lifetime (*i*.*e*., PLQY = 5.7% and τ = 0.19 μs, Table 2), despite the still high value of the nonradiative decay constant (*i*.*e*., *k*_nr_ = 4.9 · 10^6^ s^–1^, Table 2) suggests a possible deactivation through accessible ^3^MC states, as discussed in the next section.

In contrast to **A**, complexes **B**, **C**, and **D** emit at significantly higher energies, with maxima around 520 nm (Table 2). The negligible solvatochromism and the modest rigidochromic shift at 77 K (Figure 5, bottom) are both consistent with emitting states of predominantly ^3^LC character. However, the room-temperature emission profiles remain broad and poorly resolved, as typically expected for ^3^MLCT emissions, so they preclude an unambiguous assignment on purely experimental grounds.

Accordingly, spin-unrestricted DFT optimization of the lowest triplet states reveals a complex scenario, particularly for **B** and **C**, in which two ^3^LC states (each localized on one of the two nonequivalent ppy ligands) lie in close energetic proximity to the ^3^MLCT state. The adiabatic energy gaps between T_1_ and T_3_ amount to only 0.18 and 0.14 eV for **B** and **C**, respectively (Figure 6).

**Figure 6:**
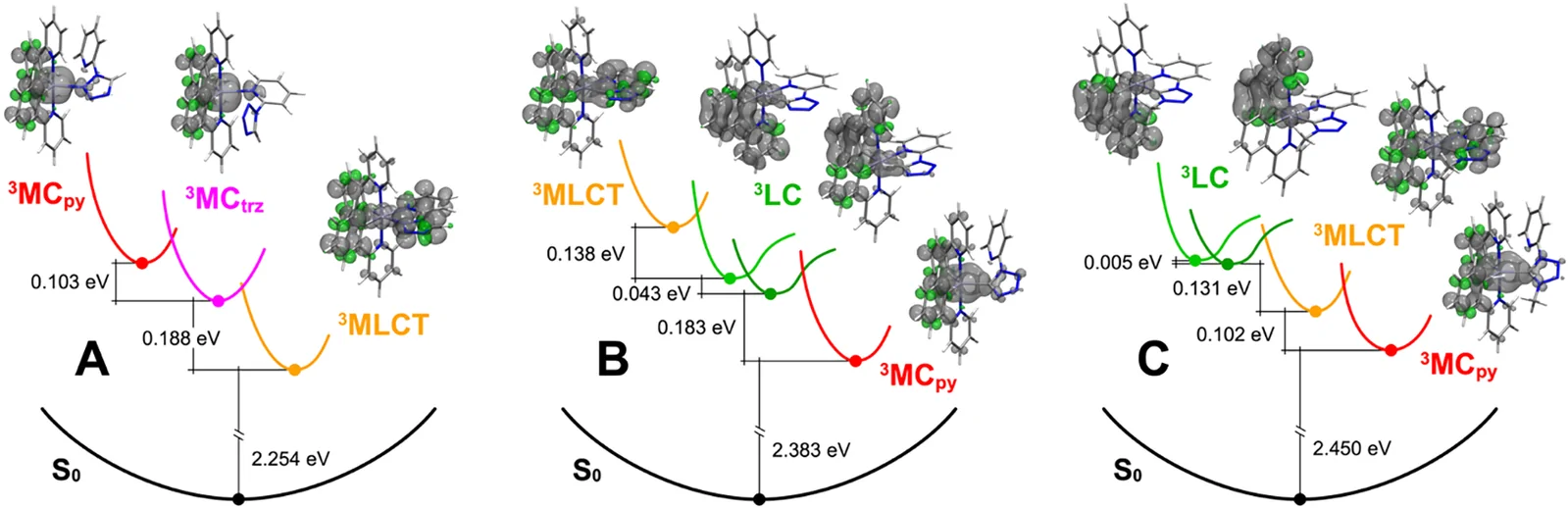
Energy diagram showing the landscape of the lowest triplets in complexes **A**, **B**, and **C**. All states have been fully optimized starting from properly selected guesses and, for all of them, the resulting relaxed minimum-energy geometry is also reported, together with its associated spin-density distribution (isovalues: 0.002 e bohr^–3^). Adiabatic energy differences between such minima are also reported. All data are computed in acetonitrile using PCM at the M06 level of theory.

In complex **B**, the two ^3^LC states constitute the lowest-lying emissive triplets, whereas in **C** the ^3^MLCT state becomes the most stable minimum upon geometry relaxation, reflecting both its already favorable energy at the Franck–Condon region (Figure 4) and the larger geometric reorganization typically associated with charge-transfer excitations (Figure 6).

On the other hand, at 77 K, the lack of both thermal equilibration and solvent reorganization (an important contribution for the stabilization of ^3^MLCT states) leads to structured and vibronically resolved emission spectra with long lifetimes for all the complexes (Figure 5, bottom and Table 2). For complexes **B**–**D**, whose lowest triplets possess predominant ppy-centered ^3^LC character (Figure 4 and Tables S2–S4), this rigidochromic behavior reflects genuine ^3^LC emission. For complex **A**, whose T_1_ presents a ^3^MLCT nature, the structured and hypsochromically shifted 77-K profile could instead be consistent with the rigidochromic behavior of the ^3^MLCT state itself. However, a rigorous ^3^MLCT/^3^LC attribution of the 77 K emission of **A** is beyond the reach of the present computational protocol, since the static PCM does not describe the frozen, nonequilibrium solvation of a low-temperature glass.

It is worth noting that PLQYs remain very low across the investigated series, suggesting an important role of nonradiative deactivation channels. It should also be emphasized that such remarkably low quantum yields, together with the limited stability of these compounds in organic solvents at low sample concentrations (Figures S31, S32, and S34), prevent a detailed discussion of their photophysical properties in solution. Accordingly, the emission properties of the complexes were also investigated in a rigid polymeric matrix, that is, dispersed in poly­(methyl methacrylate) (PMMA) at a concentration of 1% by weight. The corresponding emission spectra are reported in Figure 7, and the photophysical parameters are gathered in Table 3.

**Figure 7:**
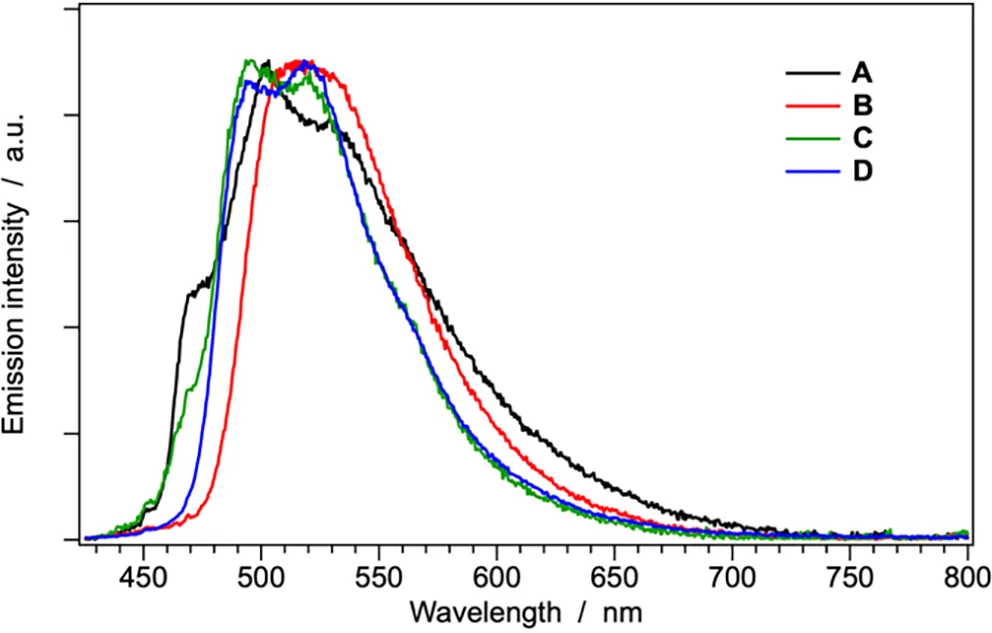
Normalized emission spectra of complexes **A**–**D** in PMMA matrix at a concentration of 1% by weight at 298 K.

**Table 3:** Luminescence Properties and Photophysical Parameters of All the Investigated Complexes in 1% PMMA Matrix at 298 K

	1% PMMA matrix, 298 K				
	λ_em_ ^a^ [nm]	PLQY^a^ [%]	τ^b^ [μs]	*k*_r_ ^c^ [10^4^ s^–1^]	*k*_nr_ ^d^ [10^5^ s^–1^]
**A**	474^sh^, 502, 530	6.7	1.7	5.8	5.4
**B**	516	9.8	1.2	8.2	7.5
**C**	495, 520	9.4	2.1	4.5	4.3
**D**	496, 520	18.8	2.0	9.6	4.2

a λ_exc_ = 400 nm.

b λ_exc_ = 373 nm.

c Radiative constant: *k*_r_ = PLQY/τ.

d Nonradiative constant: *k*_nr_ = 1/τ – *k*_r_. ^sh^ = shoulder.

A remarkable enhancement of the luminescence is observed for all the complexes once dispersed in PMMA, suggesting that the more rigid polymeric matrix is capable of limiting the important geometrical deformations of the complexes in the excited states, hence limiting the effective population of the dark ^3^MC states (see next section).

### DFT Investigation on Radiationless Pathways

The uniformly low photoluminescence quantum yields, together with the strong enhancement of emission intensity observed in frozen glass at 77 K or in polymeric matrix at 298 K, prompted a systematic investigation of nonemissive ^3^MC states by spin-unrestricted U-DFT calculations. Low-lying ^3^MC states have been identified as effective quenching channels in a range of cyclometalated iridium­(III) complexes,^2,47^ including those bearing *N*-heterocyclic carbene and triazole-based ligands,^49^ and are associated with partial or complete decoordination of an Ir–N bond,^50^ typically distorting the coordination geometry from octahedral toward trigonal bipyramidal.^51^

Two distinct families of ^3^MC states are generally distinguished in these complexes: equatorial ^3^MC states, arising from decoordination of an Ir–N bond of the ancillary ligand, and axial ^3^MC states, involving elongation of the Ir–N bonds of the cyclometalating ligands (*i.e.*, the two phenylpyridine ligands).^52,53^ In archetypal [Ir­(ppy)_2_(N^∧^N)]^+^ complexes, the former have been shown to play the more prominent role in nonradiative deactivation.^53^ In the following study, both families were considered.

For complex **A**, in which the tetrazolyl–pyridine ancillary ligand **L1** coordinates through two nitrogen atoms of comparable field strength—one from the pyridine ring (N_py_) and one from the tetrazole ring (N_trz_)—two independent ^3^MC states were considered: one involving elongation and decoordination of the Ir–N_py_ bond (resulting in the ^3^MC_py_ minimum) and one involving rupture of the Ir–N_trz_ bond (giving the ^3^MC_trz_ one). Both optimized geometries converge to a slightly distorted trigonal-bipyramidal five-coordinate structure (Figure 6). The ^3^MC_py_ state, arising from pyridine decoordination, lies 0.29 eV above the emitting ^3^MLCT triplet (T_1_), while ^3^MC_trz_, experiencing tetrazole decoordination, is closer in energy at only 0.19 eV above T_1_. Both states are therefore accessible at 298 K, with the tetrazole-decoordination pathway being more readily populated. The adiabatic energy gap between T_1_ and S_0_ is 2.25 eV, in good agreement with the experimentally found emission maximum of 592 nm (Table 2). The presence of ^3^MC-mediated nonradiative deactivation channels, accessible from the T_1_ minimum, provides a quantitative rationalization for the low PLQY of **A**. In addition to the above-mentioned ^3^MC-mediated pathways, the fast nonradiative deactivation of **A** (Table 2) is likely reinforced by direct T_1_→S_0_ intersystem crossing, favored both by the small T_1_–S_0_ energy gap and by the ^3^MLCT character of the emissive state; a quantitative assessment of this channel would require dedicated spin–orbit coupling calculations and is beyond the scope of the present work.

For complex **B**, where the ancillary pyridyl-tetrazolide coordinates the iridium center like a C^∧^N cyclometalating ligand, only a single ^3^MC geometry was optimized, corresponding to decoordination of the pyridyl nitrogen of the tetrazolide ligand. The optimized ^3^MC_py_ state lies approximately 0.20 eV below the emissive ^3^LC states (and 0.36 eV below the ^3^MLCT one), meaning such a ^3^MC minimum is found to be more stable than all the potentially emissive triplets of **B** (Figure 6). Such theoretical evidence clearly explains why complex **B** is even less emissive than complex **A** in dichloromethane, a solvent in which both complexes are relatevely stable (Table 2).

A similar scenario is also observed in the case of **C**, bearing the tetrazolylidene carbene as ancillary ligand. The ^3^MC state was obtained by rotating the pyridyl ring of the ancillary ligand to induce Ir–N decoordination (Figure 6) and the resulting minimum was estimated to stand only 0.10 eV below the emissive ^3^MLCT state, indicating that this ^3^MC state is thermally accessible from the emissive triplet at room temperature; anyway the actual efficiency of the associated nonradiative channel cannot be quantified without characterizing the transition-state barriers connecting the two minima, which is beyond the scope of the present work.

Having characterized the equatorial ^3^MC states, we also optimized the axial ^3^MC minima, involving the elongation of the Ir–N­(ppy) bonds, at the same level of theory. In all cases, these minima lie 0.56–0.58 eV above the corresponding lowest-lying equatorial ^3^MC states (Figure S35). Although a fully rigorous ranking of the two channels would require characterization of the connecting transition states rather than adiabatic energy differences alone, such a sizable separation makes a competitive contribution of the axial pathway unlikely, in line with the minor role reported for these states in related cyclometalated iridium­(III) complexes.^53^ The equatorial ^3^MC states therefore remain the dominant metal-centered quenching channel across the series.

The suppression of ^3^MC-mediated deactivation at 77 K, where thermal activation is prevented and the matrix rigidity precludes important geometrical rearrangements and ligand decoordination, accounts for the strong emission recovery in the frozen butyronitrile glass (Table 2). These computational results corroborate the experimental photophysical data and establish ^3^MC deactivations as the dominant nonradiative channels in this series of complexes.

## Conclusions

We have demonstrated that 2-(1*H*-tetrazol-1-yl)­pyridine (**L1**) is a genuinely versatile bidentate ligand scaffold that can access three different coordination modes toward the same [(ppy)_2_Ir]^+^ unit center: neutral N^∧^N coordination (complex **A**), monoanionic C^∧^N cyclometalation following base-assisted deprotonation (complex **B**), and N^∧^C: carbene coordination via its *N*-methylated derivative **L2** (complex **C**). A fourth complex (**D**), arising from partial ring-opening of the tetrazolium salt during carbene formation (with the release of molecular nitrogen), was also isolated and characterized.

Despite the limited stability and low PLQYs observed in solution—a limitation attributed to thermally accessible ^3^MC deactivation channels, but that could be somehow overcome by embedding the complexes in PMMA matrix—this work proves that a single, synthetically straightforward ligand precursor can generate three structurally and electronically distinct classes of iridium­(III)-based emitter, with full control over charge, coordination geometry, and emission character of the related complexes. The suppression of ^3^MC-mediated quenching through rational ligand design—for instance, by increasing the field strength of the pyridyl moiety of the ancillary ligand or by introducing steric constraints that raise the barrier to Ir–N decoordination—represents a clear path toward higher-performance tetrazole-based phosphors for optoelectronic applications.

## Supplementary Material





## Notes

Electronic Supporting Information (ESI) available: NMR spectra of ligands and complexes, TD-DFT vertical excitations from the ground state, other DFT-computed properties, experimental stability studies (PDF)
Cartesian coordinates of all the fully optimized geometries computed by DFT (XYZ)
