# Supplementary material for: One Scaffold, Three Chelation Modes: 2‑(1H‑Tetrazol-1-yl)pyridine as a Dynamic Ligand for Cationic and Neutral Cyclometalated Iridium(III) Complexes
Source: Inorg Chem. 2026 Aug 20;65(34):19748–59. doi: 10.1021/acs.inorgchem.6c02199 (PMC13536886; doi:10.1021/acs.inorgchem.6c02199)
Supplement: Supplementary file 1 [file ic6c02199_si_001.pdf]

Supporting Information

One Scaffold, Three Chelation Modes: 2-(1*H*-Tetrazol-1-yl)pyridine as Dynamic Ligand for Cationic and Neutral Cyclometalated Iridium(III) Complexes

Cecilia Bruschi,[¶] Dmitry Tungulin,[§] Filippo Monti,[*†] Elia Matteucci,[×]

Enrico Leoni,[‡] Letizia Sambri,[§] Nicola Armaroli,[†] and Andrea Baschieri[*†]

¶ Laboratory of Organic Electronics, Department of Science and Technology, Linköping University, 601 74 Norrköping, Sweden.

§ Department of Industrial Chemistry "Toso Montanari", University of Bologna, Via Piero Gobetti 85, 40129 Bologna, Italy.

† Institute for Organic Synthesis and Photoreactivity (ISOF), National Research Council of Italy (CNR), Via Piero Gobetti 101, 40129 Bologna, Italy.

× Department of Chemistry "Giacomo Ciamician", University of Bologna, Via Piero Gobetti 85, 40129 Bologna, Italy.

‡ Laboratorio TIMAS-MCC, ENEA, Via Ravegnana 186, 48018 Faenza (RA), Italy.

E-mail: F. M.: filippo.monti@isof.cnr.it

A. B.: andrea.baschieri@isof.cnr.it

Table of Contents	***Pages***
NMR spectra of ligands **L1** and **L2**	S2 – S5
HRMS spectrum of ligands **L1** and **L2**	S6
Characterization of **L3**	S7
NMR spectra of complex **A**	S8 – S9
NMR spectrum of ligand **L1** deprotonated	S10
NMR spectra of complexes **B** and **D**	S11 – S16
HRMS spectrum of complexes **A** - **D**	S17 – S18
Stability studies for complex **D**	S19
TD-DFT vertical excitations	S20 – S23
DFT and stability studies for complex **A**	S24
Spin-density distributions of axial ^{3}MC states	S25

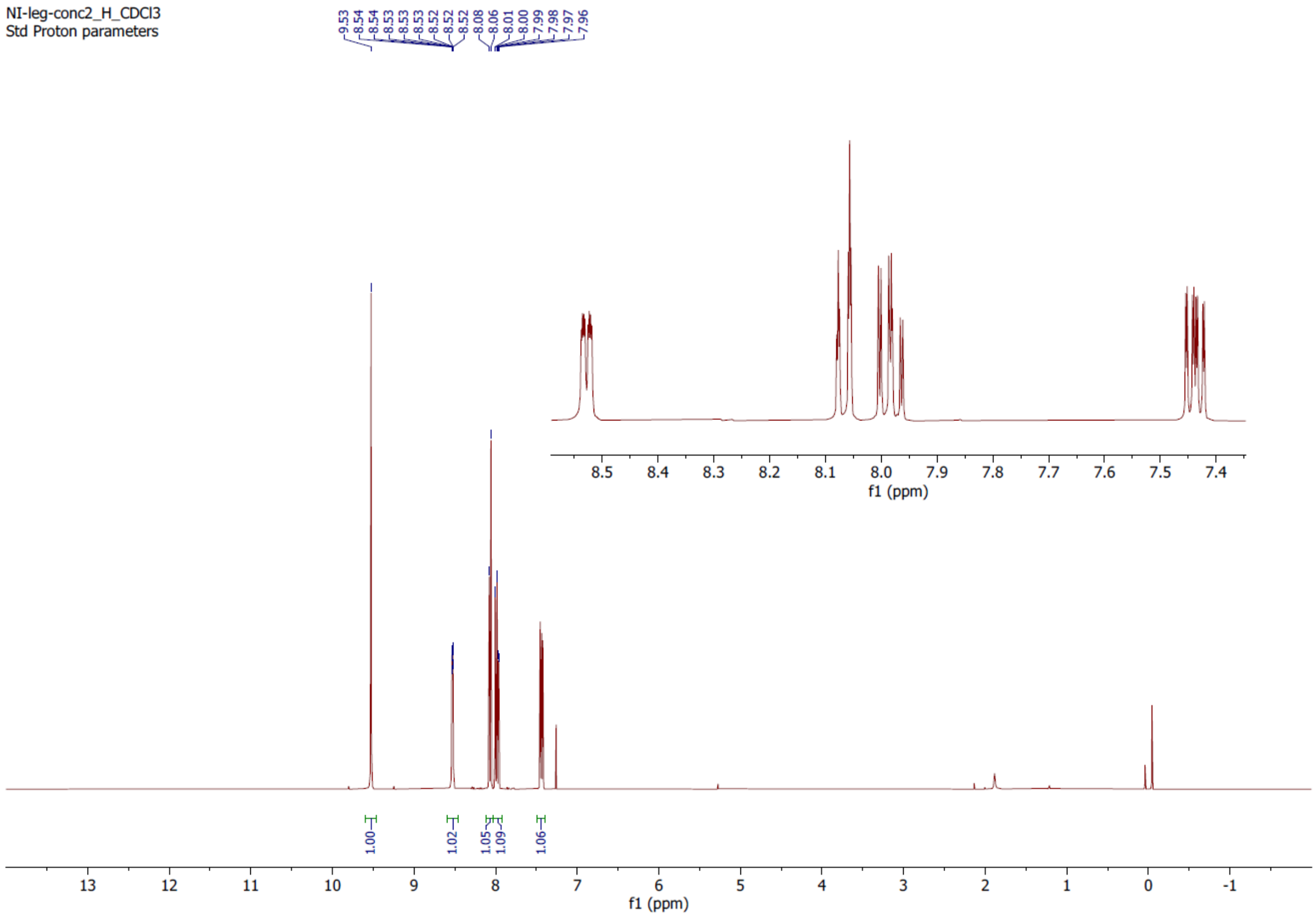

Figure S1. ^{1}H NMR spectrum of ligand **L1**.

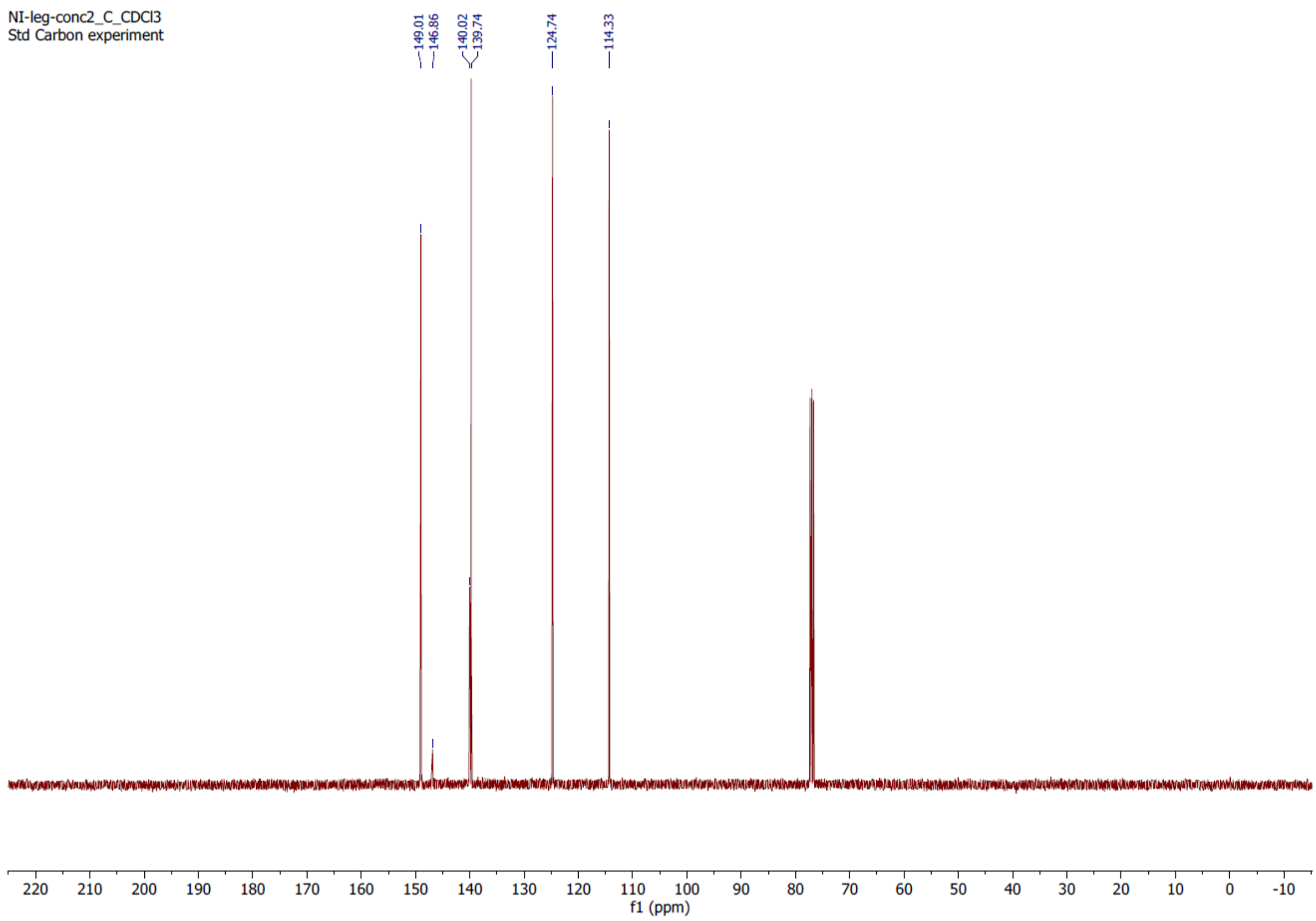

Figure S2. ^{13}C NMR spectrum of ligand **L1**.

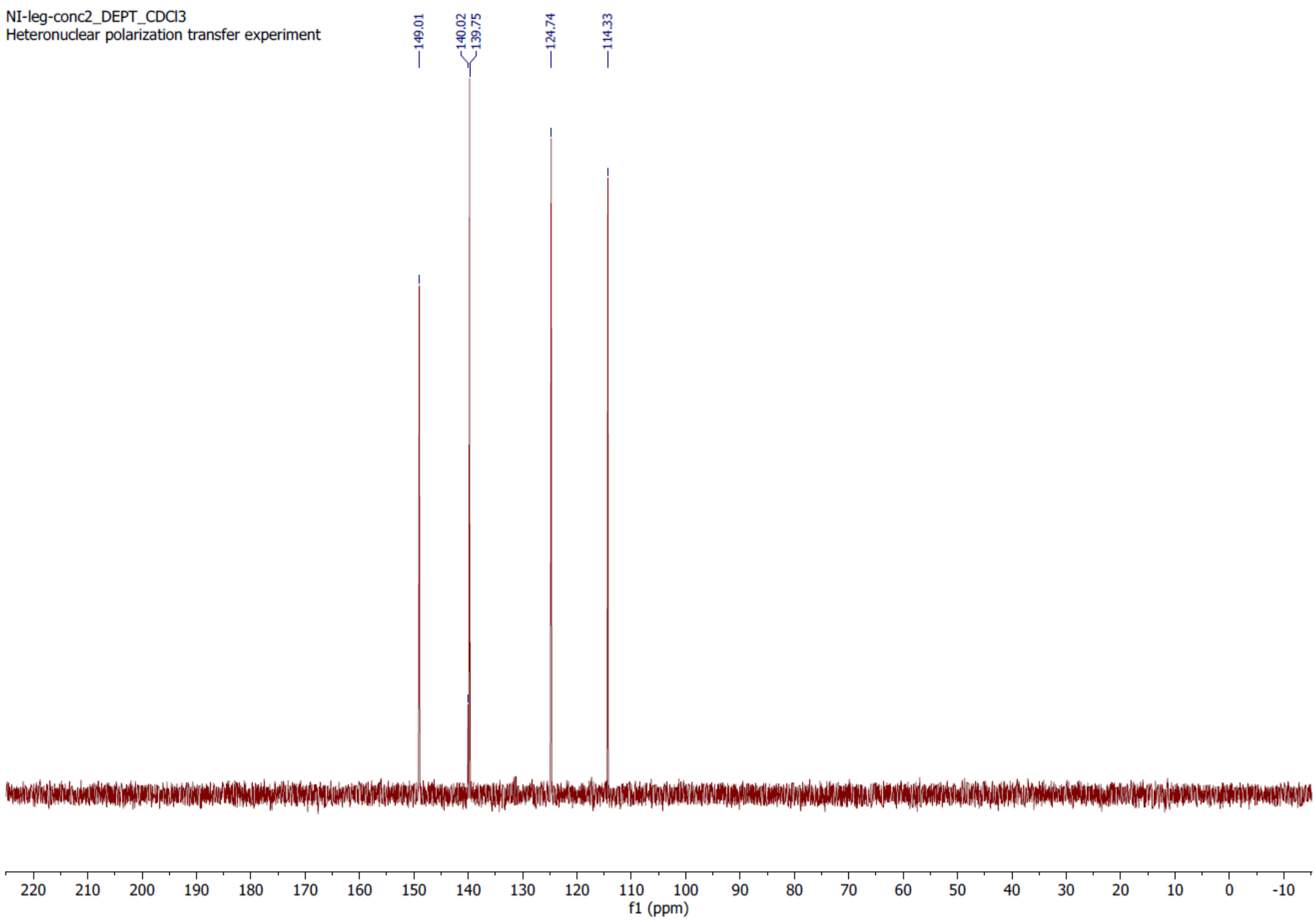

Figure S3. DEPT 135 NMR spectrum of ligand **L1**.

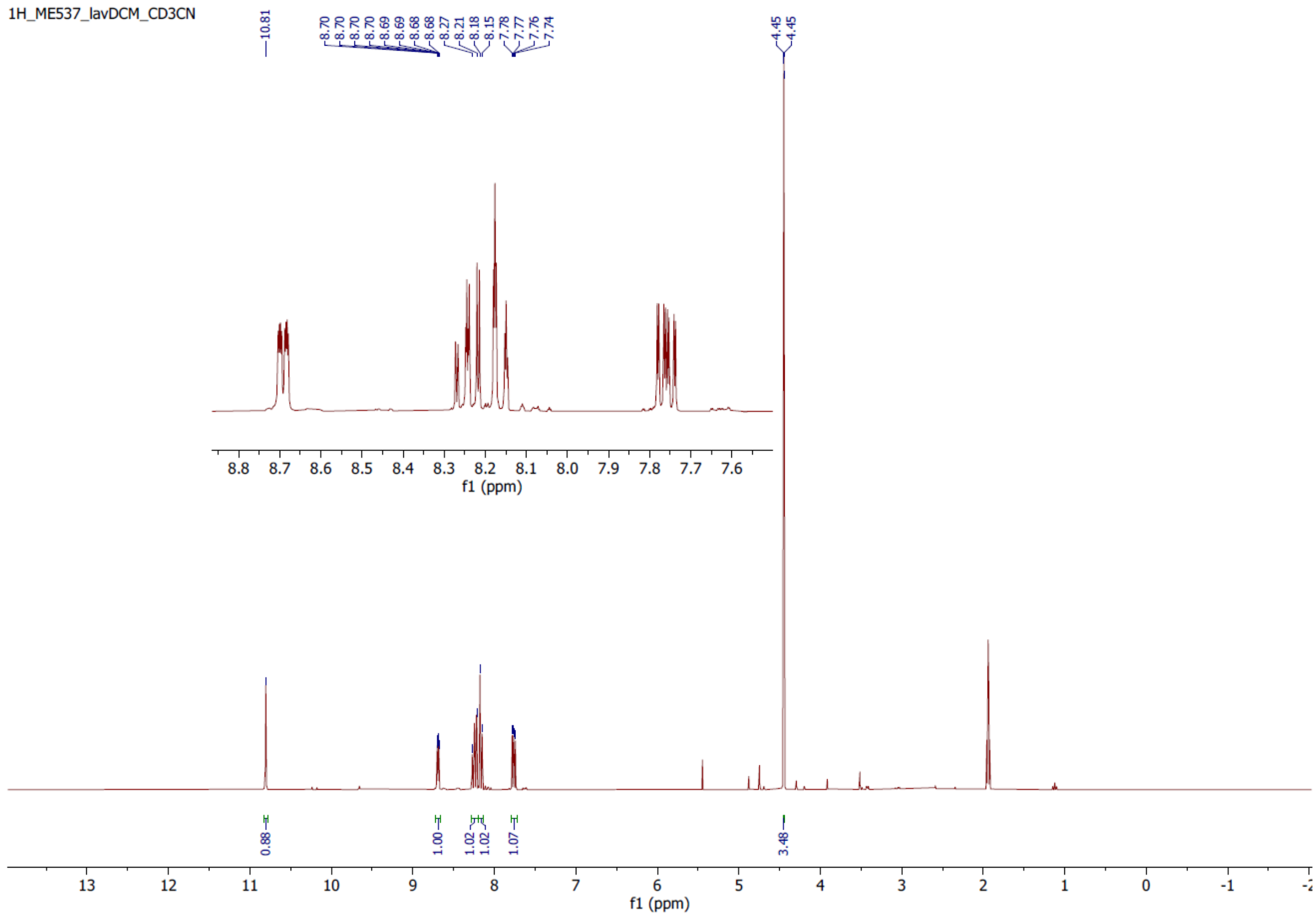

Figure S4. ^{1}H NMR spectrum of ligand **L2**.

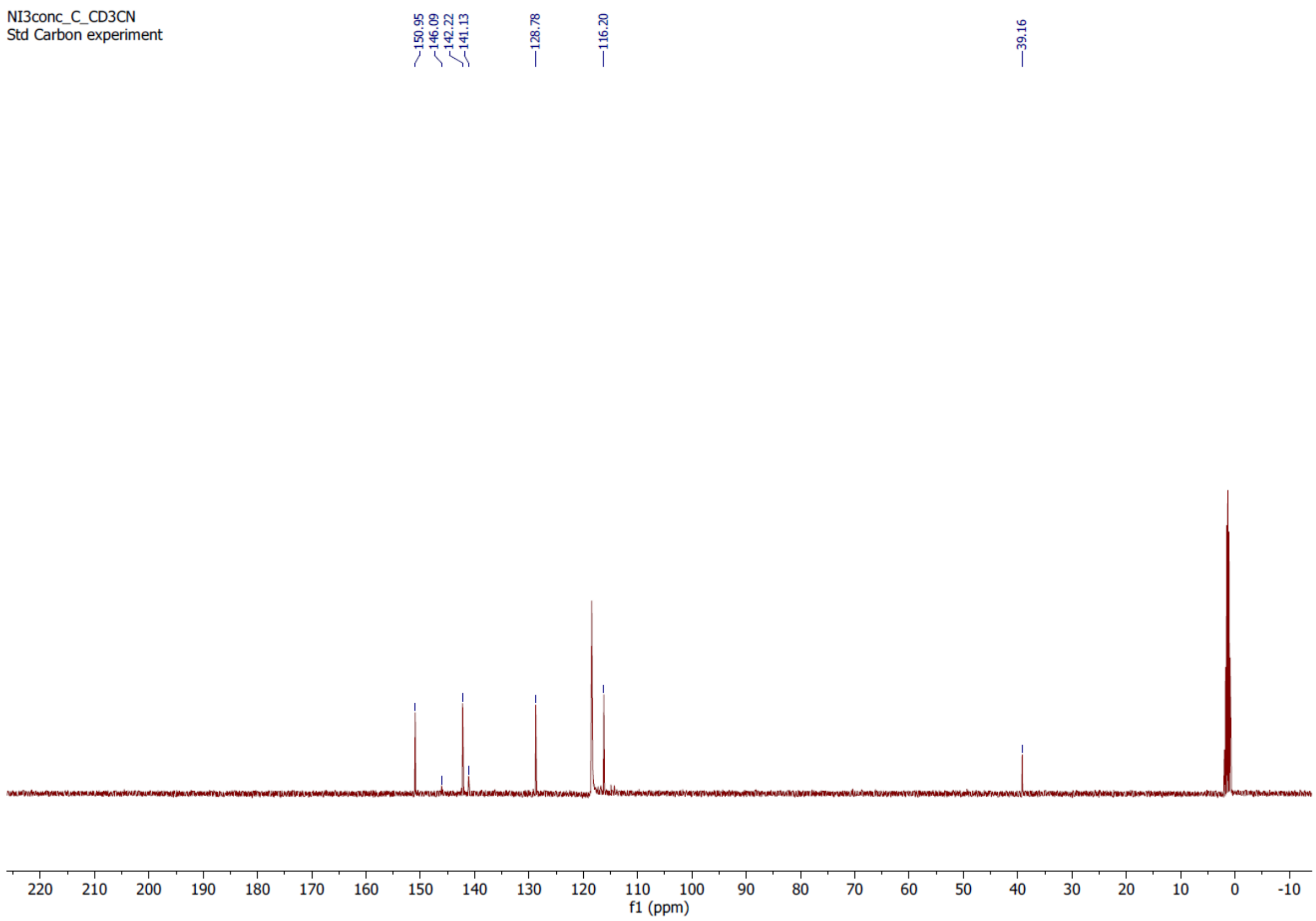

Figure S5. ^{13}C NMR spectrum of ligand **L2**.

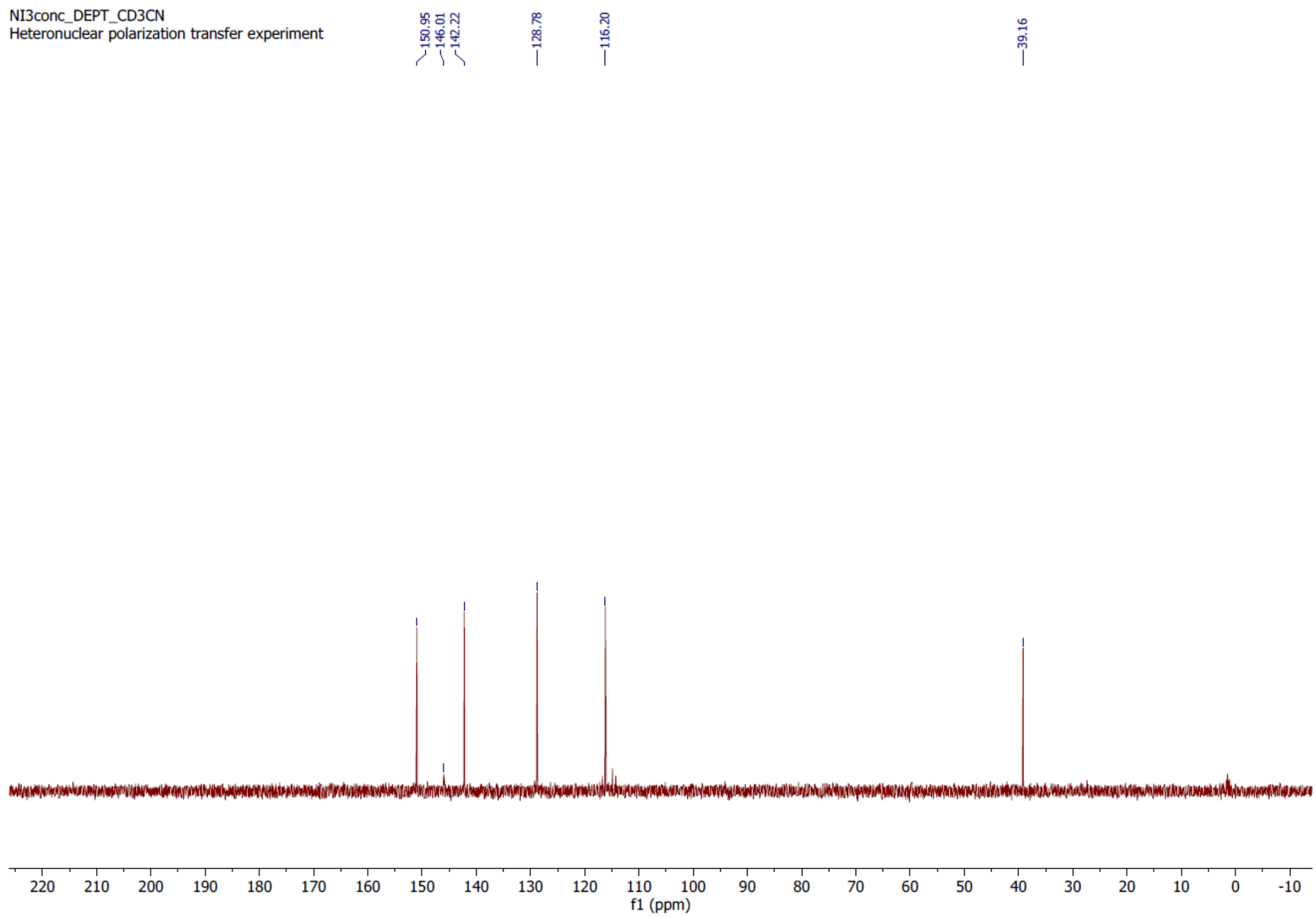

Figure S6. DEPT 135 NMR spectrum of ligand **L2**.

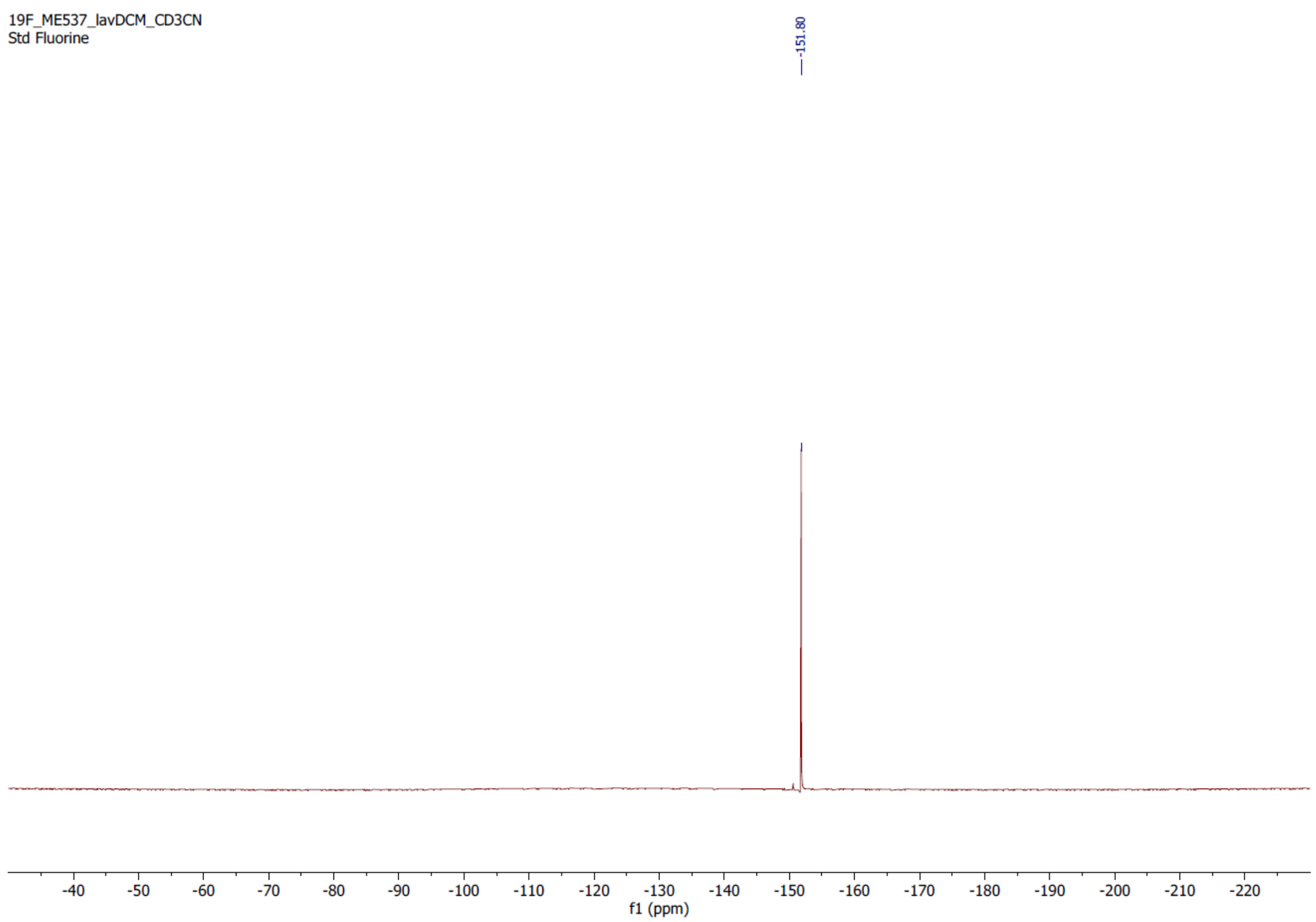

Figure S7. ^{19}F NMR spectrum of ligand **L2**.

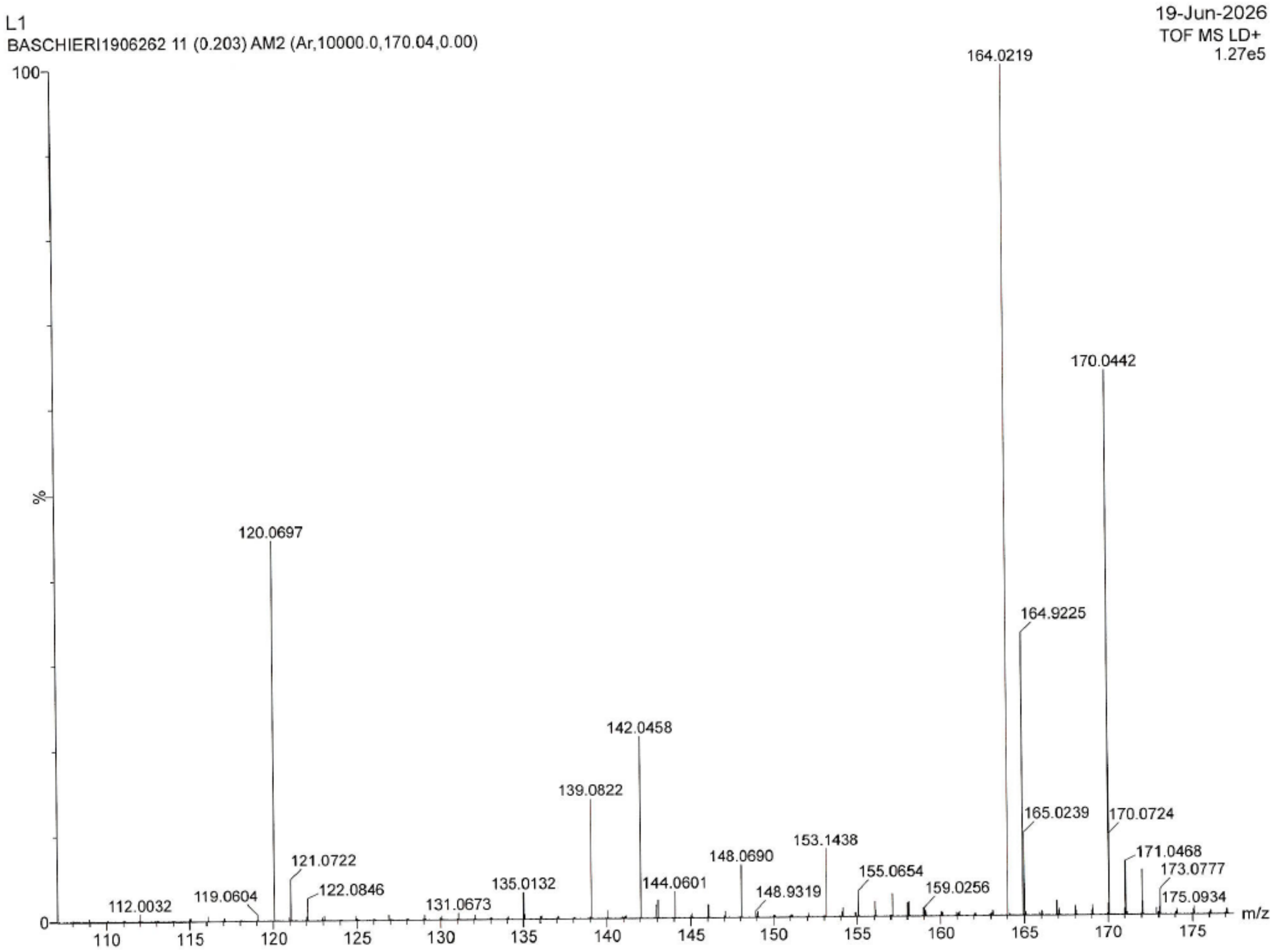

Figure S8. High-resolution mass spectrum of ligand **L1**.

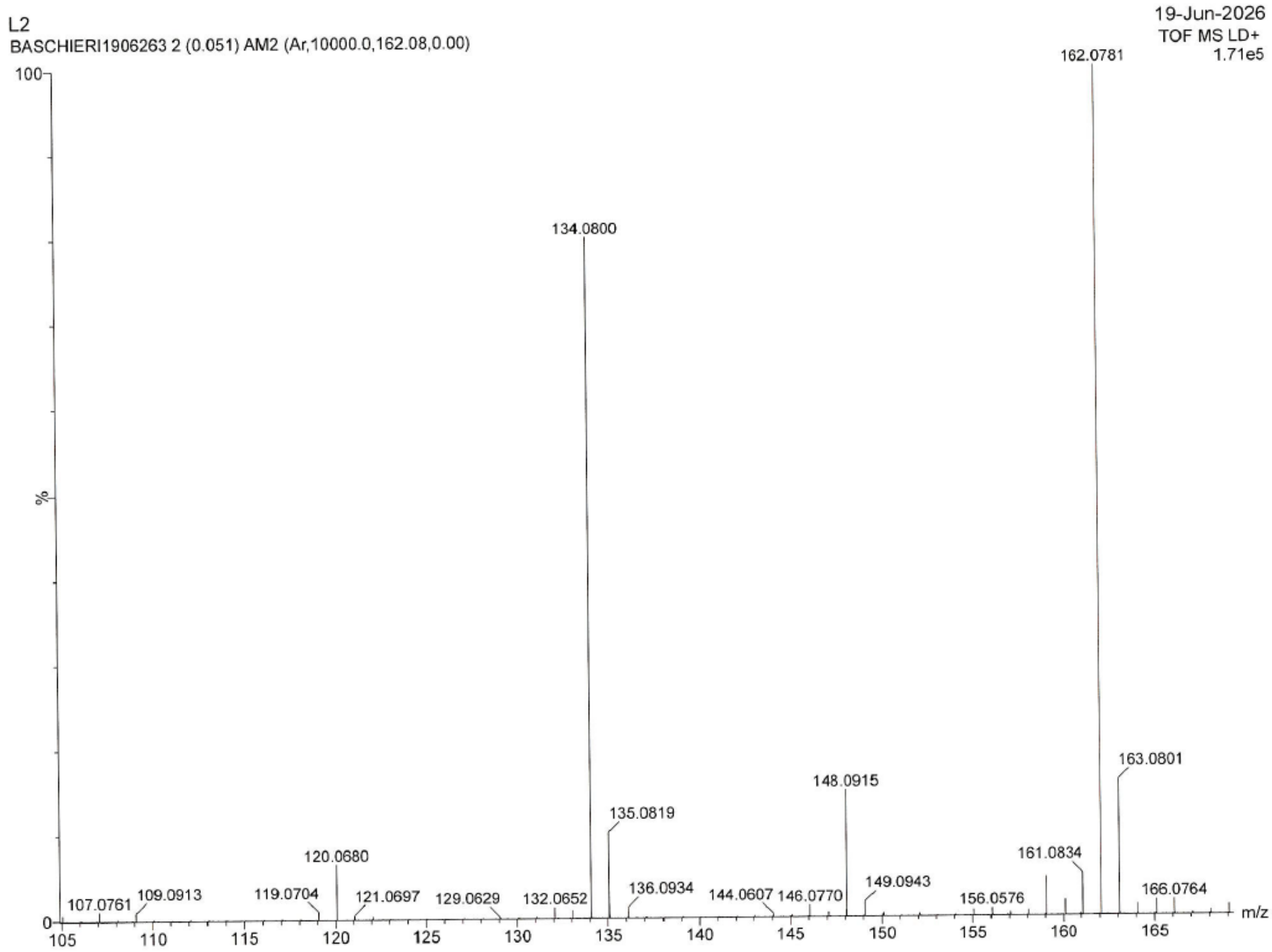

Figure S9. High-resolution mass spectrum of ligand **L2**.

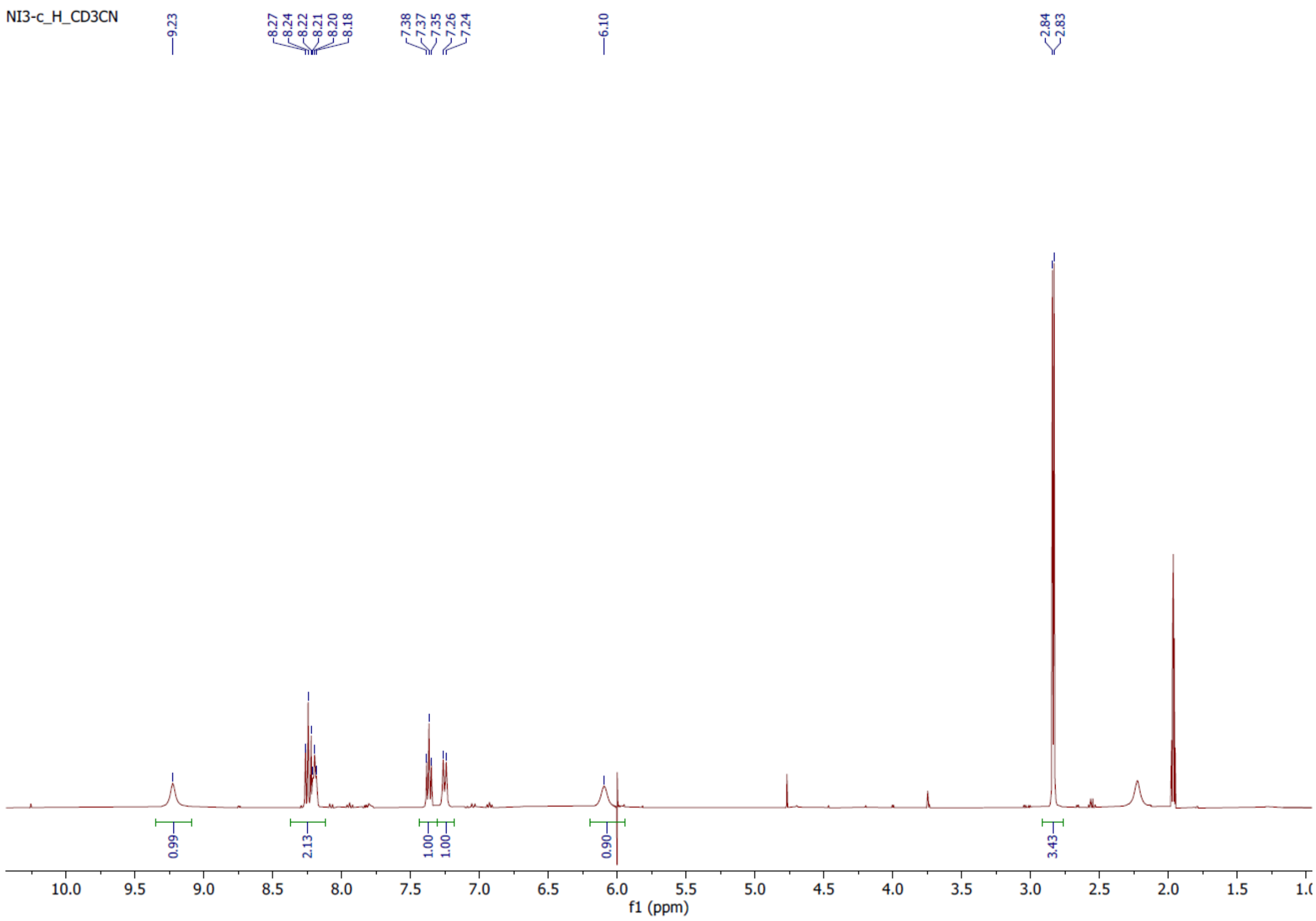

Figure S10. ^{1}H NMR spectrum of decomposed ligand **L3**.

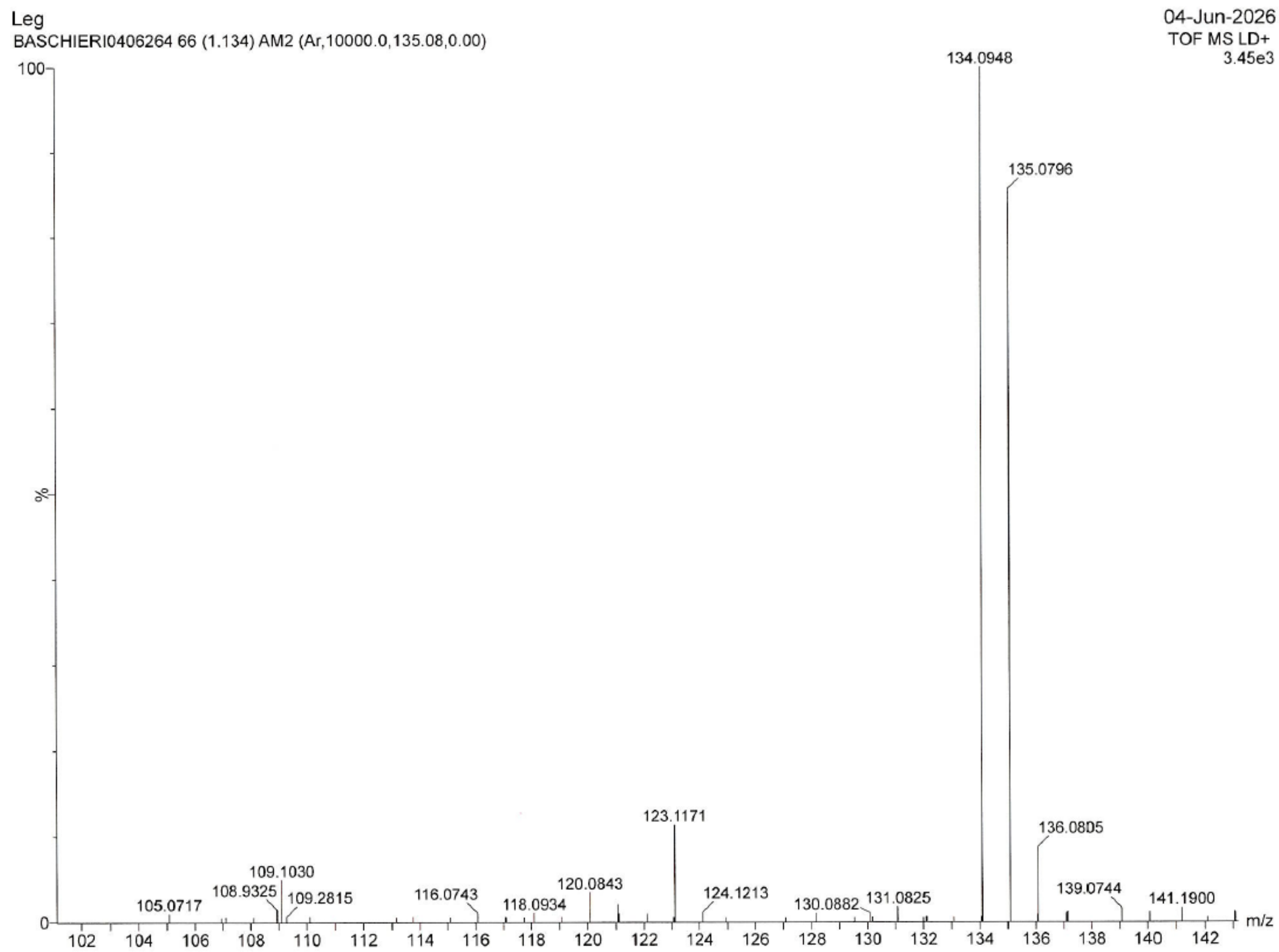

Figure S11. High-resolution mass spectrum of ligand **L3**.

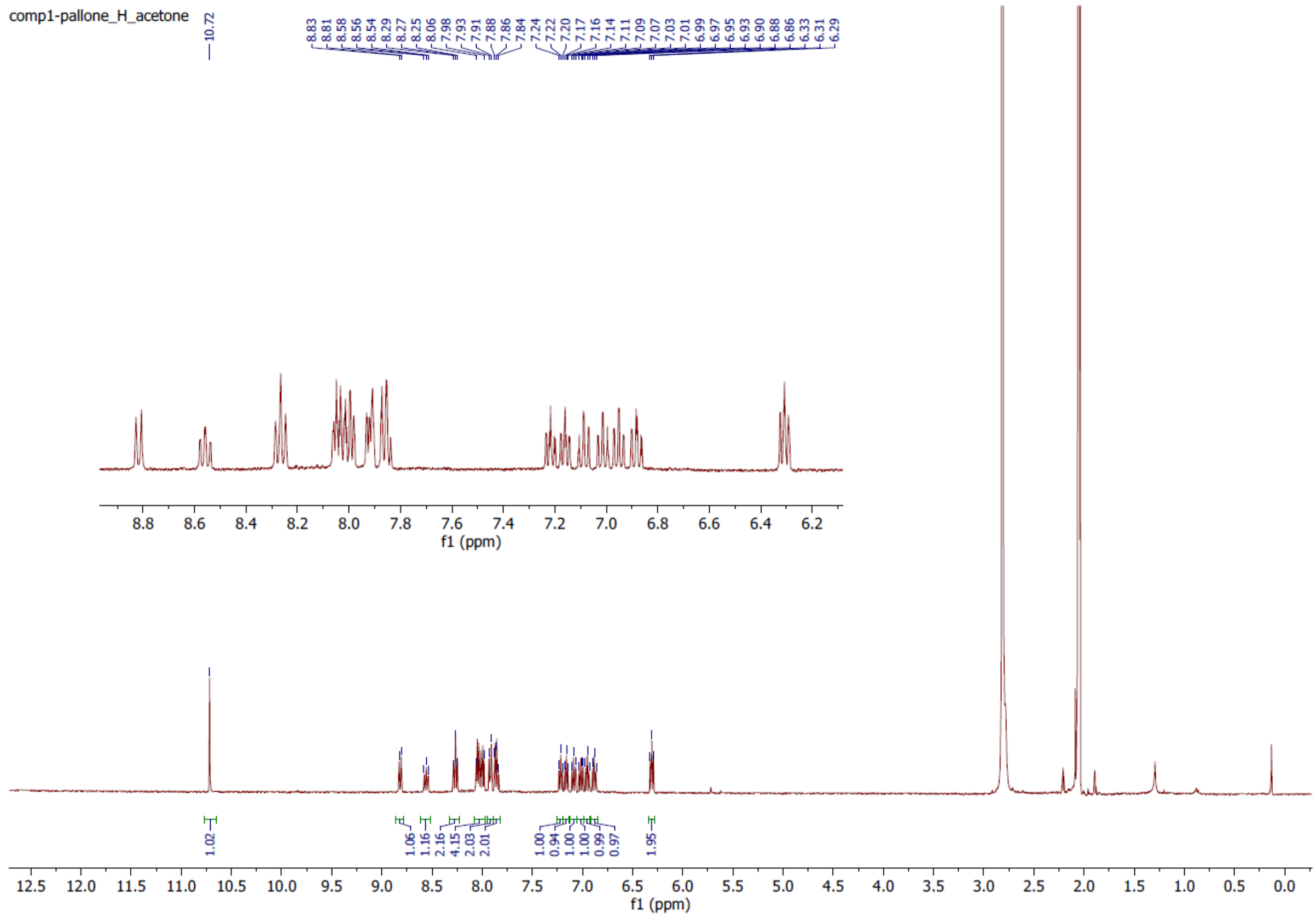

Figure S12. ^{1}H NMR spectrum of complex **A**.

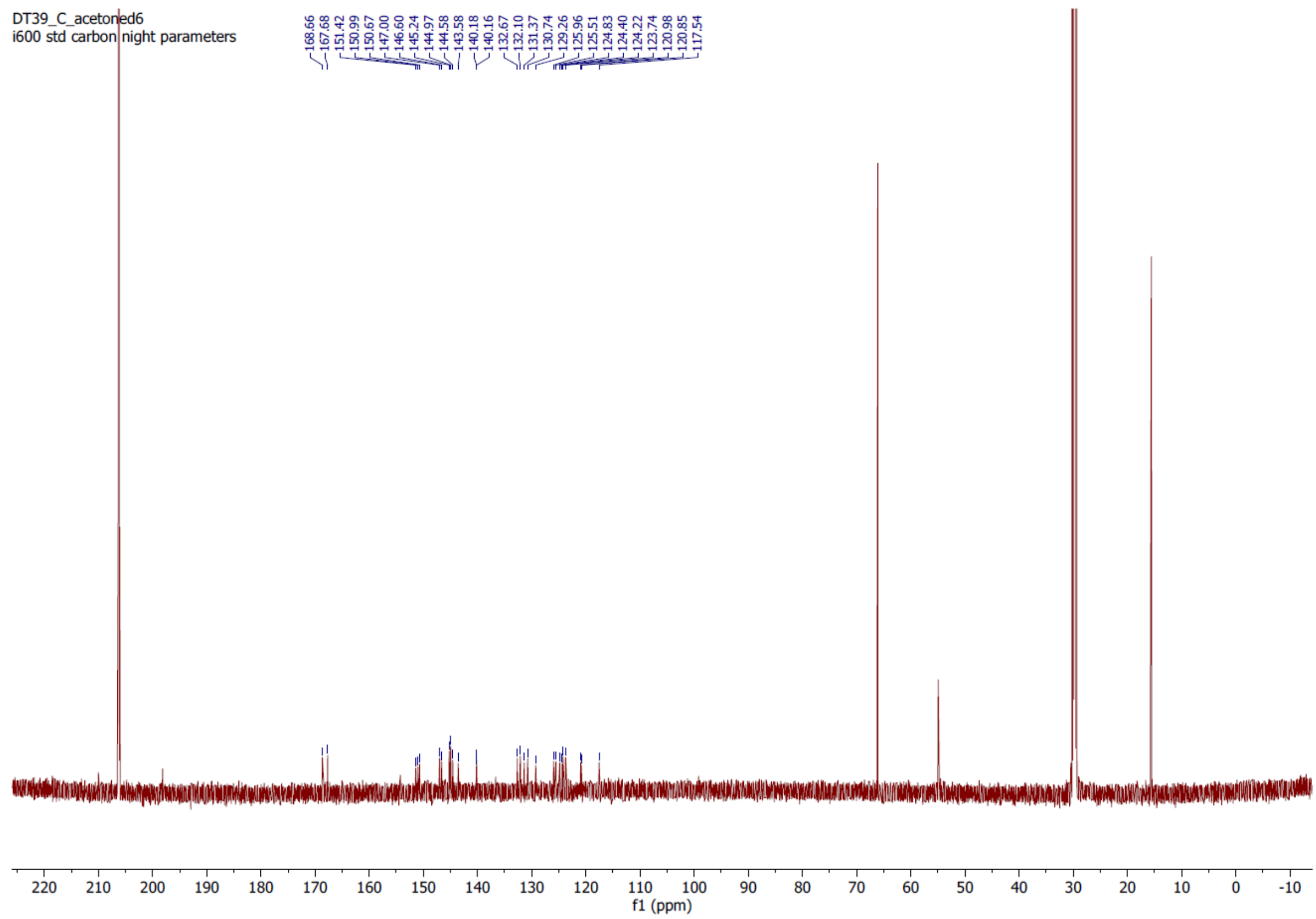

Figure S13. ^{13}C NMR spectrum of complex **A**.

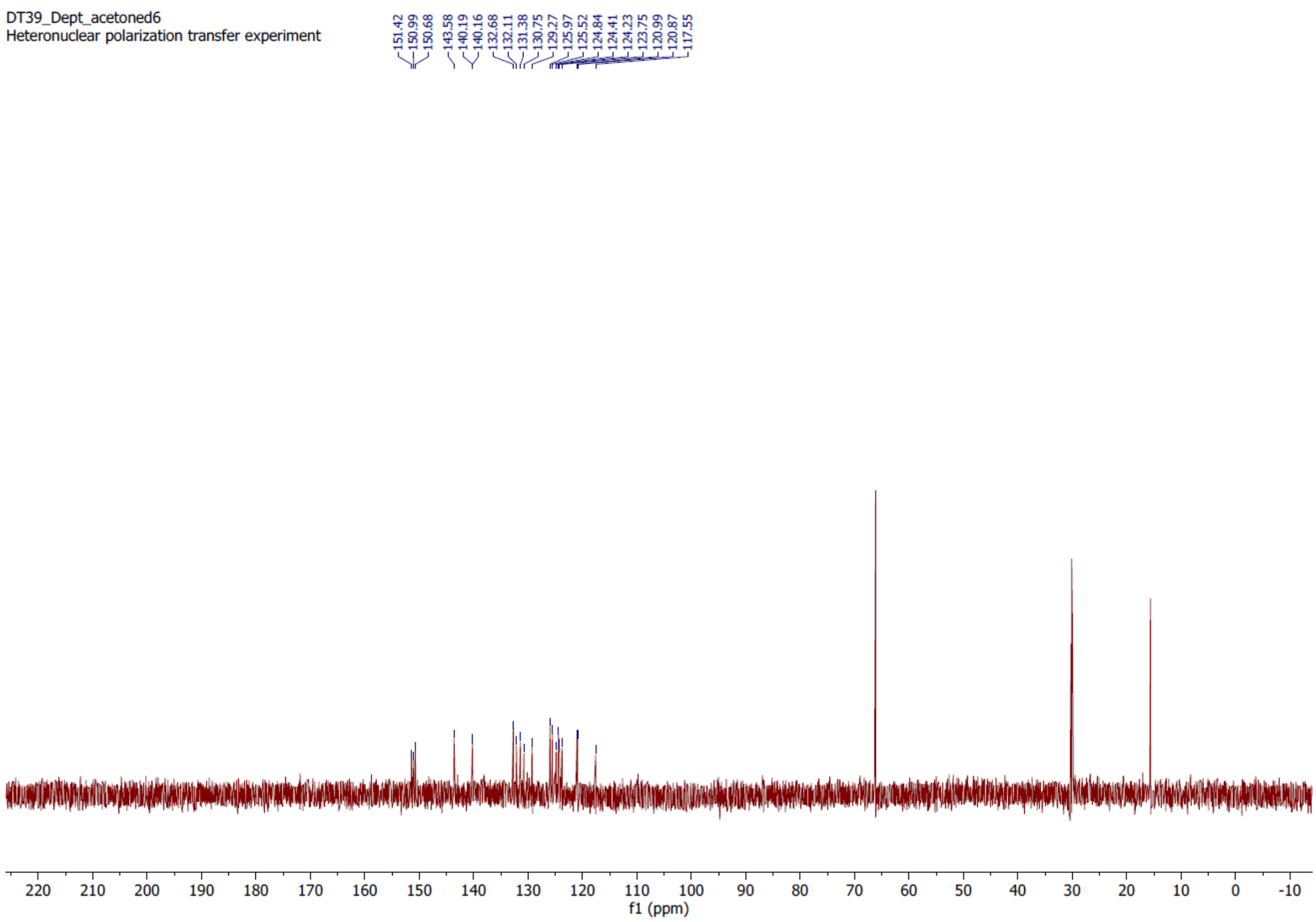

Figure S14. DEPT 135 NMR spectrum of complex **A**.

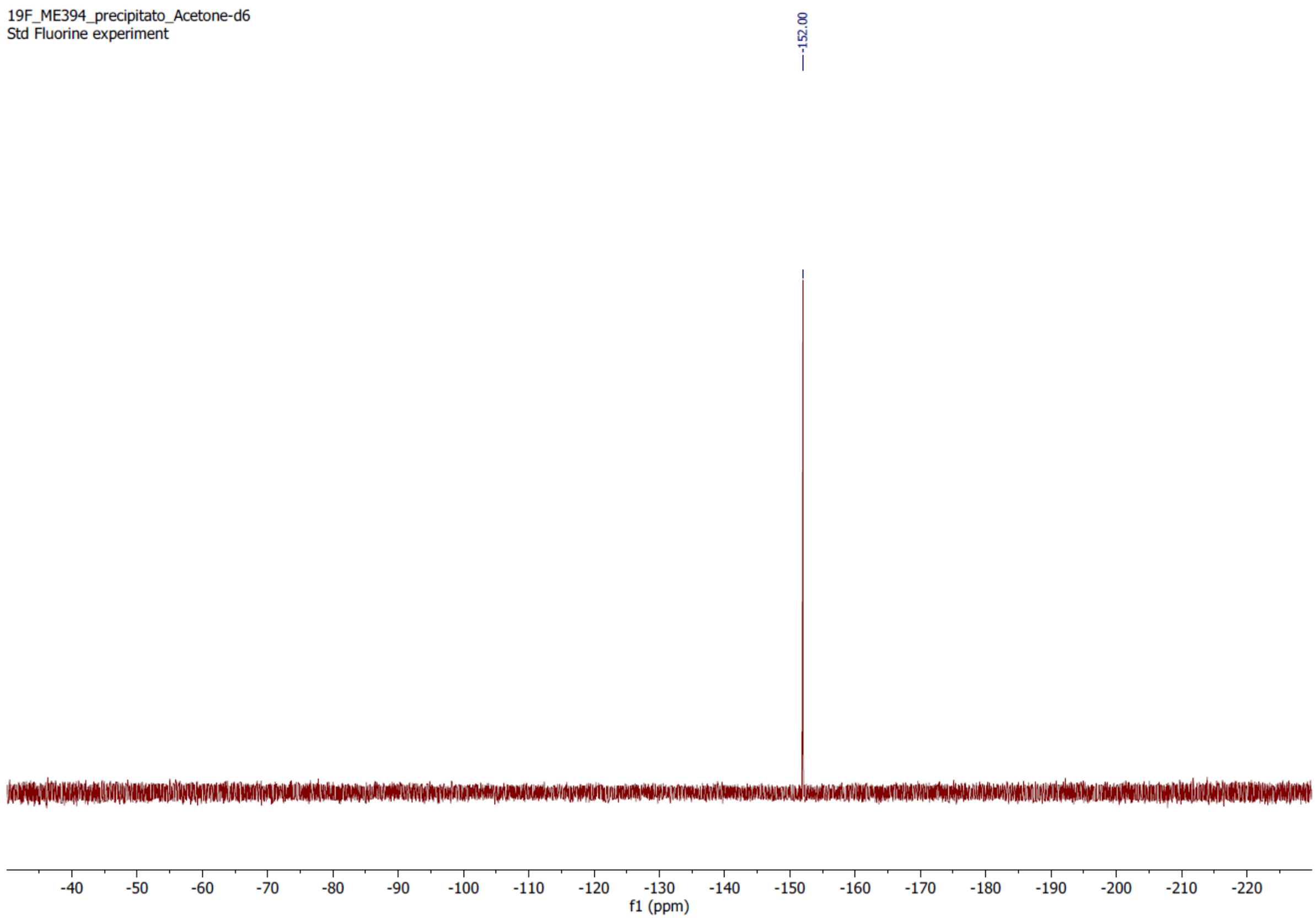

Figure S15. ^{19}F NMR spectrum of complex **A**.

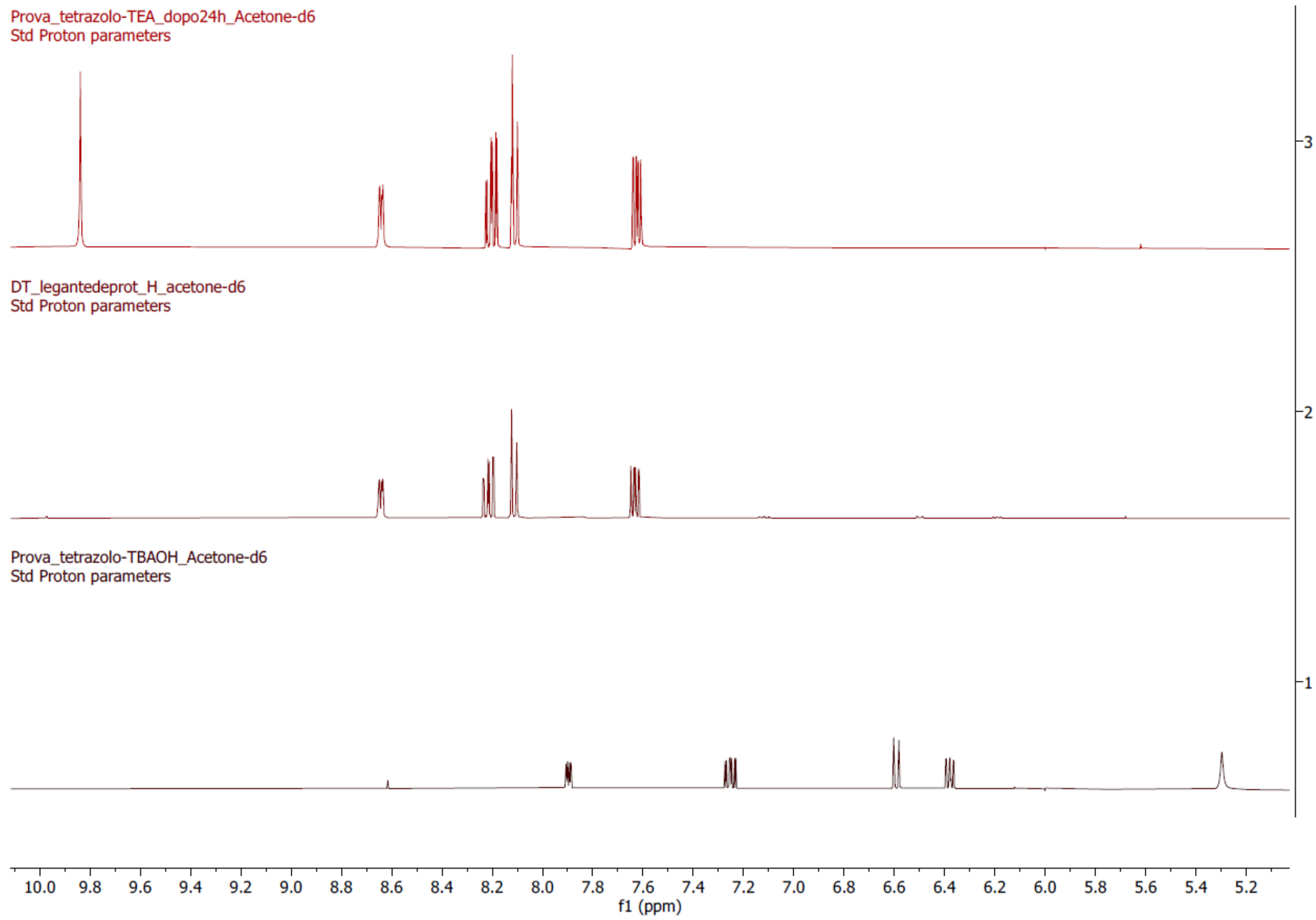

Figure S16. ^{1}H NMR spectrum shows the deprotonation effect of TEA, DBU or TBAOH on ligand **L1**.

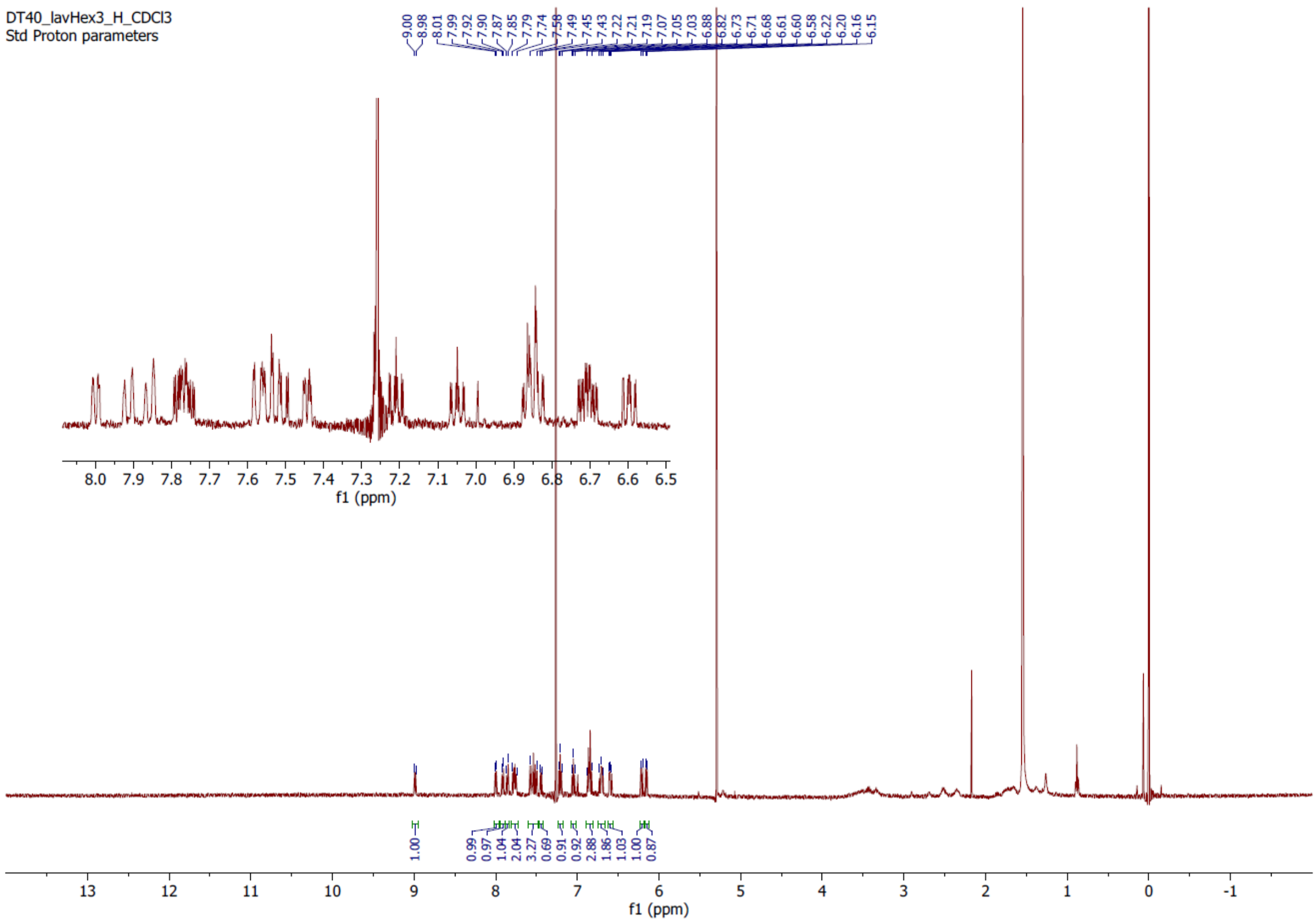

Figure S17. ^{1}H NMR spectrum of complex **B**.

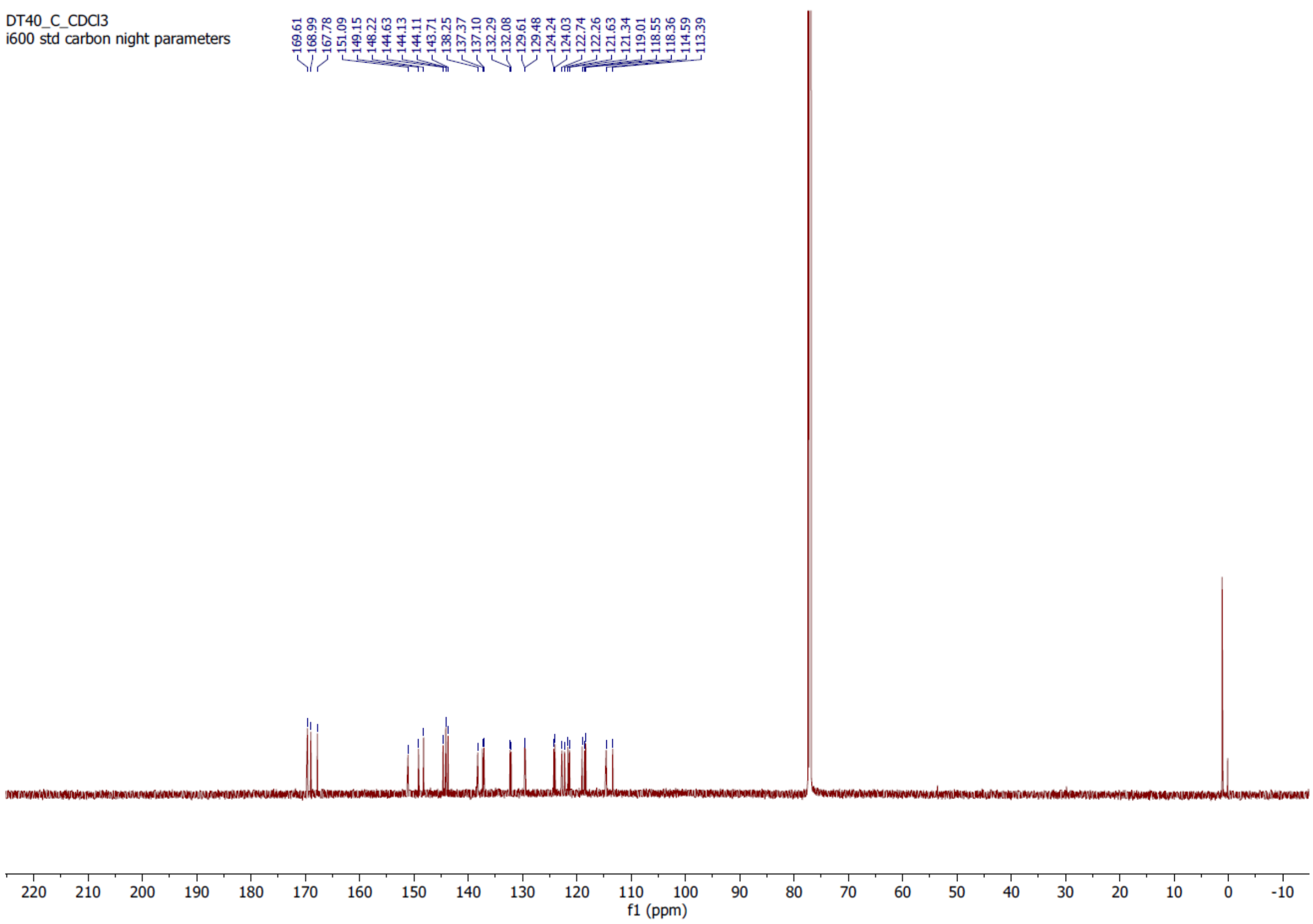

Figure S18. ^{13}C NMR spectrum of complex **B**.

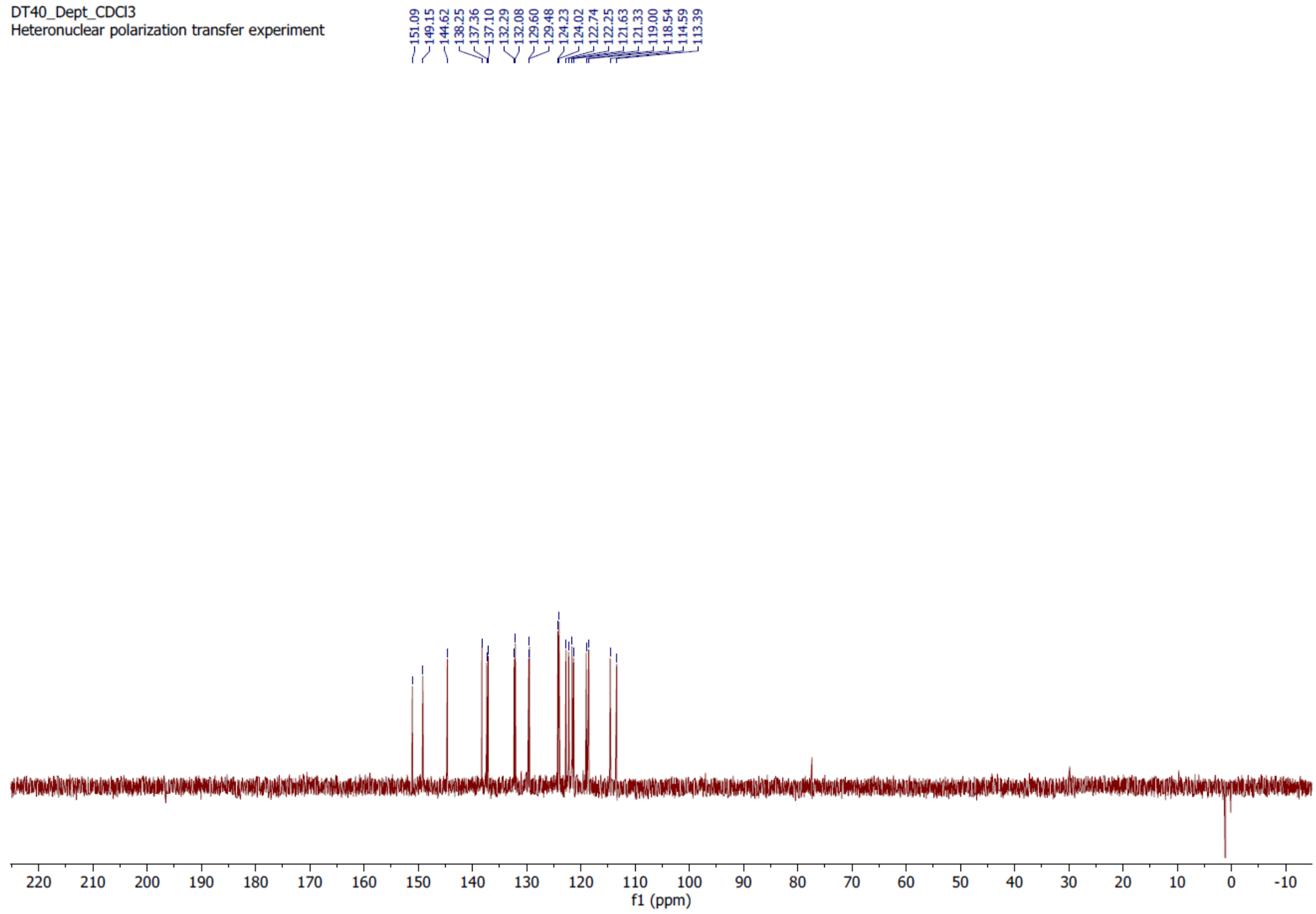

Figure S19. DEPT 135 NMR spectrum of complex **B**.

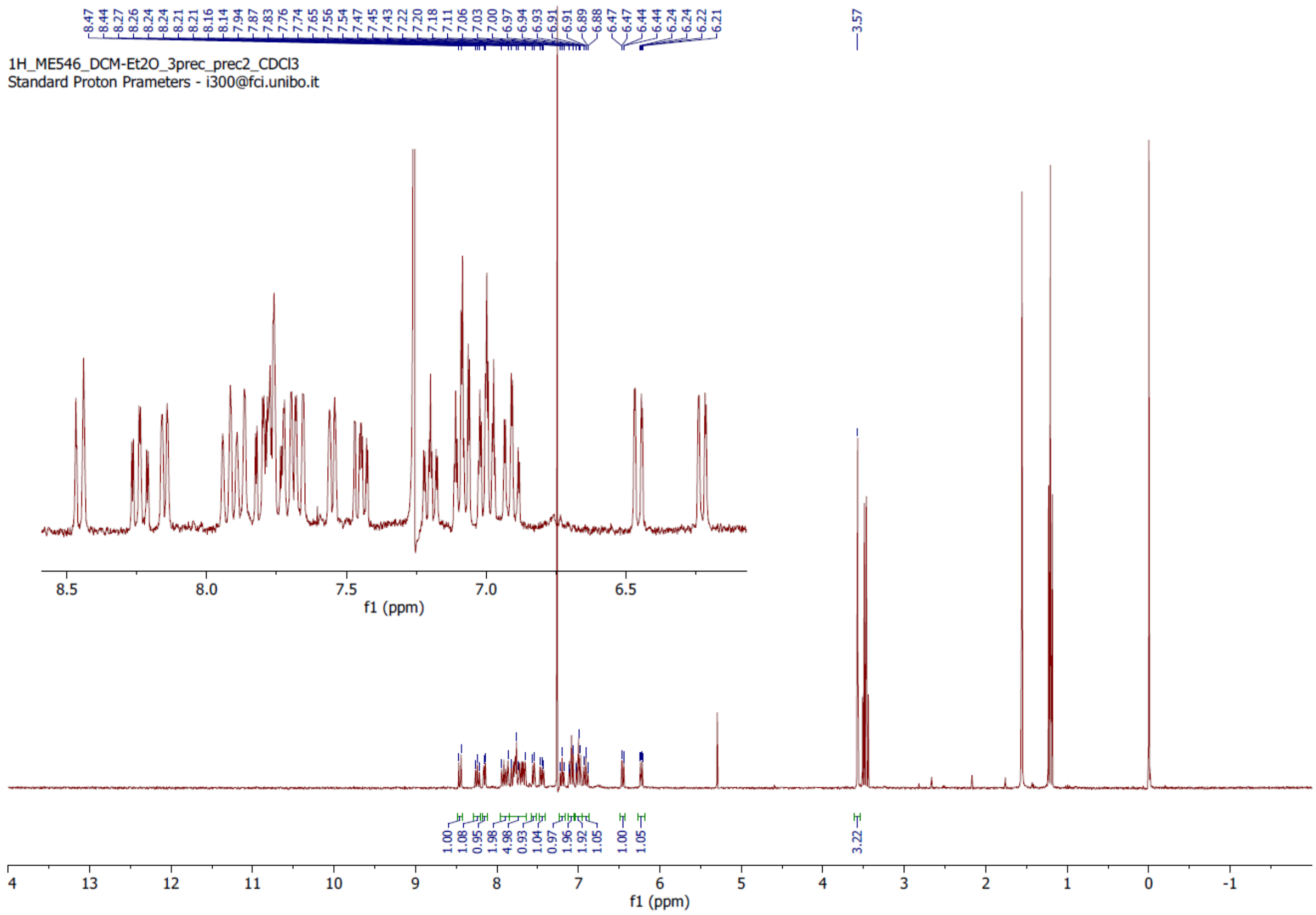

Figure S20. ^{1}H NMR spectrum of complex **C**.

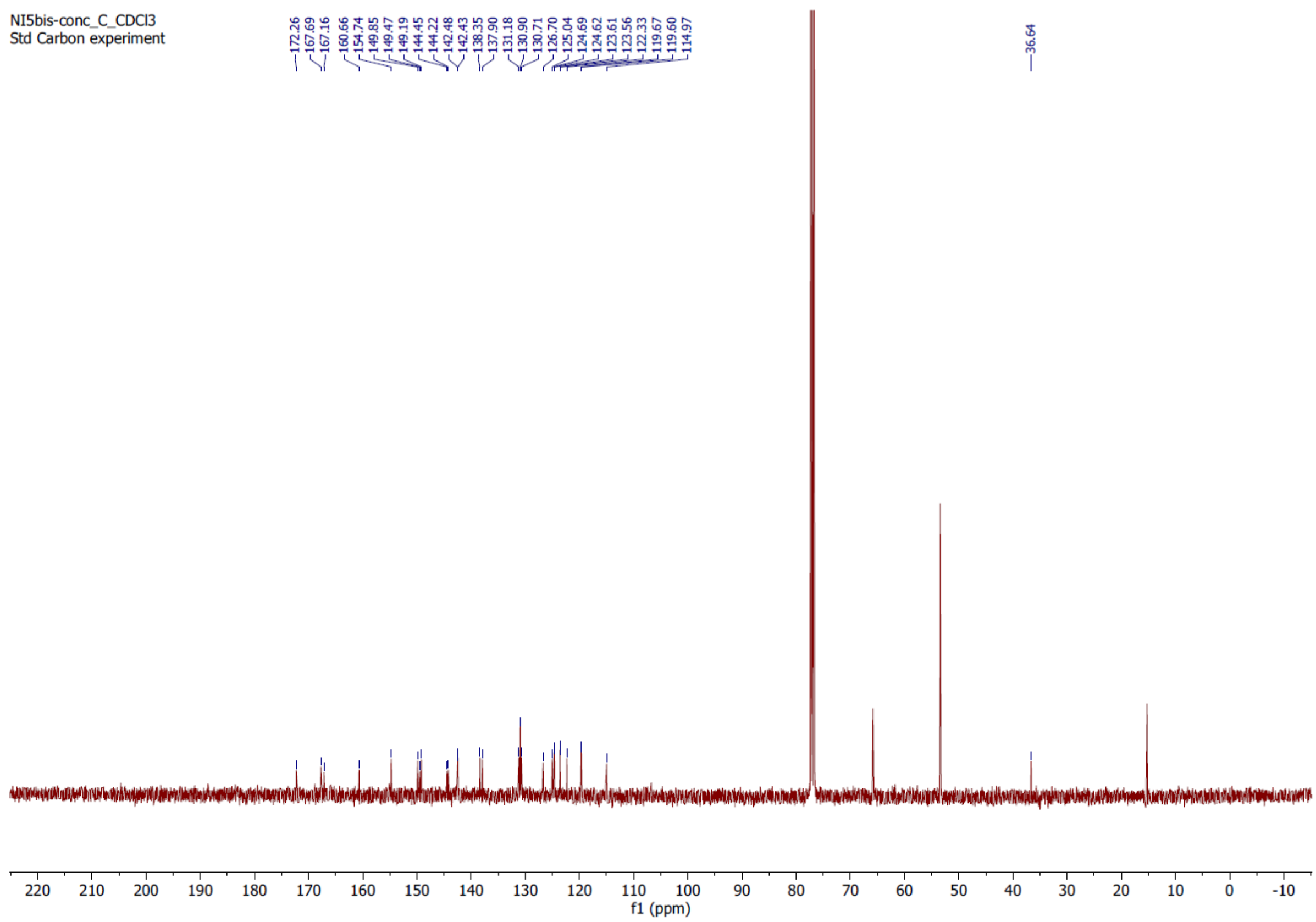

Figure S21. ^{13}C NMR spectrum of complex **C**.

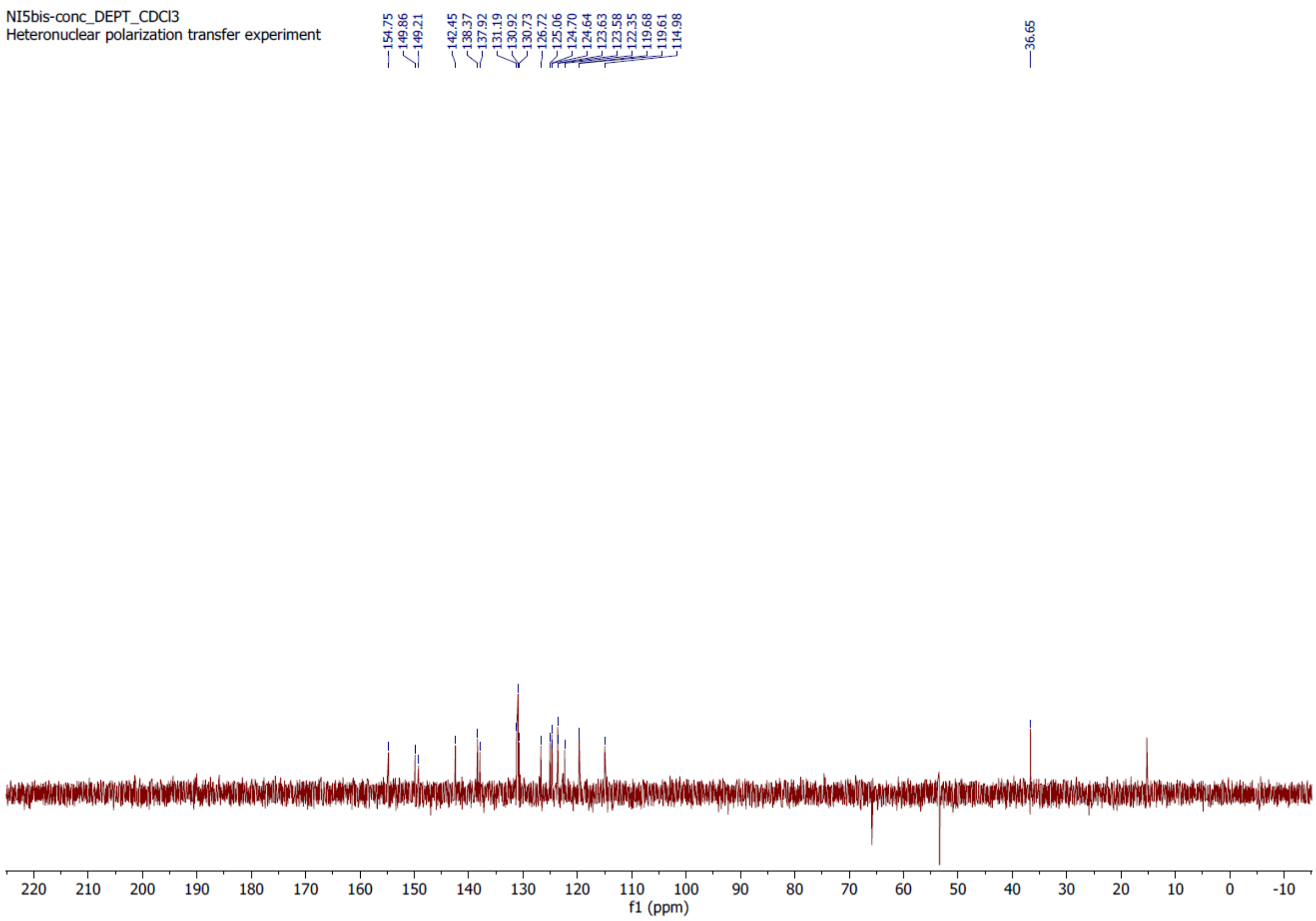

Figure S22. DEPT 135 NMR spectrum of complex **C**.

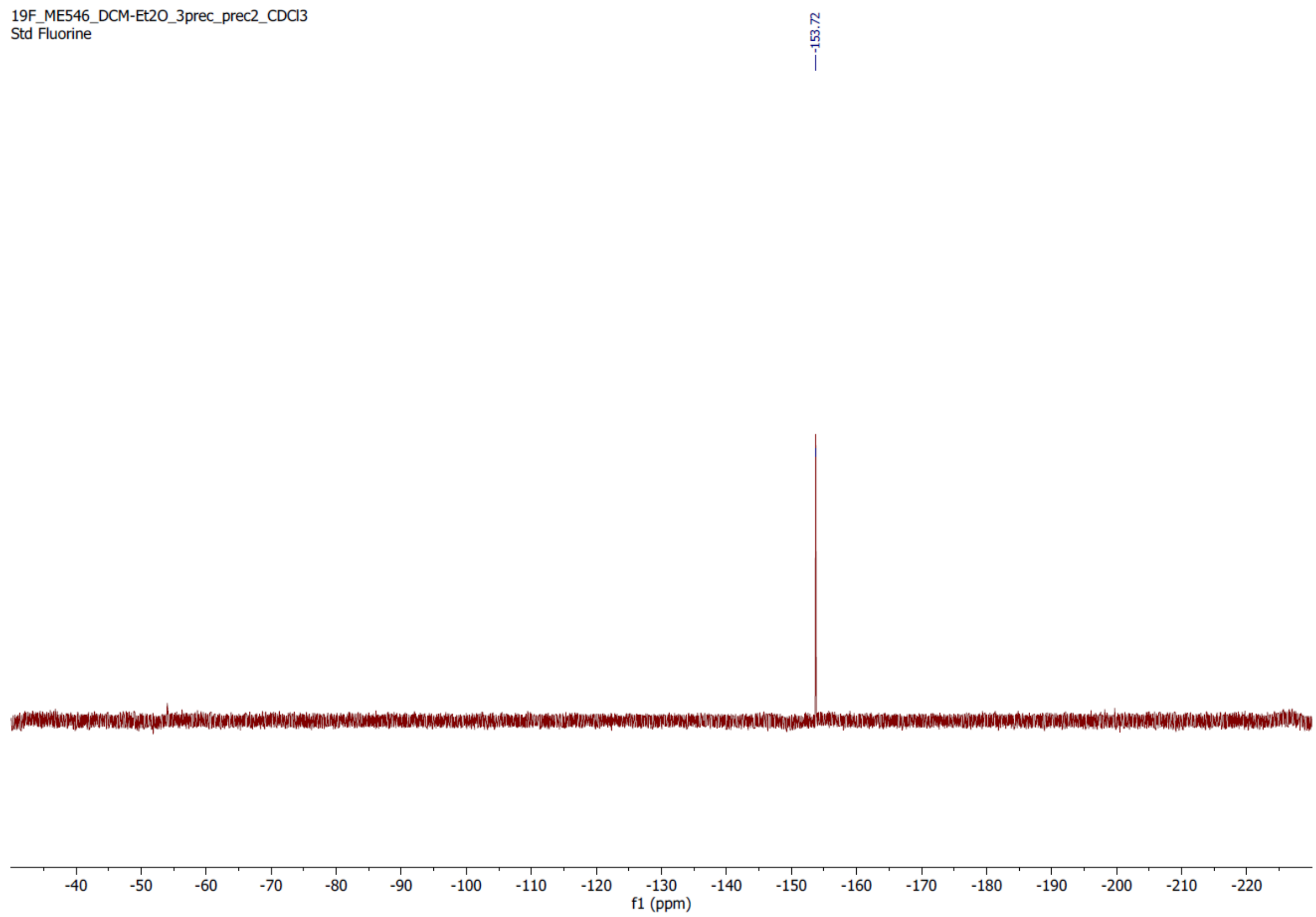

Figure S23. ^{19}F NMR spectrum of complex **C**.

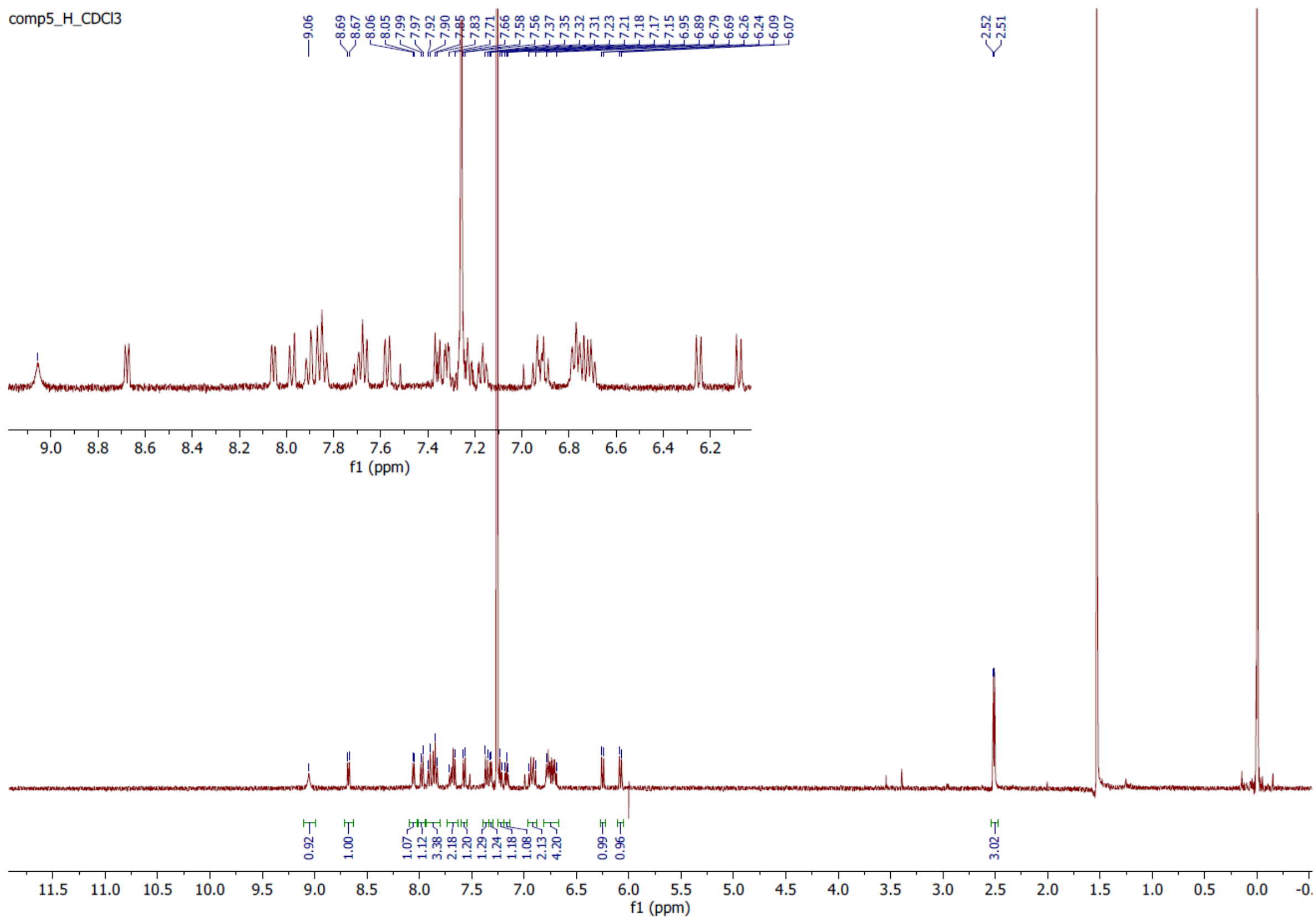

Figure S24. ^{1}H NMR spectrum of complex **D**.

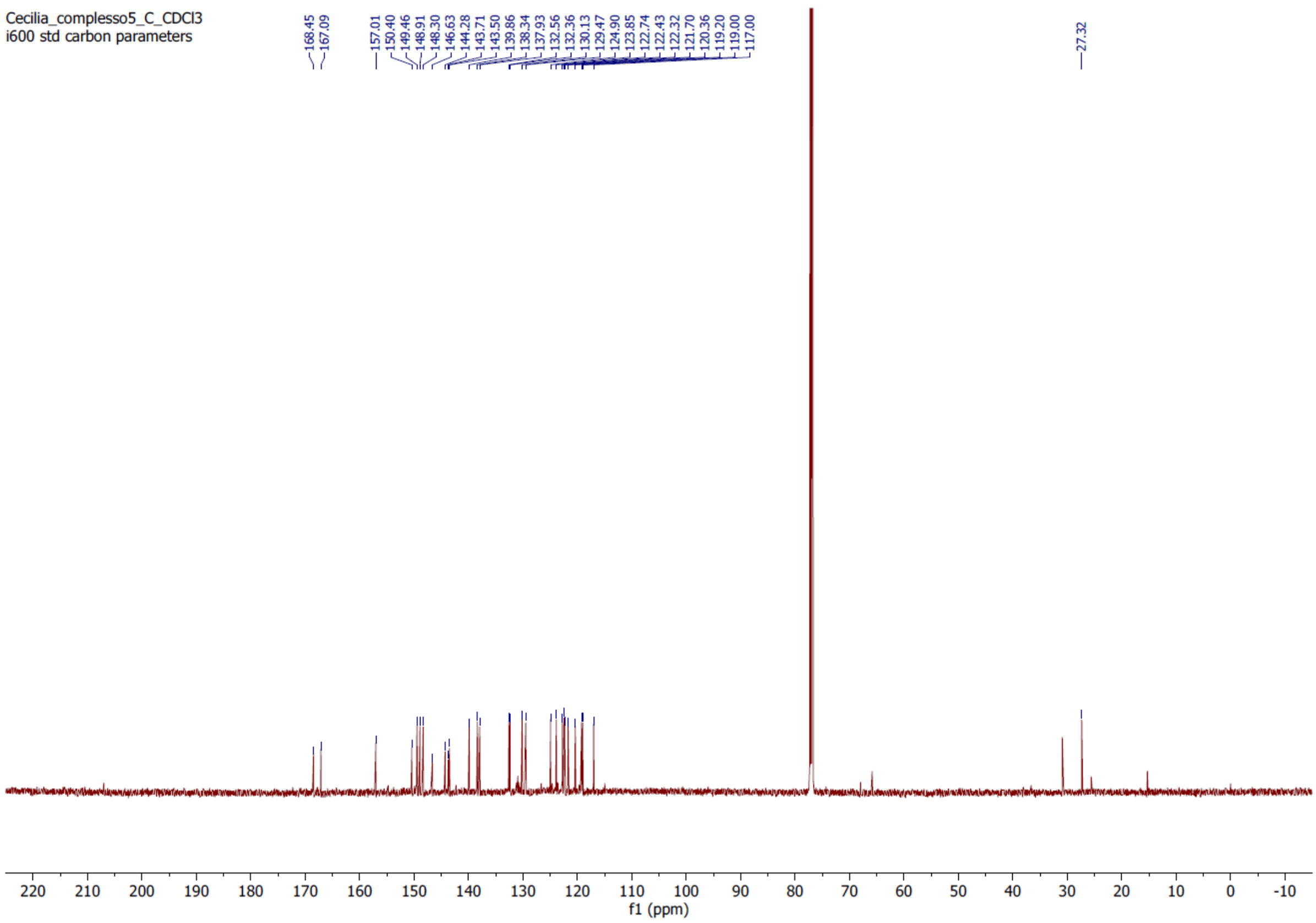

Figure S25. ^{13}C NMR spectrum of complex **D**.

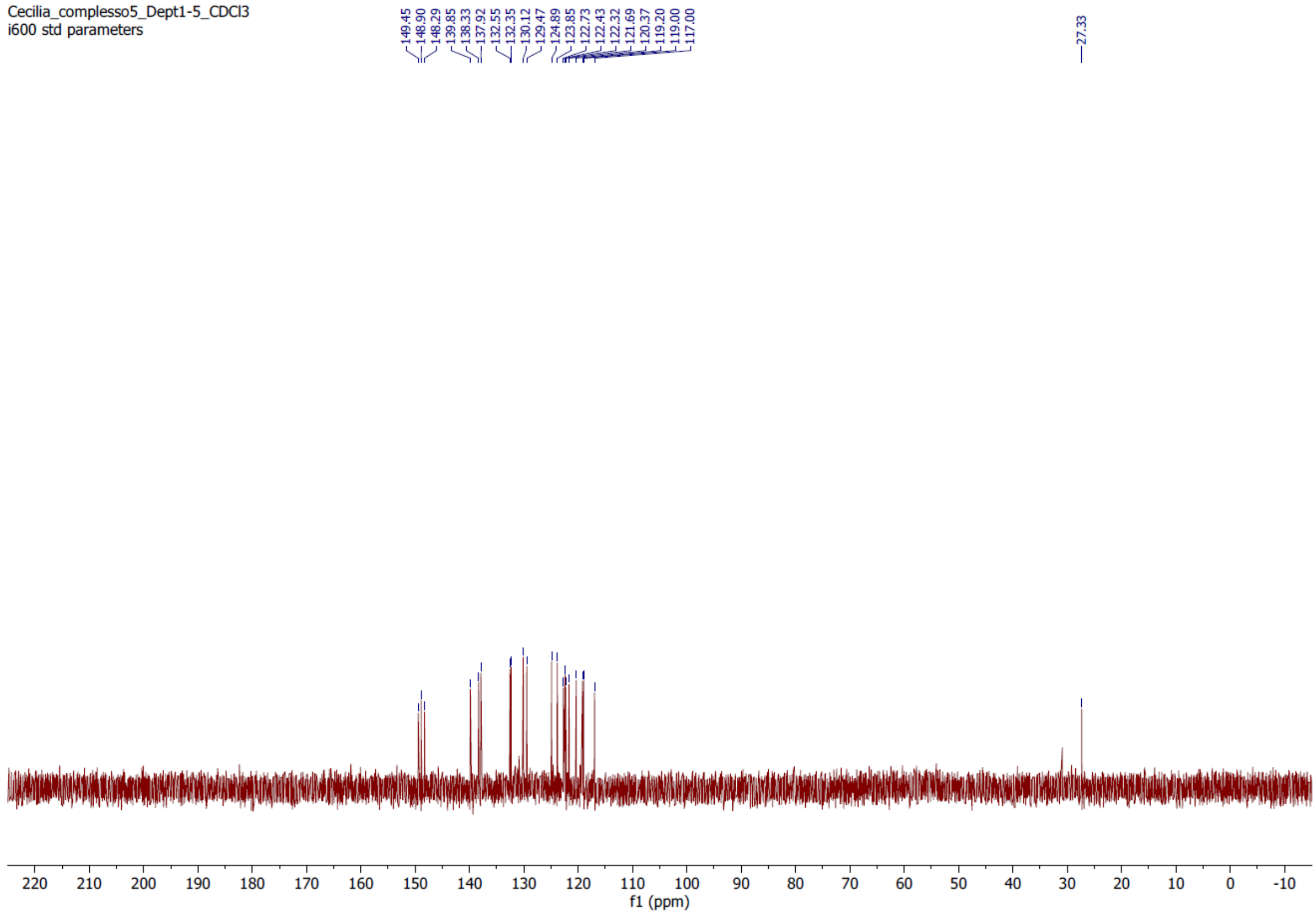

Figure S26. DEPT 135 NMR spectrum of complex **D**.

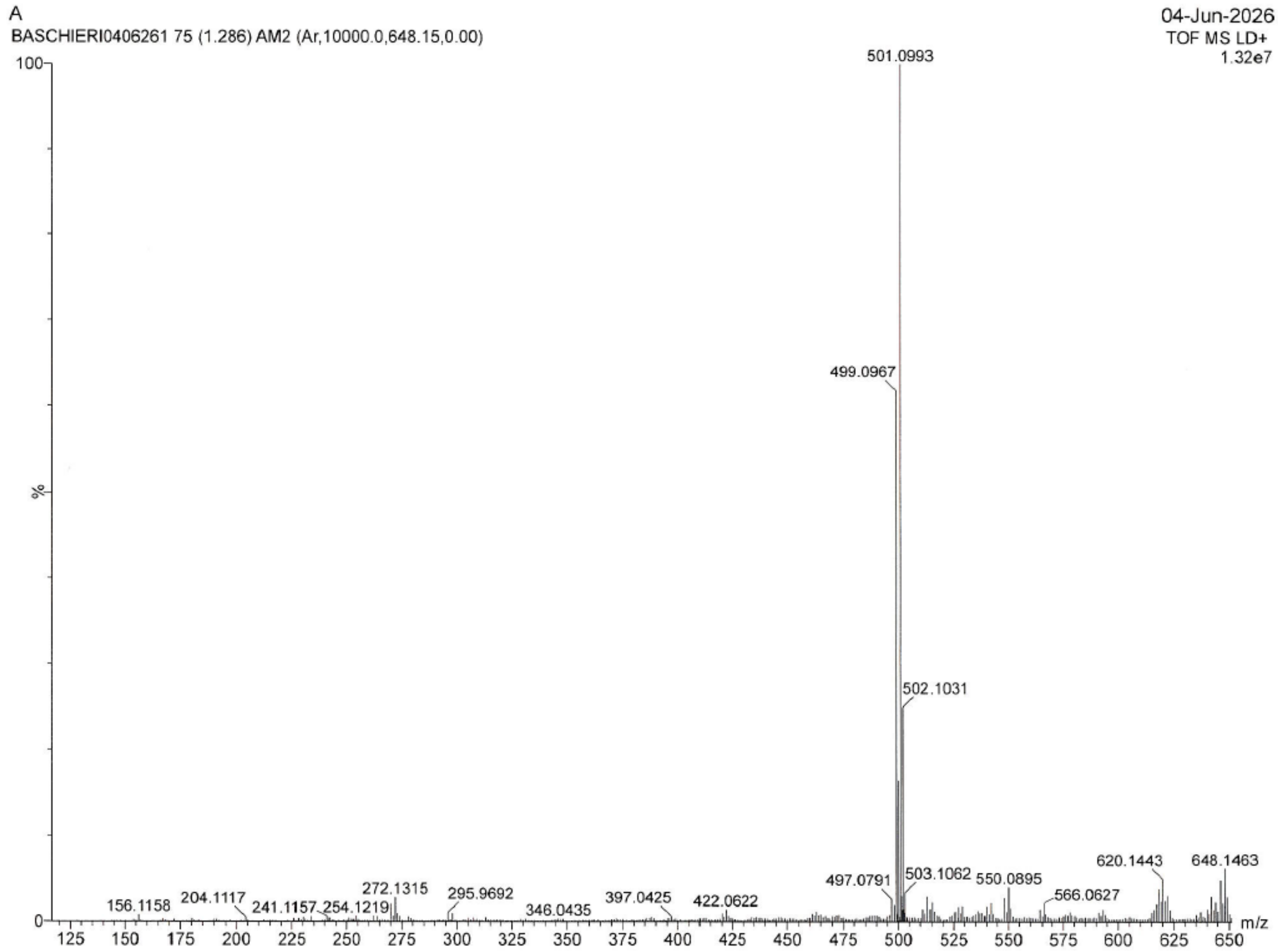

Figure S27. High-resolution mass spectrum of complex **A**.

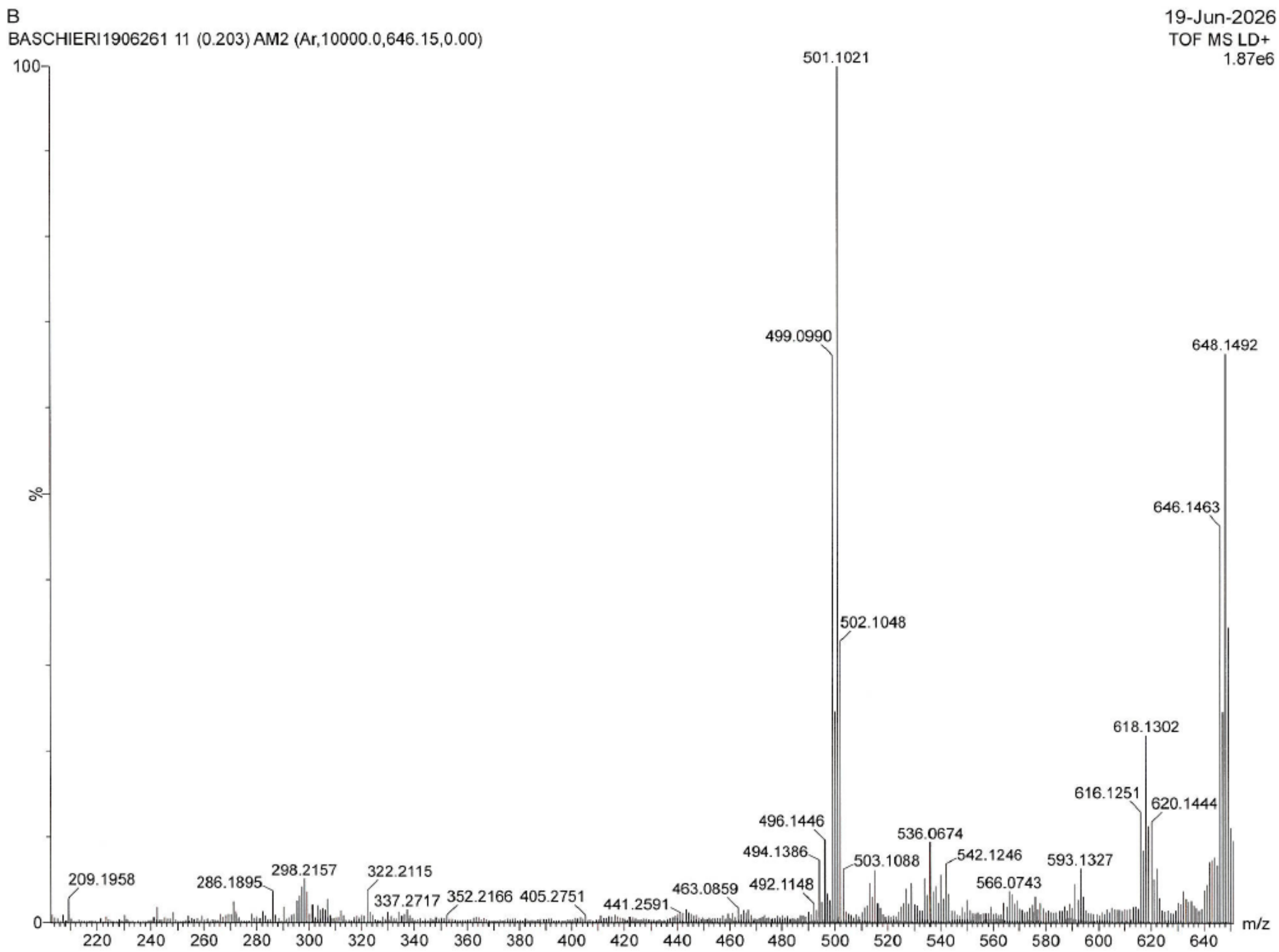

Figure S28. High-resolution mass spectrum of complex **B**.

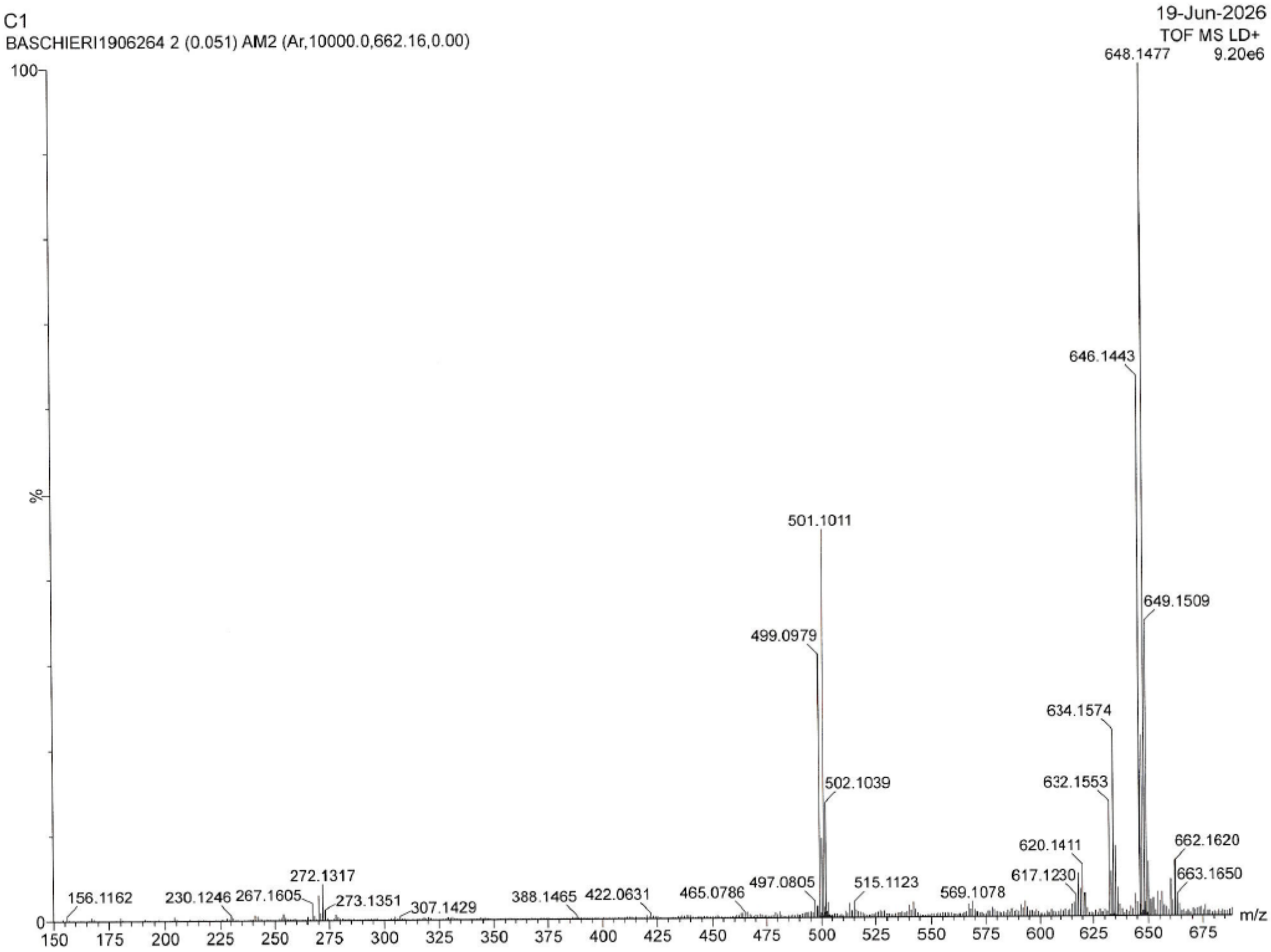

Figure S29. High-resolution mass spectrum of complex **C**.

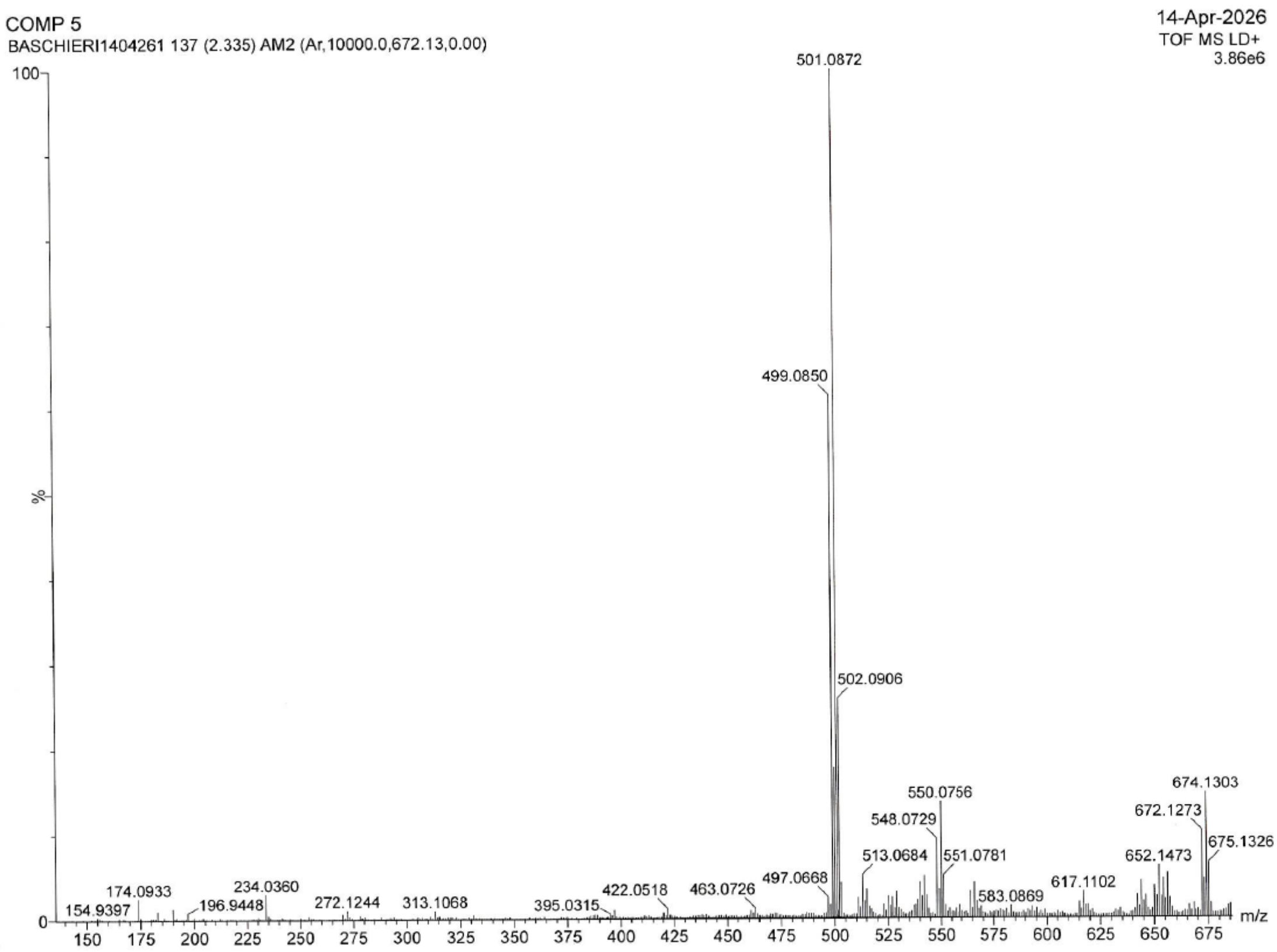

Figure S30. High-resolution mass spectrum of complex **D**.

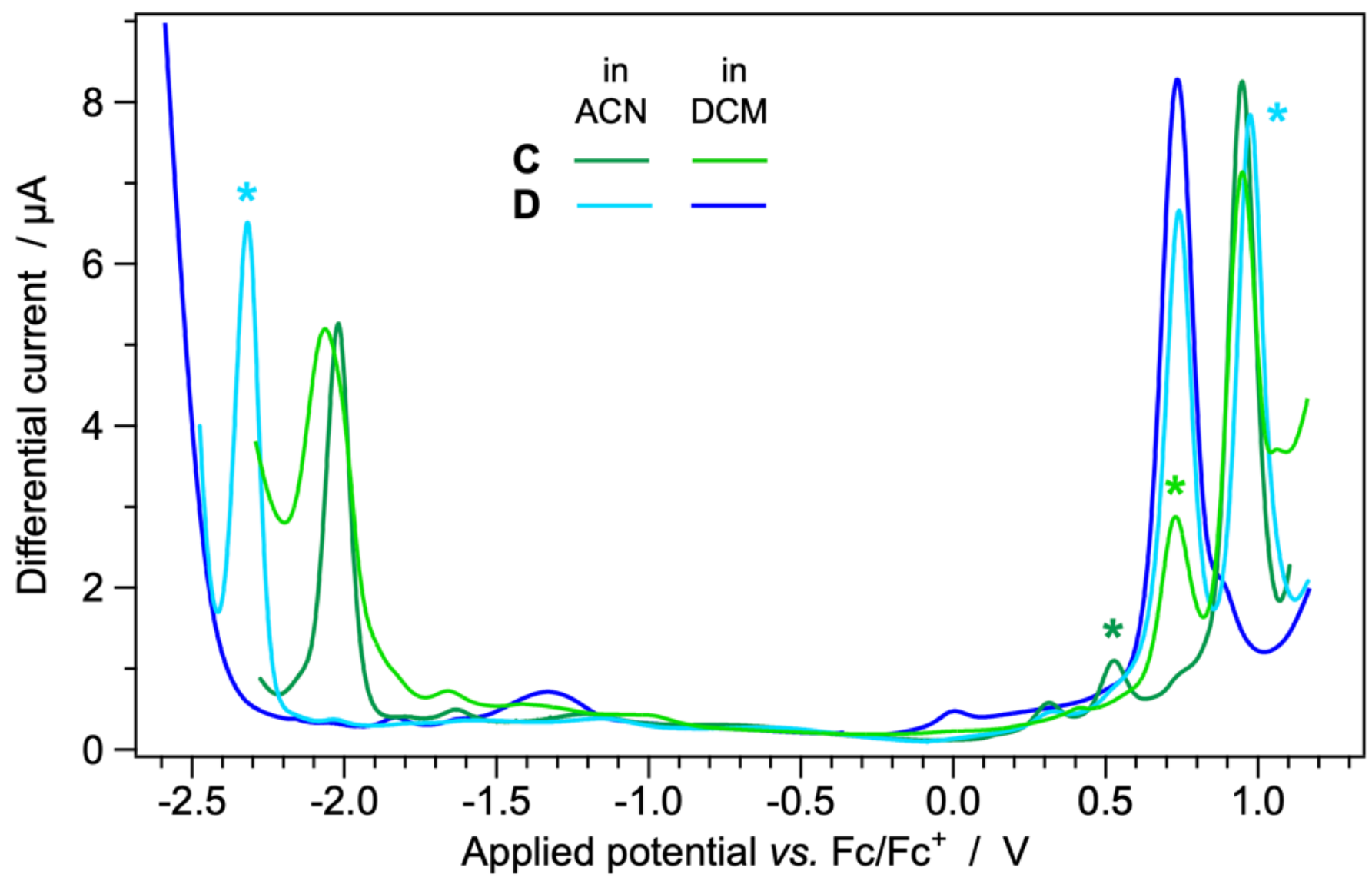

Figure S31. Square-wave voltammograms of complexes **C** and **D** (0.5 mM) recorded in acetonitrile and dichloromethane solution at 298 K. Complex **C** shows minor signs of degradations in both solvents, particularly at positive potentials (marked with green asterisks); conversely, complex **D** shows a clean voltammogram in dichloromethane solution, while additional peaks are observed in acetonitrile due to complex instability in the highly coordinative acetonitrile solution (cyan asterisks).

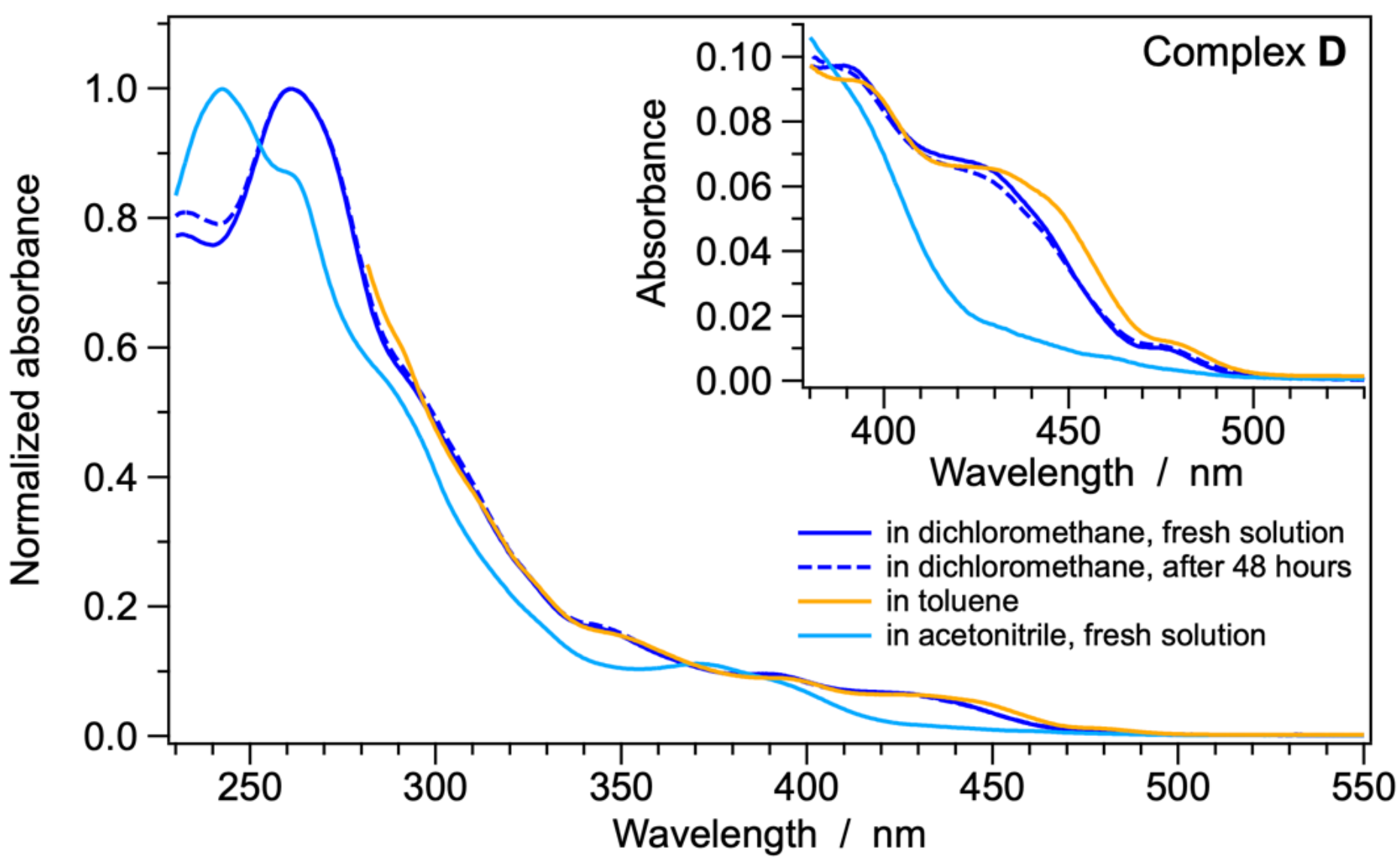

Figure S32. Normalized absorption spectra of complex **D** in different solvents. The absorption spectrum of **D** in acetonitrile lacks absorption features at longer wavelengths (see inset), suggesting possible dissociation of the ancillary ligand due to the coordinating ability of such solvent. On the contrary the spectrum of **D** in dichloromethane is fundamentally superimposable to the one recorded in toluene (an apolar, non-coordinating and acid-free solvent).

Table S1. Calculated NTOs couples describing the triplet excitations below 3.20 eV for **A** in acetonitrile (see Experimental Section for details). The λ value is the natural transition orbital eigenvalue associated with each NTOs couple; orbital isovalue: 0.04 $e^{-1/2}$ $bohr^{-3/2}$.

	Transition energy [eV (nm)]	NTO couple hole → electron (λ)	Nature
$S_0 \rightarrow T_1$	2.53 (490)	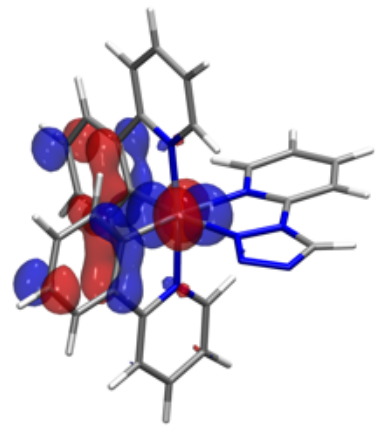 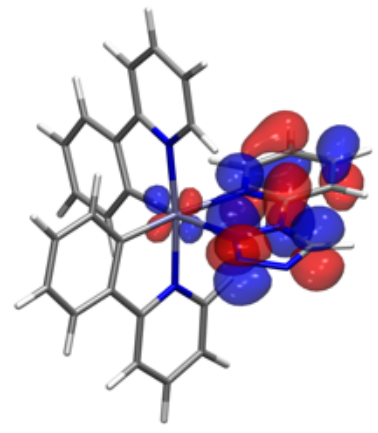 (99.8%)	Mainly MLCT from the iridium center to the ancillary ligand
$S_0 \rightarrow T_2$	2.76 (449)	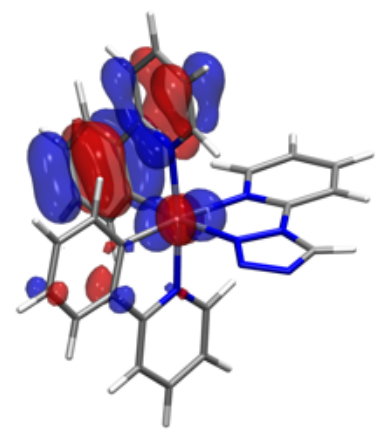 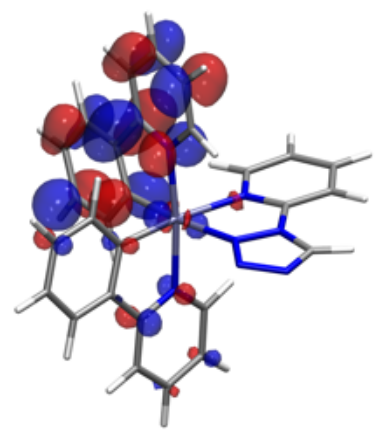 (70.4%)	Mainly LC on the cyclometallating ppy ligands
$S_0 \rightarrow T_3$	2.79 (444)	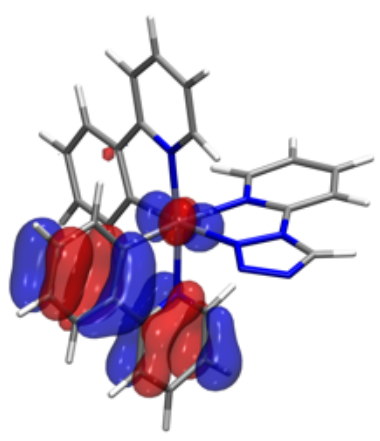 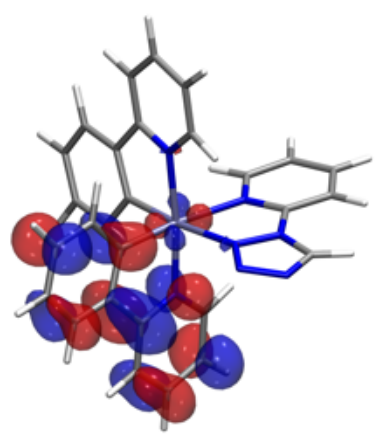 (65.3%)	Mainly LC on the cyclometallating ppy ligands
$S_0 \rightarrow T_4$	3.00 (414)	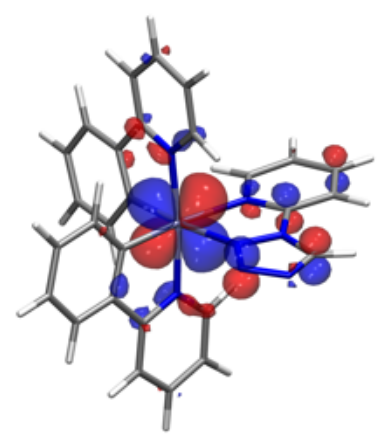 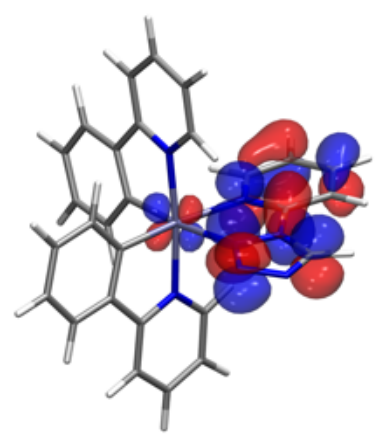 (96.7%)	MLCT from the iridium center to the ancillary ligand
$S_0 \rightarrow T_5$	3.07 (404)	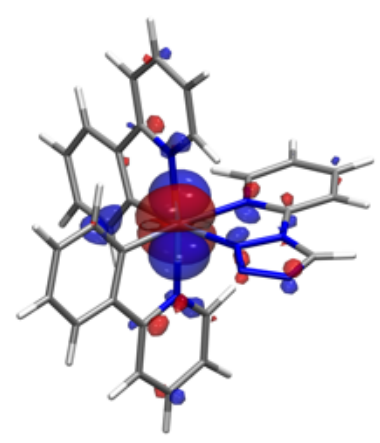 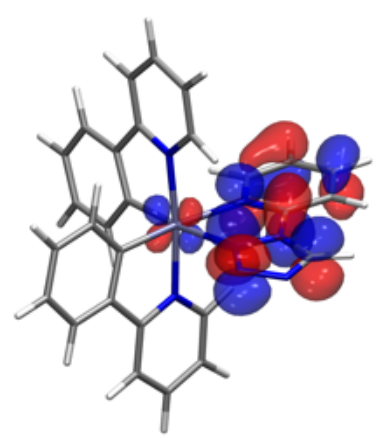 (97.6%)	MLCT from the iridium center to the ancillary ligand

Table S2. Calculated NTOs couples describing the triplet excitations below 3.20 eV for **B** in acetonitrile (see Experimental Section for details). The λ value is the natural transition orbital eigenvalue associated with each NTOs couple; orbital isovalue: 0.04 $e^{-1/2}$ $bohr^{-3/2}$.

	Transition energy [eV (nm)]	NTO couple hole → electron (λ)	Nature
$S_0 \rightarrow T_1$	2.73 (454)	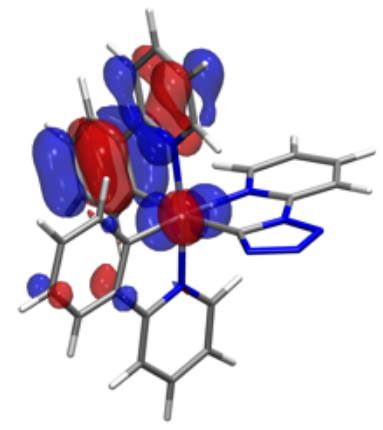 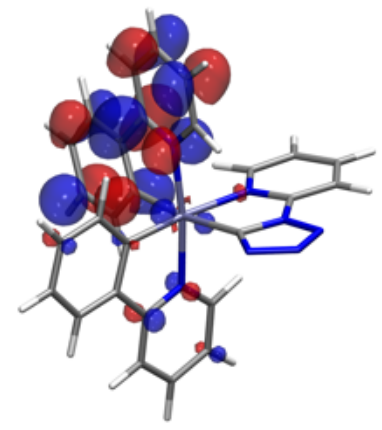 (79.4%)	Mainly LC on the cyclometallating ppy ligands
$S_0 \rightarrow T_2$	2.77 (447)	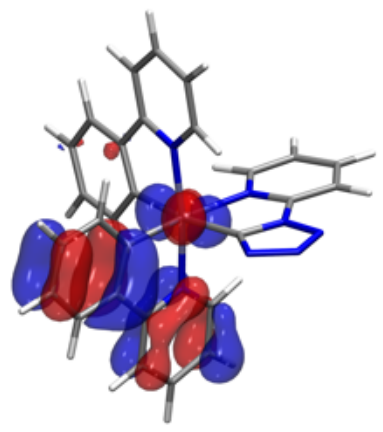 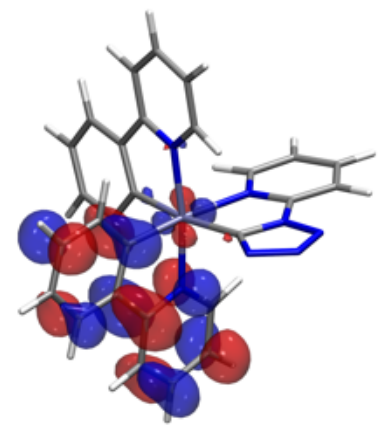 (74.8%)	Mainly LC on the cyclometallating ppy ligands
$S_0 \rightarrow T_3$	3.10 (400)	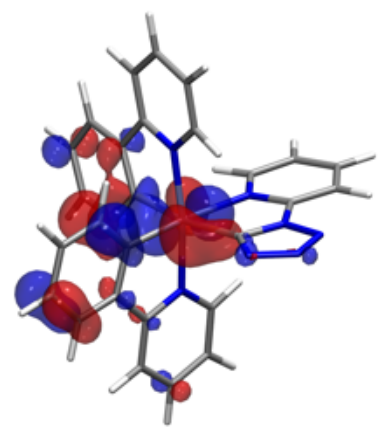 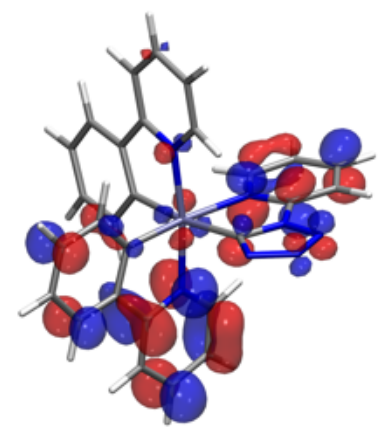 (69.0%)	Mixed LC/LLCT/MLCT character involving both the ppy and the ancillary ligand
$S_0 \rightarrow T_4$	3.12 (398)	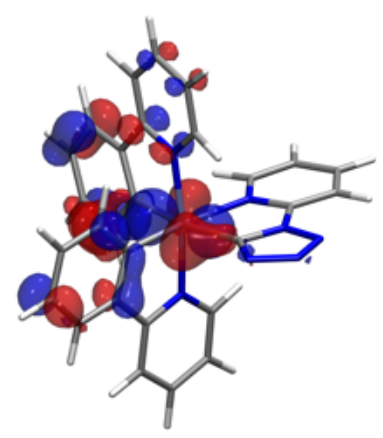 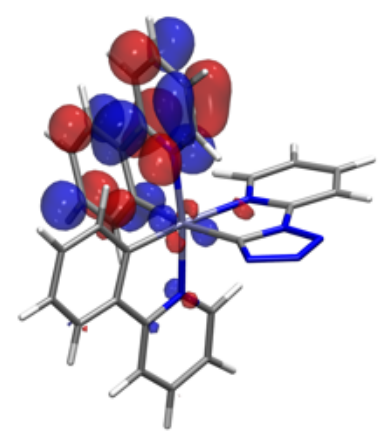 (72.3%)	Mixed LC/LLCT/MLCT character involving the ppy ligands
$S_0 \rightarrow T_5$	3.13 (397)	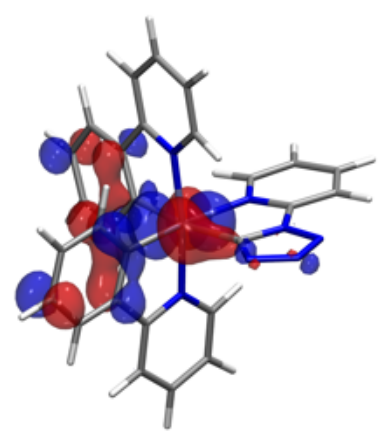 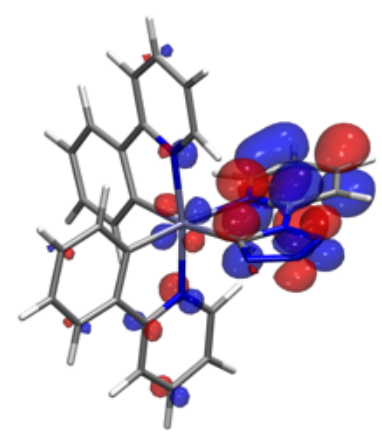 (83.0%)	Mixed MLCT/LLCT character involving the ancillary ligand

Table S3. Calculated NTOs couples describing the triplet excitations below 3.20 eV for **C** in acetonitrile (see Experimental Section for details). The λ value is the natural transition orbital eigenvalue associated with each NTOs couple; orbital isovalue: 0.04 $e^{-1/2}$ $bohr^{-3/2}$.

	Transition energy [eV (nm)]	NTO couple hole → electron (λ)	Nature
$S_0 \rightarrow T_1$	2.78 (446)	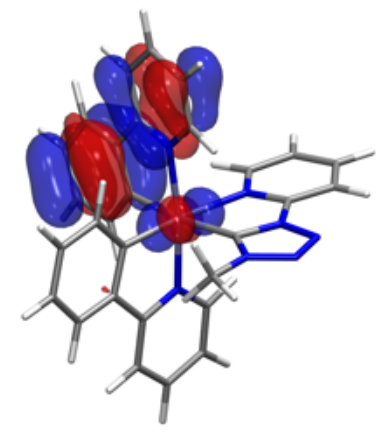 → 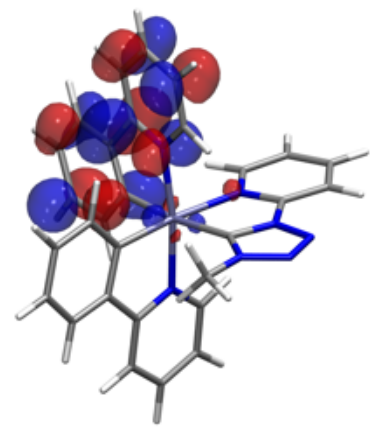 (82.3%)	Mainly LC on the cyclometallating ppy ligands
$S_0 \rightarrow T_2$	2.82 (440)	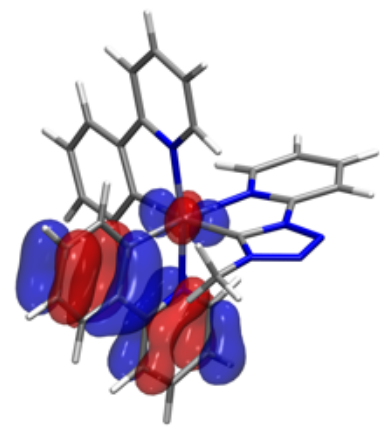 → 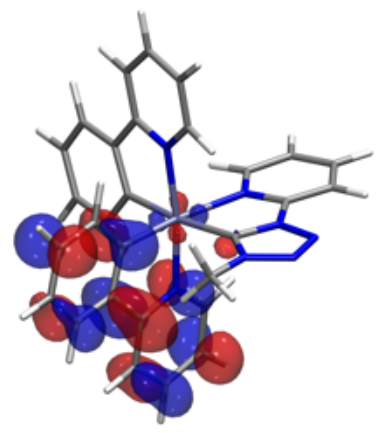 (80.1%)	Mainly LC on the cyclometallating ppy ligands
$S_0 \rightarrow T_3$	2.91 (426)	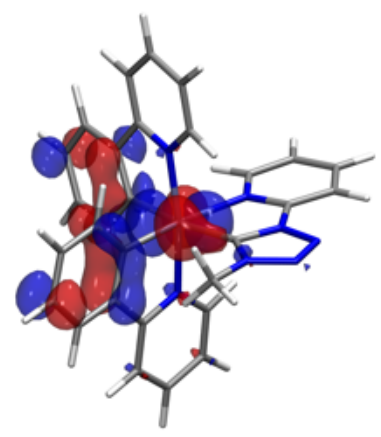 → 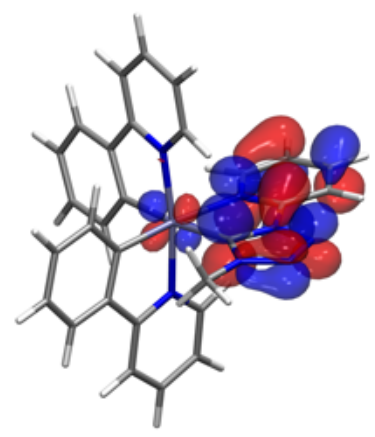 (95.3%)	Mainly MLCT from the iridium center to the ancillary ligand

Table S4. Calculated NTOs couples describing the triplet excitations below 3.20 eV for **D** in acetonitrile (see Experimental Section for details). The λ value is the natural transition orbital eigenvalue associated with each NTOs couple; orbital isovalue: 0.04 $e^{-1/2}$ $bohr^{-3/2}$.

	Transition energy [eV (nm)]	NTO couple hole → electron (λ)	Nature
$S_0 \rightarrow T_1$	2.68 (462)	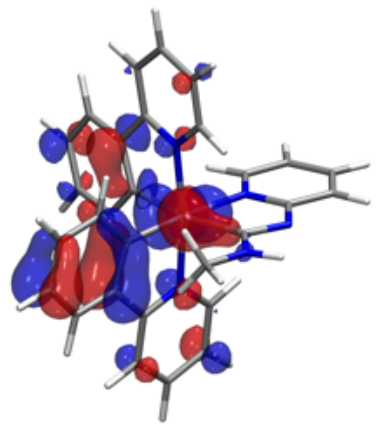 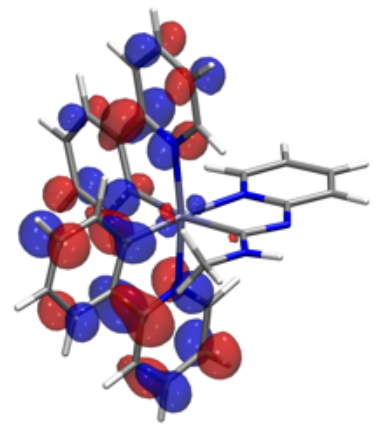 (79.1%)	Mainly LC on the cyclometallating ppy ligands
$S_0 \rightarrow T_2$	2.73 (455)	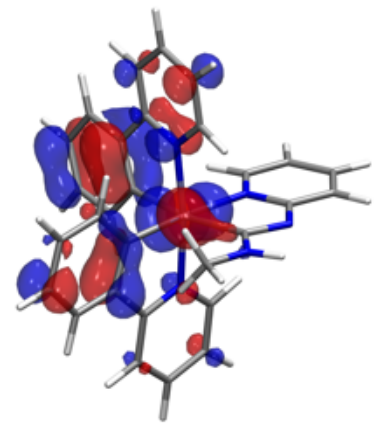 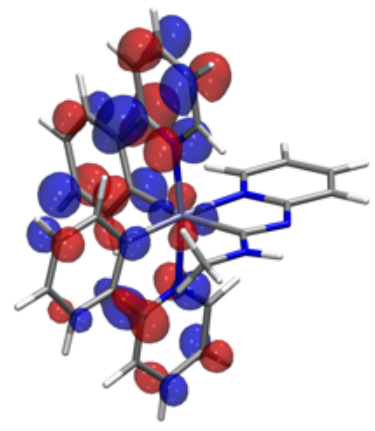 (68.6%)	Mainly LC on the cyclometallating ppy ligands
$S_0 \rightarrow T_3$	2.96 (418)	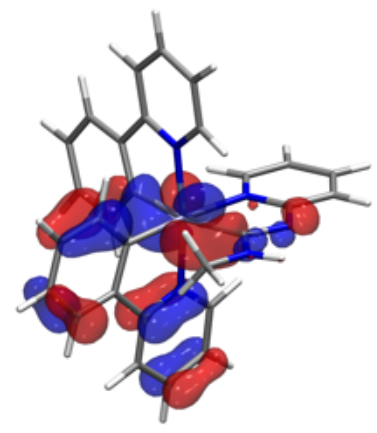 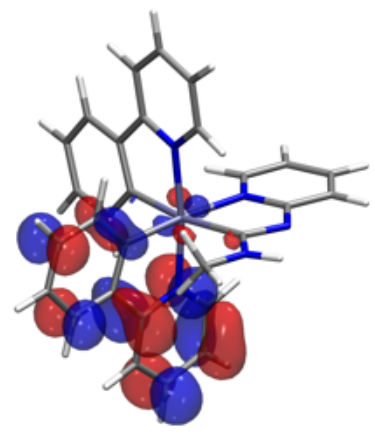 (78.3%)	Mainly LC on the cyclometallating ppy ligands
$S_0 \rightarrow T_4$	3.02 (411)	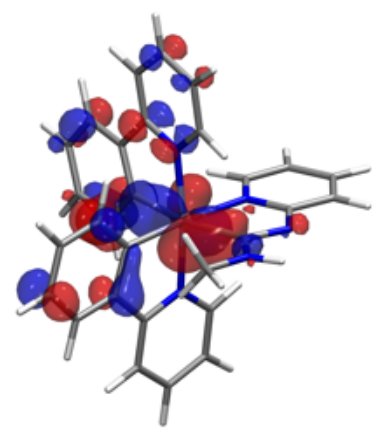 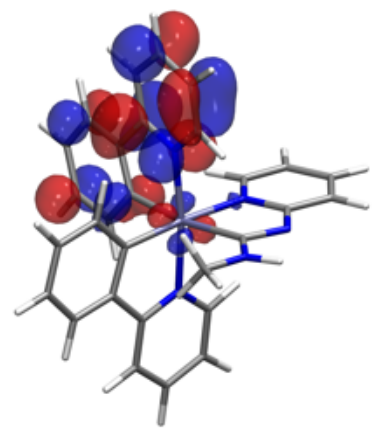 (86.0%)	Mainly LC on the cyclometallating ppy ligands
$S_0 \rightarrow T_5$	3.13 (396)	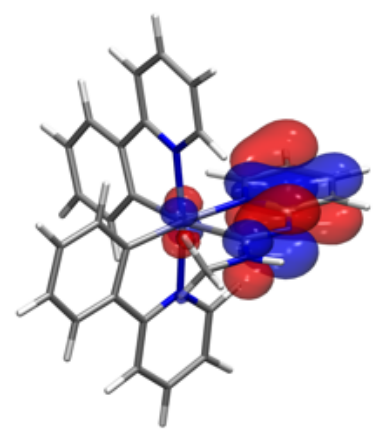 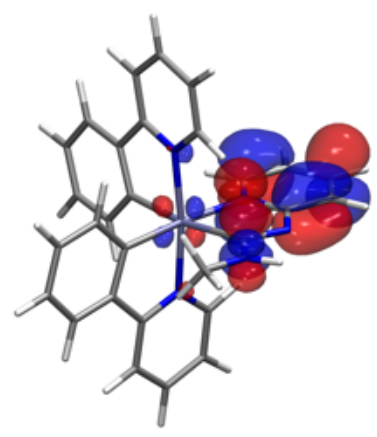 (97.0%)	LC on the ancillary ligand

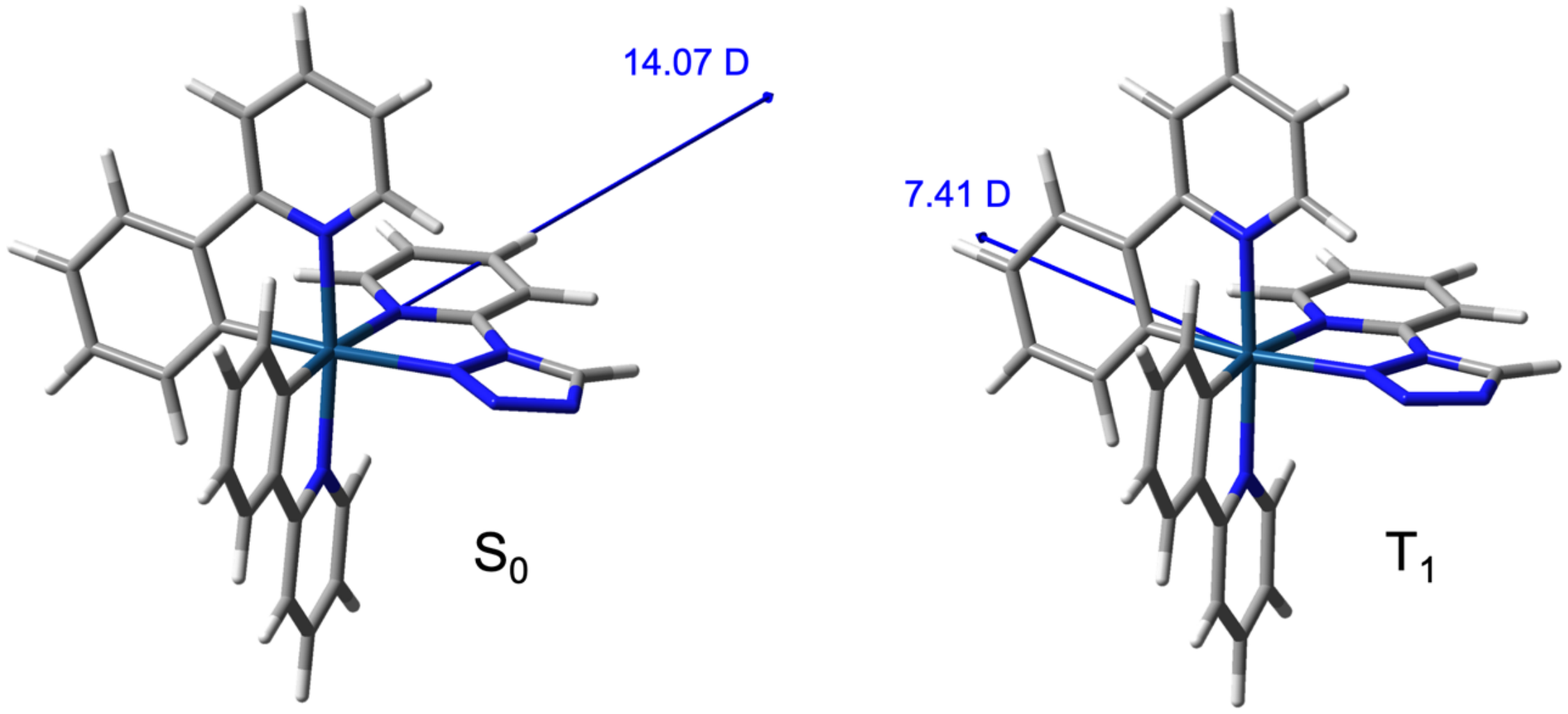

Figure S33. Permanent molecular dipole moment calculated at the M06 level of theory in acetonitrile (using PCM) for complex **A** in its ground-state minimum-energy geometry (S_0, left) or in the lowest triplet-state minimum (T_1, right). The excited state is less polar than the ground state. The depicted molecular dipole moment vectors follow the physics convention (*i.e.*, they point from negative to positive charge regions).

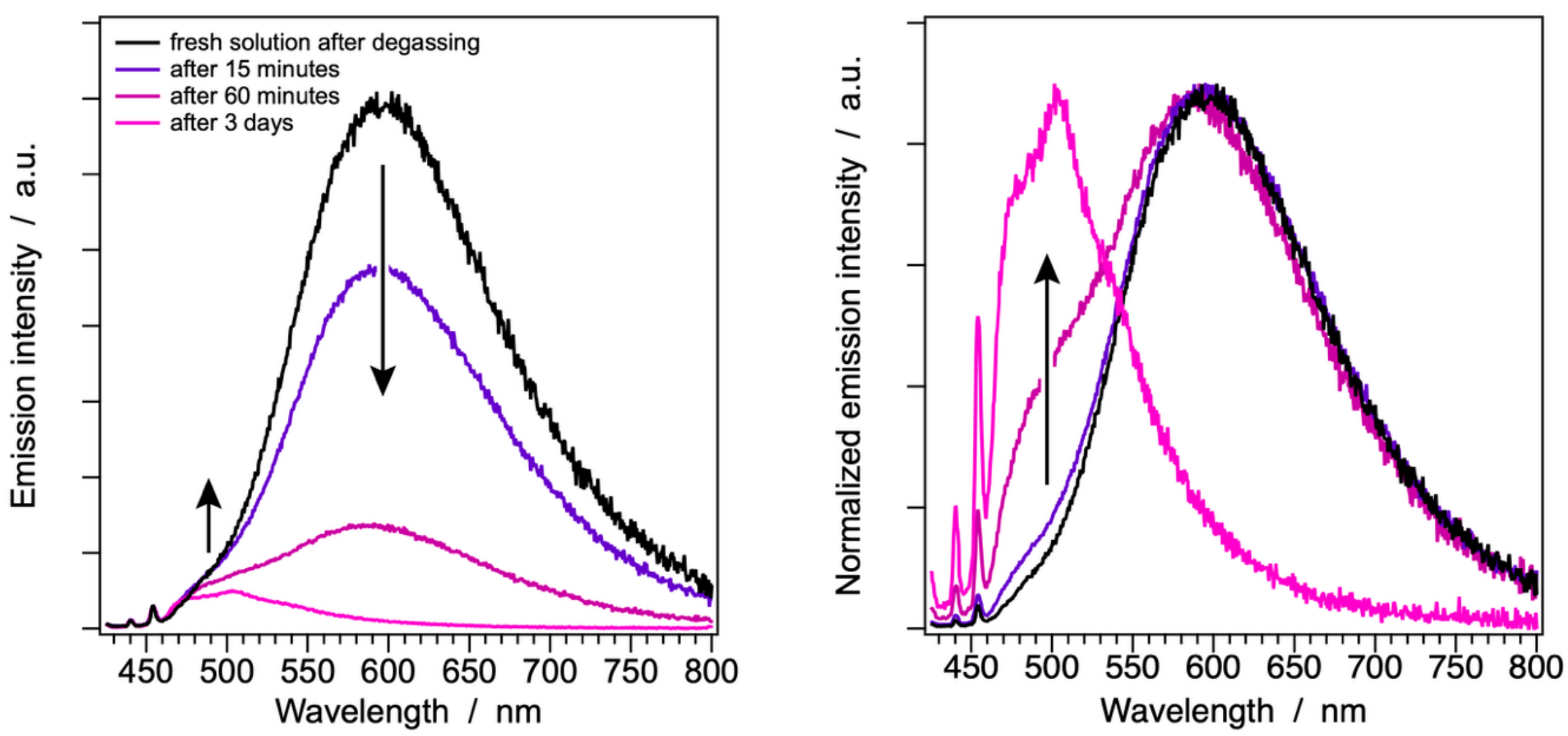

Figure S34. Time evolution of the emission spectra of complex **A** (λ_{exc} = 400 nm) in oxygen-free acetonitrile solution at room temperature (left); the same spectra are also normalized to compensate for the remarkable intensity reduction over time (right). The characteristic ^{3}MLCT emission band of complex **A**, peaking at approx. 600 nm, disappears over time and a very weakly emissive structured band appears at approx. 500 nm, probably due to the decoordination of the N^N ancillary ligand, leading to a residual ^{3}LC emission centered on the ppy cyclometallating ligands. Accordingly, a reliable determination of the photoluminescence quantum yield of complex **A** in acetonitrile solution is prevented.

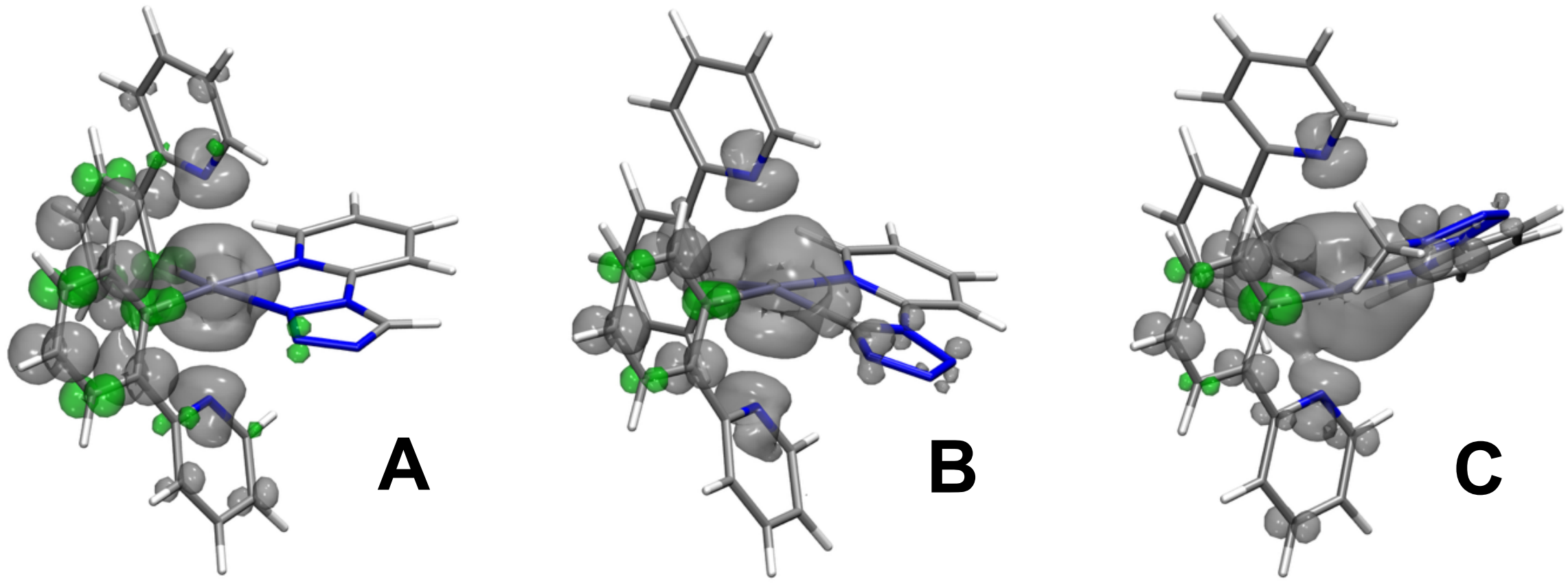

Figure S35. Spin-density distribution of the fully optimized axial ^{3}MC states in complexes **A**–**C**, involving the elongation of the axial Ir–N(ppy) bonds (isovalues: 0.002 e bohr^{-3}). In each complex, such axial ^{3}MC state is found approx. 0.5 eV above the highest lying equatorial ^{3}MC state (Figure 6). All data are computed in acetonitrile using PCM at the M06 level of theory.
